# 40th International Symposium on Intensive Care & Emergency Medicine 2020 – Part 2: e-ISICEM 2020 15-18 September 2020

**DOI:** 10.1186/s13054-020-03187-9

**Published:** 2020-09-14

## P513 Core warming as a treatment for coronavirus disease-2019 (COVID-19) - evaluation of the influence of lung density

### V Kostov^1^, M Mercado-Montoya^2^, N Bonfanti^3^, E Gundert^3^, AM Drewry^4^, R Bedimo^5^, K Kostov^6^, S Shah^7^, E Kulstad^8^

#### ^1^Walter Payton College Preparatory High School, Chicago, IL, United States; ^2^Universidad de Antioquia, Bioengineering Department, Medellín, Colombia; ^3^UT Southwestern Medical Center, Departments of Emergency Medicine and Anesthesia/Critical Care, Dallas, TX, United States; ^4^Washington University School of Medicine, Department of Anesthesiology, St. Louis, MO, United States; ^5^UT Southwestern Medical Center, Dallas, TX, United States; ^6^Life Science Angels, Chicago, IL, United States; ^7^Illinois Institute of Technology, Department of Bioengineering, Chicago, IL, United States; ^8^UT Southwestern Medical Center, Department of Emergency Medicine, Dallas, TX, United States

**Introduction:**

Elevated temperature may actually be helpful in enhancing immune function and improving outcomes from illnesses including sepsis, ARDS, and COVID-19. Increasing clinical data show fever may shorten illness duration, and a recent pilot randomized controlled trial suggests benefit from actively warming patients with sepsis. We evaluated the potential to provide core warming to patients using a commercially available heat transfer device, focusing on the effect of lung density on regional body temperature.

**Methods:**

Using Comsol Multiphysics, we modeled heat transfer in the body from the device, taking into account ventilator airflow. We considered a patient with initial body temperature of 38°C. The simulation was performed on a simplified geometry of a human body and airway from the pharynx to the lungs. The simulations used a fixed value of blood perfusion rate obtained from prior modeling, and a range of lung density seen in patients with varying degrees of ARDS.

**Results:**

Heat diffuses from the device by conduction and convection to the nearby tissues, including the air flowing in the airways (Figure 1). Skin surface is at a lower temperature than the core due to convective cooling in a hospital environment. At the range of blood perfusion modeled, maximum lung temperature ranged from 37.6°C to 38.6°C. The results suggest that changes in the lung density due to ARDS do not impact heat transfer significantly or affect ability to heat the lungs. The average lung plus air temperature was found to be close to 38.02°C with the peak temperature close to 38.4°C.

**Conclusions:**

The provision of core warming via commercially available technology can increase regional temperature of lung tissue and airway passages over a range of lung density to treat conditions increasingly being shown to benefit from hyperthermia, including sepsis, ARDS, and likely also COVID-19. Clinical study is ongoing.

**Fig. 1 (abstract P513).**
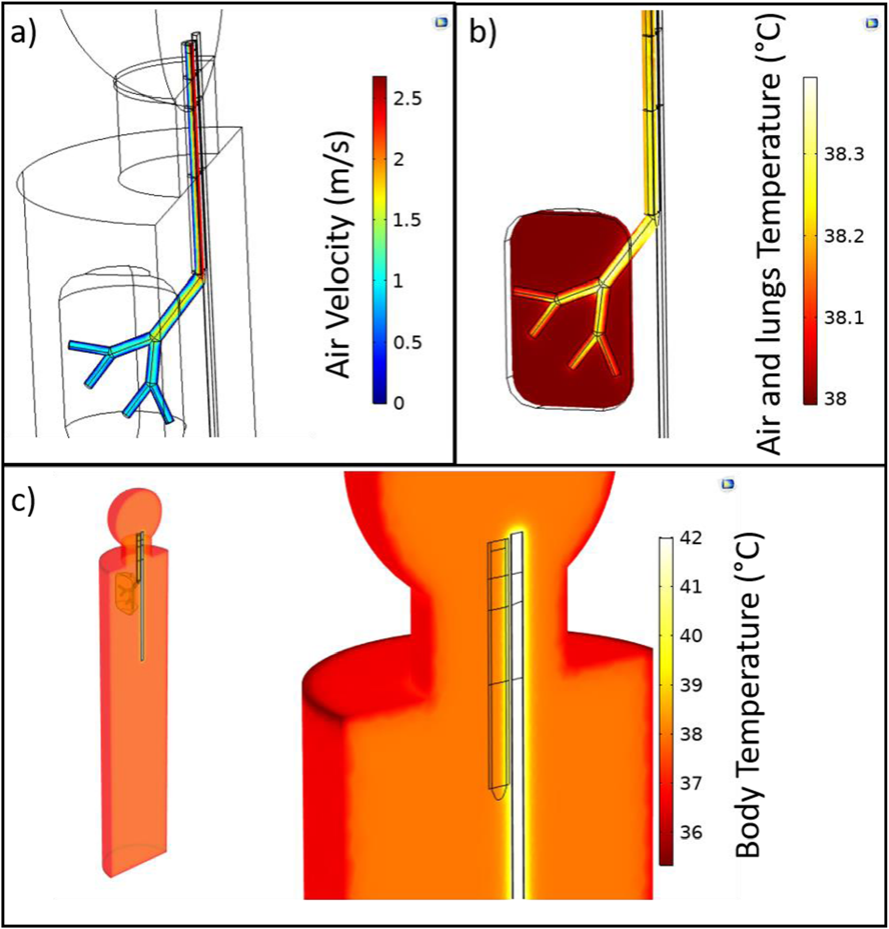
Stationary graphical results. a) Air velocity in the airways, b) Temperature in the airways+lungs and c) Body temperature

## P514 Luck, knowledge and strength facing SARS-CoV-2. The Veronese experience

### K Donadello^1^, L Gottin^2^, P Zanatta^3^, V Schweiger^2^, M Taiana^4^, F Romagnosi^3^, M Ceola Graziadei^5^, R Pettiti Boetti^2^, E Polati^2^

#### ^1^University of Verona, School of Medicine, Anesthesia and Intensive Care B, Department of Surgery, Dentistry, Gynecology and Pediatrics, Verona, Italy; ^2^University of Verona, School of Medicine, Verona, Italy; ^3^AOUI, University Hospital Integrated Trust of Verona, Anesthesia and Intensive Care A, Verona, Italy; ^4^AOUI, University Hospital Integrated Trust of Verona, Anesthesia and Intensive Care B, Verona, Italy; ^5^AOUI, University Hospital Integrated Trust of Verona, Cardio-Thoracic Anesthesia and Intensive Care, Verona, Italy

**Introduction:**

On Feb 20, 2020, the first patient with COVID-19 in Italy developed respiratory failure and was admitted to the ICU. Strong from Chinese experience and with 2 week-gap from Lombardy, we organized our hospital (AOUI of Verona, Italy) from 60 to 114 ICU beds. We aimed to describe our first COVID-19 critically ill patients.

**Methods:**

Prospective observational study of all COVID-19-confirmed critically ill patients, treated at our ICUs between 6 to 29 March, 2020. Date of final follow-up was April 26, 2020. Demographic and daily clinical data were collected, including data on organ failure, management and outcome.

**Results:**

Of the 95 pts included, the median age was 65(56.5-70) yrs and 78 (82%) were male. 57(54%) had cardiovascular disorders, 18 (19%) were obese, 17 (18%) had dyslipidemia, 13 (14%) had diabetes, 15 (16%) were current smokers, 12 (13%) had COPD; 27 (28%) patients were admitted from the ED, 47 (49%) from a medical ward and 21 (22%) were transferred from another hospital. At ICU admission, APACHE II and SOFA scores were 23(15-29) and 8(5-11), respectively; lymphocytopenia was present (0.5 [0.1-0.8] 109/l); CRP was 137(61-194) mg/l, PCT was 0.3 (0.2-0.8) ng/ml. Total CPK, LDH and D-Dimer were 131(77-339) U/l, 421(311-505) U/l and 1265 (570-2032), respectively; arterial lactate was 1.2 (0.9-1.6) mmol/l. P/F at baseline was 81 (65-118); 90 pts (94%) received invasive mechanical ventilation and after tracheal intubation P/F was 115 (98-149], TV was 495 (450-500) ml, RR was 17.5 (15-20) apm, PEEP was 11 (10-12) cmH_2_O, PPlat was 20 (18-22) cmH_2_O, DP was 9 (8-10) cmH2O and compliance was 47.25 ml/cmH_2_O. 2 pts (2.1%) were treated with Sildenafil, 5(5.3%) received iNO and 3 (3.2%) needed ECMO. All received hydroxychloroquine, 87 (92%) lopinavir–ritonavir, 8 (8.4%) remdesivir, 14 (15%) tocilizumab. On D1, 82 (86%) pts were under antibiotic therapy. 28 day ICU mortality was 17.8%. ICU LOS was 14(2-40) for survivors and 6(1-28) for non-survivors.

**Conclusions:**

Among COVID-19 a large proportion required mechanical ventilation and ICU mortality was 17.8%.

## P515 Withdrawn

## P516 Time-course of immune profile in ICU patients with COVID-19: preliminary results

### AC Hernandez Padilla^1^, R Jeannet^2^, TD Daix^3^, R Formento^4^, AL Fedou^5^, G Gilbert^5^, B Evrard^5^, P Vignon^6^, J Feuillard^4^, B François^6^

#### ^1^CHU Dupuytren, Inserm CIC 1435 & UMR 1092, Limoges, France; ^2^CHU Dupuytren, UMR CNRS 7276, Inserm 1262 & Inserm CIC 1435, Limoges, France; ^3^CHU Dupuytren, Inserm CIC 1435/Réanimation Polyvalente, Limoges, France; ^4^CHU Dupuytren, UMR CNRS 7276, Inserm 1262, Limoges, France; ^5^CHU Dupuytren, Réanimation polyvalente, Limoges, France; ^6^CHU Dupuytren, Réanimation polyvalente & Inserm CIC 1435 & UMR 1092, Limoges, France

**Introduction:**

Lymphopenia appears to characterize COVID-19 [1]. Its depth on admission seems associated with prognosis [2], and exposes COVID-19 patients to secondary infections [3]. Current reports on immune response during severe COVID-19 are restricted to single cross sectional assessment on admission. Thus, we assessed the immune profile of patients admitted to the ICU for COVID-19-related ARDS and its evolution during the first week of stay.

**Methods:**

Prospective, observational study in consecutive patients admitted for COVID-19-related ARDS in our ICU. Peripheral blood was sampled on admission and on Day 3, 5 and 8 for immunophenotyping of lymphocytes (T, NK, B cells quantification, CD4+ and CD8+ T cells function), granulocytes (CD16- immatures granulocytes (IG), CD64+) and monocytes (mHLA-DR). Pro-inflammatory T cells cytokine production (IFN-ɣ, TNF-α, and IL-2) was assessed in 3 patients and 3 controls.

**Results:**

13 patients were included (8 men (62%); 72 [64–76] y/o; 8 severe ARDS (62%)). All exhibited deep global lymphopenia that persisted to D7 (Fig 1). Increased proportion of regulatory T cells correlated with defective production of IFN-ɣ by CD4+ T cells. Accordingly, effector CD4+ frequencies were decreased; those of effector memories CD8+ and HLA-DR+, CD38+ activated CD8+ were increased. Increased number of granulocytes was observed with a rise of CD64 expression; IG were barely detected at admission. Monocyte counts were also increased with a major HLA-DR down regulation.

**Conclusions:**

Patients with COVID-19 showed sustained alterations of immune response with deep and persistent global lymphopenia which was correlated to increased T cell exhaustion and increased non-functional antigen presenting cells. These preliminary data warranting further confirmation suggest that therapies boosting host immunity could be a path worth following for COVID-19.

**References**

1. Lin L et al. Emerg Microbes Infect 9:727-732, 2020

2. Chen G et al. J Clin Invest 130:2620-2629, 2020

3. Huang C et al. Lancet 395:497-506, 2020

**Fig. 1 (abstract P516).**
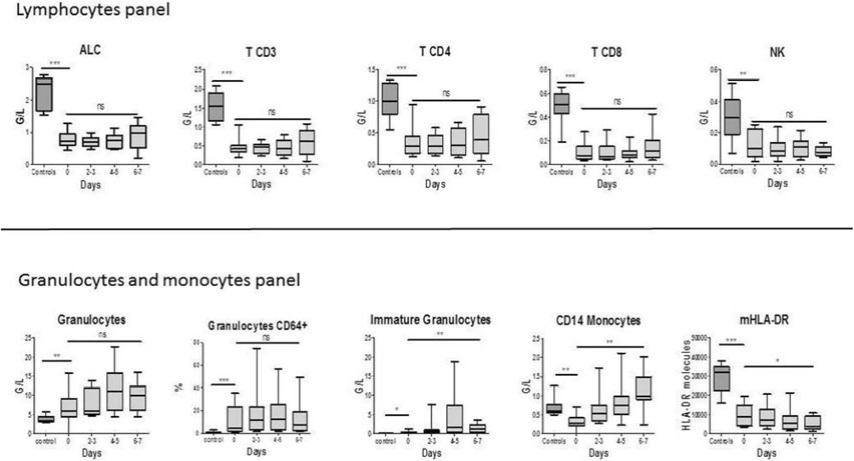
Lymphocyte, granulocyte and monocyte subsets evaluated by flow cytometry of 13 COVID-19 ICU patients. Box plot represent results for 10 healthy subjects (control) and for 13 COVID-19 ICU patients: Boxes give the median with the first and the third quartile. Whiskers represent min to max. Mann-Whitney test p-values are represented by ns, *, ** and *** for p>0.05, p <0.05, p<0.01 and p<0.001 respectively

## P517 Tracheostomy during COVID-19 in non-operating-room: a new approach by a multidisciplinary team

### M Cavagnino^1^, M Costa^1^, A Costa^1^, F Crimaldi^1^, R Guglielmetti^2^, F Brovelli^2^, M Farah Dell´Aringa^1^, R Vaschetto^1^, D Colombo^3^

#### ^1^Università del Piemonte Orientale, Translational Medicine Department, Novara, Italy; ^2^SS. Trinità Hospital - ASL Novara, Otorhinolaryngology Division, Borgomanero, Italy; ^3^SS. Trinità Hospital - ASL Novara, Anesthesia and Intensive Care Department, Borgomanero, Italy

**Introduction:**

Percutaneous dilatational tracheotomy (PDT) and surgical tracheostomy (ST) are documented techniques for patients undergoing prolonged mechanical ventilation and difficult weaning. During COVID-19 pandemia many patients require mechanical ventilation for several days/weeks and suffer prolonged weaning, making PDT/ST a possible option. Due to pandemia, setting issues became more complex arising several questions about safety both for operators and for patients. In this paper we describe the approach adopted in a “red zone” Italian Hospital during COVID-19 spread.

**Methods:**

PDT -Dolphin BT™ Ciaglia and/or Ciaglia Blue Rhino® sets- was programmed at patient's bed inside the COVID-ICU and patient was transferred to a surgical table and workstation was set up with essential surgical equipment (Figure 1). A multidisciplinary team was enrolled: an expert intensivist and ENT surgeon as 1st and 2nd operator respectively, performing PDT; another expert ENT surgeon managing airways and fibroscopy. Instrumental nurse and anesthesia nurse for airways assistance were involved, as they both were always in staff daytime to support ICU-nurse due to ICU beds surge.

**Results:**

To April 30th, we performed 22 procedures as described: 15 patients safely underwent PDT without any complication, in 3 patients we converted PDT to ST due to hemorrhagic complications (2) and anatomical complications (1). ST was performed in 4 patients due to PDT set shortage.

**Conclusions:**

This method allowed us to perform a potentially high-risk procedure safely for patient and operators, as setting was ready for rapid surgical conversion in case of need and operators were already completely equipped with individual protection devices and environmental protection devices. This approach was possible thanks to ENT surgeons increased availability due to elective operating room shut down, in addition, it saved one intensivist maintaining the highest safety profile. We believe that this model can be successfully used in other contexts similar to COVID-19 pandemia.

**Fig. 1 (abstract P517).**
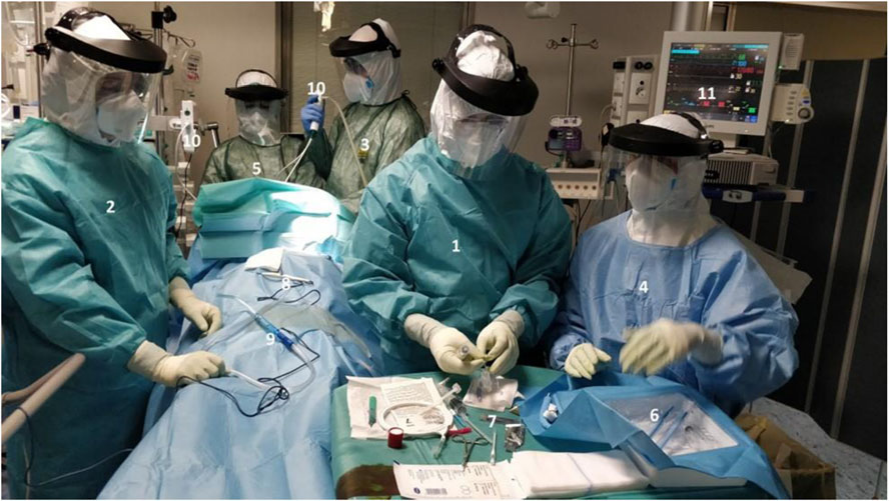
Patient’s bed setting for multidisciplinary team tracheostomy procedure. 1. Intensive care physician (1st operator), 2. ENT Surgeon (2nd operator), 3. ENT Surgeon (airway and fibroscopy management), 4. Instrumental nurse, 5. Anesthesia nurse, 6. Percutaneous tracheostomy set, 7. Part of surgical tracheostomy set, 8. Electrocautery device, 9. Mechanical suctioning unit (Yankauer cannula), 10. single-use flexible bronchoscope, 11. multi-parameter monitor. All subjects in the figure have given their consent for the image to be published

## P518 Mental health of ICU versus non-ICU healthcare workers in a peripheral private hospital during the COVID-19 outbreak in Belgium

### S Noels^1^, H Vanden Eede^2^

#### ^1^Antwerp University Hospital, Anesthesiology, Edegem, Belgium; ^2^AZ Rivierenland, Anesthesiology, Rumst, Belgium

**Introduction:**

The primary aim of this study was to compare the effect of the COVID-19 outbreak on the mental health of ICU and non-ICU healthcare workers. The secondary goal was to look for gender differences in coping with this situation.

**Methods:**

A cross-sectional exploratory design was used. Participants were recruited in the first weeks of the Belgian outbreak. The inclusion criteria were healthcare workers working in high risk areas. Self-administered surveys concerning their education, work environment and personal experience used questions from standardized questionnaires. Correlations (95% level of significance) were searched using logistic regressions.

**Results:**

A total of 73 individuals completed the survey, giving a 75% participation rate. For our primary outcome, we didn’t find a significant difference between ICU and non-ICU nurses. We did find a significant correlation for nurses originating from the OR (Odds = 2.07) and the orthopedic ward (Odds=2.07), and the change of behavior at home. For our secondary goal, we found a clear gender difference in coping with the outbreak. Females showed a greater presence of anxiety (M=0%; F=14%) and felt more worried about the situation. (M=18.75%; F=45.5%). They were more afraid of getting infected (Odds=1.02) and infecting their family (Odds=1.63). Additionally, the female gender had an influence on having behavioral changes at home (Odds=0.66). Furthermore, women tend to find more solace in their family and are more in need of a mental break in comparison to men. Other results are summarized in Table 1.

**Conclusions:**

Our study highlights that well-trained ICU nurses have the same risk for psychological distress than other nurses. Furthermore, female healthcare workers may need more psychological support. Protecting and educating healthcare workers seems to be an important component to address a pandemic outbreak. Finally, it could be interesting to compare this data with new surveys in the following months to evaluate the evolution of their mental health.

**Table 1 (abstract P518).** ICU vs. non-ICU nurses and gender differences in coping with the COVID-19 outbreak

Data (n=73)	Non-ICU healthcare personnel (n=62)	ICU nurses (n=11)	Male (n=16)	Female (n=57)
Current feeling	Worried: n=22 (35.5%) Not worried: n=30 (48.4%)	Worried: n=7 (63.6 %) Not worried: n=1 (9.1%)	Worried: n=3 (18.75%) Afraid: n=0 (0%)	Worried: n=26 (45.5%) Afraid: n= 8 (14%)
Sufficient safety measurements	Yes: n=49 (79%) No: n=9 (14.5%)	Yes: n=9 (81.8%) No: n=2 (18.2%)	Yes: n=10 (62.5%) No: n=4 (25%)	Yes: n=48 (84.2%) p-value <0.0001 No: n=7 (12.3%)
Sufficient support of the hospital management	Yes: n=47 (75.8%) No: n=6 (9.7%)	Yes: n=9 (81.8%) No: n=1 (9.1%)	Yes: n=9 (56.25%) No: n=3 (18.75%)	Yes: n=47 (82.4%) P-value <0.0001 No: n=4 (7%)
Insomnia/stress: behavioral changes at home	Yes: n=41 (66.1%) No: n=20 (32.2%)	Yes: n=8 (72.7%) No: n=3 (27.3%)	Yes: n=12 (75%) No: n=4 (25%)	Yes: n=37 (64.9%) p-value=0.0182 No: n=19 (33.3%)
Fear of getting infected	Themselves: n=40 (64.5%) Family: n=47 (75.8%)	Themselves: n=9 (81.8%) Family: n=10 (90.9%)	Themselves: n=9 (56.25%) Family: n=11 (68.75%)	Themselves: n=40 (70.2%) Family: n=46 (80.7%)
Need for mental break	Yes: n=47 (75.8%) No: n=12 (19.3%)	Yes: n=9 (81.8%) No: n=2 (18.2%)	Yes: n=8 (50%) No: n=6 (37.5%)	Yes: n=48 (84.2%) p-value<0.0001 No: n=8 (14%)
Relaxing activities to cope with situation	Alcohol: n=17 (27.4%) Sport: n=21 (33.9%)	Alcohol: n=2 (18.2%) Sport: n=2 (18.2%)	Alcohol: n=4 (25%) Family: n=1 (6.25%)	Alcohol: n=13 (22.8%) Family: n=13 (22.8%)

## P519 New insights for surge capacity during COVID-19 pandemia: the Eastern Piedmont wayout

### A Costa^1^, M Farah Dell´Aringa^1^, M Costa^1^, M Cavagnino^1^, F Crimaldi^1^, R Vaschetto^1^, F Della Corte^1^, D Colombo^2^

#### ^1^Università del Piemonte Orientale, Translational Medicine Department, Novara, Italy; ^2^SS. Trinità Hospital - ASL Novara, Translational Medicine Department, Borgomanero, Italy

**Introduction:**

Hub and spoke network is set in order to provide the best care for specialistic pathologies. New or unknown ills, therefore, may be preferentially centralized to hub hospital. Surge capacity, usually based on pre-disaster situation and different algorithms (despite some variations), follow the criterion “the more you have (Staff, Stuff, Space) the more you have to surge”. Following this principles, COVID-19 pandemic have the potential to overwhelm hub hospitals, forcing a big reduction of specialistic activities for a long period with the risk of reducing the treatment capacity of many other life-threatening pathologies. We would like to present the activity of a network of hospitals in Northern Italy, and how they organized their ICU network to cope with the crisis.

**Methods:**

Eastern Piedmont is a region with 6 hospital, one hub university hospital and 5 spoke hospitals. The hub hospital is the only facility with advanced specialties. In order to keep specialistic services available, spoke hospitals proportionally increased their capacity more than the hub hospital by cutting their overall activity.

**Results:**

Spoke hospital ICU beds increment was 284% (min 225% max 417%) compared to a 155% increase of the hub hospital (Figure 1). Total bed capacity went from 51 to 115 beds. The hub hospital managed to keep 7 specialistic elective surgical room open every day during the crisis, while 4/5 spoke hospitals held 1 each.

**Conclusions:**

An increment of 200% in capacity is usually considered the goal of crisis surge response but this could be achieved for brief period of times without affecting the quality of care. Considering the duration of the epidemic and the need to keep specialistic services available for all the patients, it is fundamental to adapt the current surge capacity model to take into account other hospitals in the network to deliver the best possible quality of care. We believe it is possible to use this organizational model to better estimate surge capacity during pandemics in similar hospital networks.

**Fig. 1 (abstract P519).**
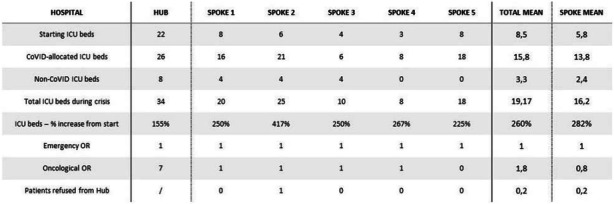
ICU beds and OR capabilities available in Eastern Piedmont before and during the COVID-19 crisis

## P520 Survey of current practice in management of anticoagulation in adult critically ill patients with COVID-19 in the Northwest of England

### SR Smith^1^, A Waite^2^, BW Johnston^3^, ID Welters^3^

#### ^1^University of Liverpool School of Medicine, Liverpool, United Kingdom; ^2^Royal Liverpool University Hospital, Intensive Care, Liverpool, United Kingdom; ^3^Royal Liverpool University Hospital, Liverpool, United Kingdom

**Introduction:**

There is increasing evidence that coronavirus disease 2019 (COVID-19) causes micro- and macrovascular thrombi. However, the best prophylactic anticoagulation strategy for critically ill patients with COVID-19 remains unclear with no concordant official guidance [1]. The purpose of this survey is to establish which anticoagulation strategies are being used for critically ill patients with COVID-19 within the Northwest of England.

**Methods:**

We conducted a 13-item online survey assessing local anticoagulation strategies and thromboembolic events in adult, critically ill patients with COVID-19. Clinical directors of critical care units within Northwest England were invited to participate and share the questionnaire with their consultant and junior doctor colleagues. The survey was conducted between April 17th and May 1st 2020.

**Results:**

There were 38 respondents, of which 66% were consultants and 29% were junior doctors. The majority of participants (91%) reported regularly using prophylactic anticoagulation. Half of respondents (49%) reported that they had changed their anticoagulation strategy to split dose low molecular weight heparin (LMWH), whilst 31% had changed to therapeutic dose LMWH. D-dimer was used to guide anticoagulation by 15% of respondents, with cut offs of 1000 and 3000 ng/ml as an indicator to give higher dose LMWH. Anti-Xa levels were used by 17% of participants to guide the dose of prophylactic anticoagulation.

**Conclusions:**

Prophylactic anticoagulation is still routinely used to treat critically ill patients with COVID-19 in the Northwest of England, but there is variation in practice, and deviation from usual practice due to clinical evidence of thromboses. There is an urgent need for data to guide the anticoagulation strategy in patients with COVID-19.

**Reference**

1. Thachil J et al. J Thromb Haemost 18:1023‐1026, 2020.

## P521 Non-invasive brain compliance findings in patients with COVID-19 in a Brazilian intensive care unit

### SS Rojas^1^, VC Veiga^1^, J Carvalho^2^, AC Lisboa^3^, AA Ordinola^3^, CP Nogueira^3^, MM Silveira^3^, MA Pitaci^3^, S Halla^3^, VM Queiroz^3^

#### ^1^Hospital Beneficência Portuguesa de São Paulo, Intensive Care Unit Coordinator, São Paulo, Brazil; ^2^Hospital Beneficência Portuguesa de São Paulo, Neurocritical Care Unit, São Paulo, Brazil; ^3^Hospital Beneficência Portuguesa de São Paulo, Intensive Care Unit, São Paulo, Brazil

**Introduction:**

Coronavirus disease 2019 (COVID-19) has been spreading worldwide. Research conducted in different countries indicated this virus causes neurotropism and neurological symptoms development. The aim of this study is to analyze brain compliance (BC) of patients diagnosed with COVID-19 at intensive care unit (ICU) admission using non-invasive technique.

**Methods:**

Observational exploratory study conducted at the ICU of Hospital Beneficência Portuguesa in São Paulo, Brazil, including 14 patients with >18 years old with severe acute respiratory syndrome symptoms and positive COVID-19 confirmed by RT-PCR between 23 March and 4 April, a period in which the patients were included in the study. BC was monitored upon the admission through evaluation of changes in intracranial pressure (ICP) pulse, applying a non-invasive sensor (brain4care method, Brain4care© Corp., Brazil) based on the relationship between the amplitudes of the P1 and P2 peaks.

**Results:**

Morbidities were present in 50% and most patients were men with mean age of 57.9±12.1 years old. Glasgow Coma Score, SAPS 3 and SOFA scales showed normal values in admission; however 13 of 14 patients presented changes in ICP waveforms, showing impaired brain compliance. Four patients appear to have the distribution of P2/P1 ratio above 1, while for others, the distributions are very close to 1. ICP pulses were used to calculate the P2/P1 ratio with a mean of 1.08, standard deviation of 0.24 and median of 1.02 in a total 258 observations, with approximately 20 observations per patient (Figure 1). At the 0.05 significance level, the true mean value of P2/P1 is between 1.009 and 1.159. One P2/P1 ratio differs from the others and represents a patient with rheumatoid arthritis, on treatment with corticoids and hydroxychloroquine.

**Conclusions:**

Respiratory distress may produce BC impairment even in early development of this disease. This equipment may represent a tool for the early detection of neurological changes in these patients and help in decision-making.

**Fig. 1 (abstract P521).**
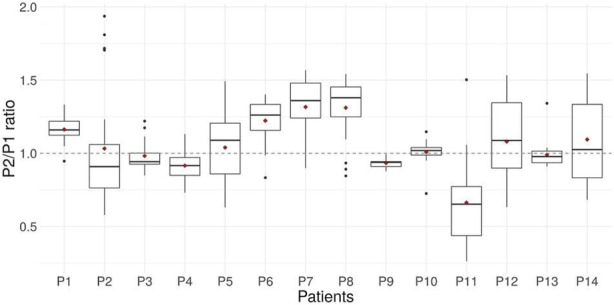
Individualized boxplot analysis of all 14 patients on admission to the ICU

## P522 COVID-19 is associated with a high rate of thrombotic circuit complications during vv-ECMO therapy

### XB Bemtgen^1^, VZ Zotzmann^1^, KS Steiner^1^, AA Asmussen^1^, SB Benk^2^ , CB Bode^1^, TW Wengenmayer^1^, SM Maier^2^, DS Staudacher^1^

#### ^1^Heart Center Freiburg University, Department of Cardiology and Angiology I, Freiburg im Breisgau, Germany; ^2^Heart Center Freiburg University, Department of Cardiovascular Surgery, Freiburg im Breisgau, Germany

**Introduction:**

The novel coronavirus SARS-CoV-2 and the resulting disease COVID-19 is known to have mild as well as critical courses requiring vv-ECMO therapy. Also, COVID-19 seems to be associated with hypercoagulability and thrombosis [1]. Indeed, our center as well did witness an increase of thrombotic complications especially in the extracorporeal circuit in these patients, particularly of the centrifugal pump. Thus, we investigated the rate of vv-ECMO complications in these patients.

**Methods:**

All COVID-19 cases admitted on our ICU who received vv-ECMO therapy were compared with vv-ECMO patients treated on our ICU during the years 2018 and 2019. Aside from baseline characteristics, duration of ICU stay and length of vv-ECMO therapy, all circuit related complications resulting in partial or complete exchange of the extracorporeal system were registered. Events clearly documented as non-thrombotic were excluded. Also D-Dimer measurements prior to these events were analyzed.

**Results:**

In total, 66 patients were analyzed, 55 non-COVID-19 vs. eleven COVID-19-related. To this date, six COVID-19 patients were still treated on our ICU. The two groups did not differ in age, BMI and severity of illness (RESP Score mean 0.89 vs 1.09, p 0.85). A significantly higher rate and probability of centrifugal pump thrombosis needing exchange could be observed in the COVID-19-group (see figure 1). In total, 16 centrifugal pump thromboses did occur in the non-COVID-19-group compared to nine in the COVID-19-group. In addition, the most recent D-Dimer measurements prior to the events were significantly lower in the non-COVID-19 group (mean 15.48 vs 26.59, p < 0.05).

**Conclusions:**

The coronavirus SARS-CoV-2 induced infection is associated with higher rates of thrombotic events of the extracorporeal system during vv-ECMO therapy and the medical team should be watchful to counteract these complications.

**Reference**

1. Leisman DE et al. Intensive Care Med 46:1105-1108, 2020

**Fig. 1 (abstract P522).**
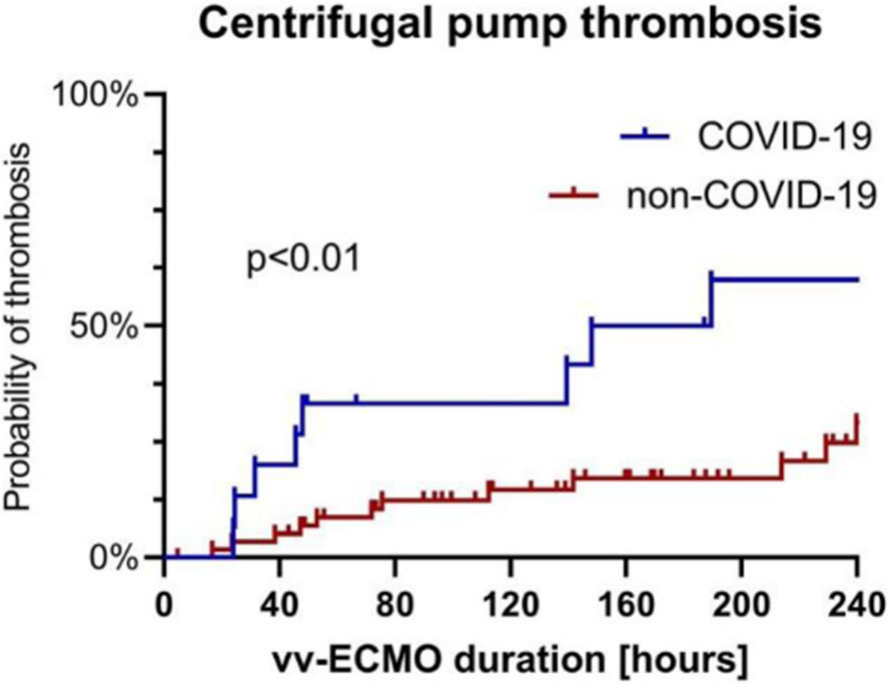
Centrifugal pump thrombosis

## P523 High incidence of barotrauma in patients with COVID-19 pneumonia during invasive mechanical ventilation

### J Udi^1^, V Zotzmann^1^, CN Lang^1^, A Fluegler^1^, K Krueger^1^, D Duerschmied^1^, F Bamberg^2^, C Bode^1^, T Wengenmayer^1^, DL Staudacher^1^

#### ^1^Department of Cardiology and Angiology I (Heart Center) and Department of Internal Medicine III (Intensive Care Medicine), University of Freiburg, Faculty of Medicine, Freiburg, Germany; ^2^Department of Radiology, University of Freiburg, Faculty of Medicine, Freiburg, Germany

**Introduction:**

COVID-19 can cause pulmonary failure and even acute respiratory distress syndrome (ARDS) requiring prolonged mechanical ventilation (MV). It is known that MV by itself comes with complications like superinfections and barotrauma. Since it has been proposed by Gattinoni et al that COVID-19 pneumonia may have two phenotypes [1], an early one presenting with low elastance and recruitability and the later one with features of ARDS, we evaluated all COVID-19 patients on MV for barotrauma.

**Methods:**

All patients with COVID-19 pneumonia on MV treated at our intensive care unit (university hospital, ARDS and ECMO reference center) between March and April 2020 were included. Characteristics of MV during the last 24 hours (h) before any complication were recorded. This retrospective registry is covered by an ethics approval (file 234-20).

**Results:**

A total of 20 patients with COVID-19 pneumonia were included (median age: 61 years, 6 female, duration of MV 22 days, 55% on venovenous extracorporeal membrane oxygenation (vv-ECMO). Of these, 8 patients (median age: 62 years, 3 female, 4 on vv-ECMO) developed barotrauma (40%) including pneumothorax (n=5), pneumomediastinum (n=5) and subcutaneous emphysema (n=2) under MV (Figure 1). Only 1 patient had a predisposing lung disease (chronic obstructive lung disease). Median MV duration before complication occurs was 18 days (range: 1-32). Median MV parameters from all 8 patients during the last 24 h before barotrauma, were: inspiratory oxygen fraction (FiO2) 55% (range: 45-70) peak inspiratory pressure 27 mbar (range: 20-29), positive end-expiratory pressure (PEEP) 12 mbar (range: 5-16), tidal volume (VT) 453 ml (range: 41-775), and respiratory frequency (RF) 22/min (range: 15-30), 63% spontaneous breathing, 50% prone positioning.

**Conclusions:**

In our experience, barotrauma is a frequent complication in the late phase of COVID-19 induced ARDS. Preliminary data suggest that barotrauma in COVID-19 may occur even when following recommendations for lung protective MV in ARDS.

**Reference**

1. Gattinoni L et al. Intensive Care Med 46:1099–1102, 2020

**Fig. 1 (abstract P523).**
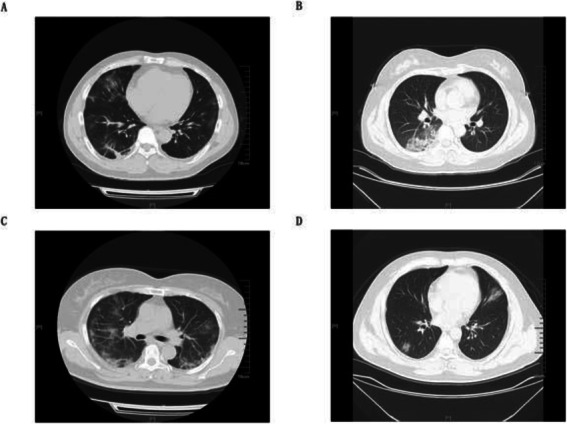
Full-body CT scan showing an extended subcutaneous emphysema, pneumomediastinum and pneumopericardium

## P524 Pulmonary embolism is a frequent complication in COVID-19 patients on the intensive care unit

### V Zotzmann^1^ , CN Lang^1^, N Gauchel^1^, X Bemtgen^1^, F Bamberg^2^, C Bode^1^, T Wengenmayer^1^, D Duerschmied^1^, DL Staudacher^3^

#### ^1^Heart Center Freiburg University, Freiburg im Breisgau, Germany; ^2^University of Freiburg, Department of Diagnostic and Interventional Radiology, Freiburg im Breisgau, Germany; ^3^Heart Center Freiburg University, Department of Cardiology and Angiology I, Freiburg im Breisgau, Germany

**Introduction:**

COVID-19, the disease caused by SARS-CoV-2 became a major health issue in 2020. One of the most recognized laboratory changes associated with COVID-19 is an increase in d-dimer levels, which correlates with poor prognosis. The origin of the elevated D-dimer levels however is poorly understood. One of the distinct characteristics of COVID-19 however is a prothrombotic state. Here, we investigate if pulmonary embolism (PE) might be more frequent in this patient collective.

**Methods:**

All COVID-19 patients admitted to our medical intensive care unit are screened as part of the daily routine for clinical signs for PE including bed-side ultrasound, laboratory parameters and the Wells-score. In case of high clinical probability of PE, a computed tomography pulmonary angiogram (CTPA) was performed. Data was retrospectively collected and analyzed.

**Results:**

Twenty-two patients (age 60.4±10.2 years, 27.3% female, 91% BMI >25kg/m²) were investigated. All patients were either on systematic prophylactic anticoagulation (16/22) or on systematic therapeutic anticoagulation (6/22) which was initiated for a preexisting indication for oral anticoagulation. Within the investigated time period, 16 out of the 22 patients (73%) were considered to have a high likelihood of PE and underwent at least one CTPA. PE was detected in 8 out of 16 patients (50%). According this cohort, the PE rate in COVID-19 patients on the ICU might be between 36.4% (8/22, in the entire cohort) and 50.0% (8/16, in patients undergoing CTPA). At time of CTPA, there was a significant difference in the level of D-dimers in the group in which pulmonary embolism compared to those without (26.5±11.2 and 7.8±10.3 mg/l, respectively, p<0.01). There was no difference in respect to first recorded or highest D-dimer levels comparing patients with and without PE.

**Conclusions:**

Despite systematic prophylactic anticoagulation, PE occurred in more than 36% of COVID-19 patients in our ICU. PE might be a disease-specific complication of COVID-19.

## P525 Incidence of post-intensive care syndrome in conscious survivors from medical critical illness in a university hospital

### N Kongpolprom^1^, P Srisawart^2^, Y Pyharnvichitnooch^3^

#### ^1^King Chulalongkorn Memorial Hospital, Pulmonary Unit, Bangkok, Thailand; ^2^King Chulalongkorn Memorial Hospital, Department of Psychiatry, Faculty of Psychiatry, Chulalongkorn University, Bangkok, Thailand; ^3^King Chulalongkorn Memorial Hospital, Department of Medicine, Faculty of Medicine, Chulalongkorn University, Bangkok, Thailand

**Introduction:**

Post-ICU syndrome (PICS) is defined as a new or worsening impairment in physical, cognitive, or mental health status arising and persisting after hospitalization for critical illness. The incidence of PICS varies among different countries. However, the incidence and risk factors of PICs in Thais remained unknown.

**Methods:**

We conducted a prospective observational study to determine the incidence of PICS among medical ICU survivors during 28 days after ICU discharge. We evaluated PICS parameters, including functional disability, cognitive impairment, psychological impairment, namely anxiety, depression, and sleep quality. In addition, we analyzed risk factors for PICS and the association between PICS and 90-day mortality.

**Results:**

A total of 68 conscious ICU survivors were analyzed in our study. The majority of them were male (55%) with a mean age of 56.6 ±20.8 years and a mean APACHE II of 16 ± 4.6. The incidence of PICS during the 28-day period was 64.71%. Poor sleep quality was the most common presentation of PICS (59%). Approximately 50%, 27%, and 16% of patients with PICS presented with functional disability, cognitive impairment, and anxiety or depression, respectively. Delirium, hypo/hyperglycemia, and hypoxemia during ICU admission were risk factors for PICS with relative risks of 1.59 (95%CI 1.21 to 2.09), 2.70 (95% 1.44 to 5.09) and 2.02 (95% CI 1.32 to 3.07), respectively. Nevertheless, vasopressor requirement, mechanical ventilation, noninvasive ventilation, intravenous sedation, and sepsis were not associated with PICS. There was no significant association between PICS and 90-day mortality.

**Conclusions:**

Incidence of PICS among Thai ICU survivors was relatively high, compared to previously published data of other countries. Risk factors for PICS included delirium, hypo/hyperglycemia, and hypoxemia. Preventive strategies during ICU admission to minimize such risk factors might decrease PICS and improve long-term ICU outcomes.

## P526 Why are acute stroke patients not receiving i.v. tPA in Republic of Moldova

### G Ciobanu, A Oglinda

#### State University of Medicine and Pharmacy “Nicolae Testemitanu”, Emergency Medecine, Chisinau, Moldova

**Introduction:**

Stroke is one of the leading causes of morbidity and mortality, accounting for 11.13% of total deaths worldwide. The development of hyper-acute treatments such as thrombolysis, organization of stroke units has changed considerably the field of stroke medicine. In Republic of Moldova in 2019 were registered 5937 new cases of stroke, including 4810 cases (81%) of ischemic, 1009 cases (17%) of hemorrhagic stroke and 118 cases of subarachnoid hemorrhage (SAH). Prevalence of cerebrovascular diseases is 274.8, incidence is 30.3 per 10 000 population.

**Methods:**

A retrospective hospital-based study was conducted at EMI in Chișinău, Republic of Moldova. All medical records with a diagnosis of stroke were identified based on the ICD, R10, from January 01, 2019 to December 31, 2019. The aim of this study was to identify reasons acute stroke patients did not receive thrombolysis.

**Results:**

Out of 456 patients 248 (54.4%) were male and 208(45.6%) were female, the mean age of the patients was 60.4±17.4 years. 79.4% of acute ischemic stroke patients did not receive thrombolytic therapy (n=362). The incidence of different risk factors in ischemic stroke were as follows: 42.6% hypertension,32.7% smoking, 32.2% alcohol intake, 24.8% diabetes, 22.6% coronary artery disease, 18.6% dyslipidemia, 16.6 % dysrhythmia, 13.4% previous stroke, 10% inactivity, 8.8% transient ischemic stroke in the past. The most common reason who did not receive thrombolytic therapy were: exceedance of time window 34.2%; low or improved NIHSS 19.8%; uncontrolled blood pressure 6.8%; stroke mimics 5.6%; history of stroke in the past 3 months 4.2%; history of gastrointestinal bleeding 2.8%; family refusal 1.4% and no alteplase availability 2.0%.

**Conclusions:**

In the Republic of Moldova, only 20.6% of ischemic stroke patients receive thrombolytic. The most common reason who did not receive thrombolytic therapy were: exceedance of time window 34.2%, low or improved NIHSS 19.8%, uncontrolled blood pressure 6.8%, stroke mimics 5.6% and history of stroke in the past 3 months 4.2%.

## P527 Mitochondrial and microbial metabolites in cerebrospinal fluid

### N Beloborodova^1^, A Pautova^2^, N Burnakova^3^

#### ^1^Federal Research and Clinical Center of Intensive Care Medicine and Rehabilitology, Lab. Metabolisms in Critical State, Moscow, Russian Federation; ^2^Federal Research and Clinical Center of Intensive Care Medicine and Rehabilitology, Negovsky Research Institute of General Reanimatology, Moscow, Russian Federation; ^3^Lomonosov Moscow State University, Chemistry Department, Moscow, Russian Federation

**Introduction:**

In a previous study the increase in levels of some microbial phenolic metabolites (phenyllactic (PhLA), p-hydroxyphenylacetic (p-HPhAA), p-hydroxyphenyllactic (p-HPhAA) acids) simultaneously with a rise in levels of mitochondrial dicarboxylic (succinic and fumaric) acids were detected in serum samples of septic patients. A close direct correlation of serum concentrations of these metabolites confirms the participation of the phenolic metabolites in the development of mitochondrial dysfunction in sepsis [1]. The purpose of this study was to find out whether there are similar correlations in the cerebrospinal fluid (CSF).

**Methods:**

Concentrations of phenolic (PhLA, p-HPhLA, p-HPhAA, p-hydroxybenzoic (p-HBA), homovanillic (HVA)) and dicarboxylic (succinic, fumaric) acids were measured by gas chromatography–mass spectrometry. We used a collection of frozen CSF samples (n = 101) of critically ill neurosurgical patients with various intracranial diseases or injuries.

**Results:**

Phenolic acids were found in CSF samples at low concentrations 0.1-1.1 μM. Dicarboxylic acids were detected in different concentration ranges: succinic acid 0.6-111 μM, fumaric acid 0.1- 5.3 μM. Close direct correlations (r) of microbial and mitochondrial metabolites were revealed (Table 1). Correlation between fumaric and succinic acid was 0.78. These correlations are similar to those obtained previously in the sera of septic patients [1].

**Conclusions:**

Correlations of phenolic and mitochondrial metabolites in CSF are similar to those previously found in the blood of critically ill septic patients. The new data relate to the revealed correlation between the metabolite of microbiota p-HBA and mitochondrial metabolites which needs to be explained in further research.

Acknowledgements

Supported by the grant of President of the Russian Federation [No. MK-627.2020.7]

**Reference**

1. Beloborodova N et al. Metabolites 9:196-213, 2019.

**Table 1 (abstract P527).** Correlations (r) between the content of microbial phenolic acids and mitochondrial dicarboxylic acids in CSF samples (n = 101) of neurosurgical patients, p < 0.05, ns – not significant

Metabolite	p-HBA	p-HPhAA	HVA	p-HPhLA
Fumaric acid	0.42	0.45	0.63	0.39
Succinic acid	0.34	0.36	0.66	0.25
PhLA	0.45	0.61	ns	0.66
p-HPhLA	0.53	0.75	0.43	-
HVA	0.40	0.41	-	0.43
p-HPhAA	0.50	-	0.41	0.75

## P528 Are we using NICE guidelines appropriately for CT head of head injury patients?

### B Shurovi^1^, GP Prajapati^2^, NN Nayeem^2^

#### ^1^University Hospital Lewisham, Critical Care, London, United Kingdom; ^2^University Hospital Lewisham, Emergency Medicine, London, United Kingdom

**Introduction:**

Head injury is the commonest cause of death and disability in people aged 1-40 years in the United Kingdom. This study evaluated whether we were appropriately using NICE Guidelines [1] to assess head injury patients by performing CT head.

**Methods:**

A single center retrospective study was carried out looking at patients attending the Emergency Department in August 2019 with suspected head injury. 102 patients were identified using iCare and data collection was completed using FirstNet and PACs

**Results:**

Total of 102 patients fit the criteria out of which 40 patients had CT head. 20 out of 40 patients fit the criteria for CT head as per NICE guidelines. Mean time to be triaged in ED was approximately 25 minutes. Mean time to be seen after triage was approximately 2 hours. CT head performed within 1 hour of request was 36%. CT head performed within 1 hour of triage was 7%. CT head reported within 1 hour was 75%.

**Conclusions:**

This retrospective study showed that doctors and nurses are not compliant with the NICE guidelines as 50% of CT head were not warranted. CT head was not being performed within the 1st hour of head injury presentation: delay of approximately 2-hour 20minutes between triage and assessment by a medical practitioner. 75% of data suggest CT head was being reported within 1 hour, which is compliant with NICE guidelines. Moreover, it was impossible to say whether some of the risk factors such as vomiting, seizures or LOC was not assessed or merely not documented. This data suggested that an intervention is required to improve the documentation process and compliance of doctors and nurses in order to improve the delays between triage, assessment and performing CT head. Furthermore, the cost of unwarranted CT scans and patients being exposed to unnecessary radiation could be reduced. Hence, we are implementing two quality improvements projects in order to address the above issues.

**Reference**

1. National Institute for Health and Care Excellence (2017) Head injury: assessment and early management. NICE guideline

## P529 Risk factors of secondary neurologic deterioration after moderate traumatic brain injury: a retrospective cohort

### P Aries^1^, A Cadieu^1^, J Ognard^2^, O Huet^1^

#### ^1^CHRU Brest, Department of Anaesthesia and Surgical Intensive Care, Brest, France; ^2^CHRU Brest, Department of Radiology, Brest, France

**Introduction:**

Patients with moderate traumatic brain injury (mTBI) are 1.5 times more frequent than those with severe TBI and some of them will develop secondary neurologic deterioration (SND) within the first 7 days [1, 2]. However, identifying at risk patients of SND is still challenging. This study aimed to determine risk factors associated with SND after mTBI.

**Methods:**

We conducted a single center retrospective study. Adults admitted in Brest hospital between 2015 and 2018 for mTBI, defined by Glasgow coma scale (GCS) score 9-13, were eligible. We assessed clinical patients’ characteristics in the prehospital setting, at admission and during the first 72 hours of hospitalization. Biology, transcranial Doppler (TCD) and CT examinations were also reported. SND was defined either by a decrease in GCS or by a deterioration in neurologic status sufficient to warrant intervention like mechanical ventilation, transfer to the ICU or neurosurgery [3]. Factors statistically associated with SND were identified.

**Results:**

147 patients were included, mean patient age was 51.5 years (± 18.94 years) and 81% of patients were men. Mean GCS score was 11.4 (± 1.63). 46 (31.3%) showed SND and 14-day mortality rate was of 18.4%. Patients with SND were older (p<0.001), had higher hypoxemia and intubation rates (p=0.011, p=0.002 respectively) before admission. They had significantly higher IGS2 scores on admission (p <0.001), and more frequent hyperglycemia (>8 mmol/l) and hypotension (SBP < 90 mmHg) (p=0.001 and p<0.001 respectively) during the first 72 hours. Neuro-worsening was also associated with abnormal TCD (pulsatility index > 1.4) and abnormal head CT scan on admission (higher categories at Marshall CT score) (p=0.004 and p<0.001 respectively). 32% of patients with SND required neurosurgery.

**Conclusions:**

We report for the first time the largest study about early outcome after mTBI. About a third of mTBI patients showed SND and early factors could be used for determining at risk patients.

**References**

1. Tagliaferri F et al. Acta Neurochir (Wien) 148:255–68, 2006

2. Davis DP et al. J Trauma 62:277–81, 2007

3. Morris GF et al. Neurosurgery 43:1369–72, 1998

## P530 Serum lactate level fails to predict clinical outcomes in patients undergoing surgical resection of brain tumors: a cross-sectional study

### G Madrid^1^, D Cohen^1^, L Moreno^2^, A Ordoñez^2^, M Solórzano^1^, MC Niño^1^

#### ^1^Fundación Santa Fe de Bogotá, Anesthesiology, Bogotá, Colombia; ^2^Fundación Santa Fe de Bogotá, Critical Medicine and Intensive Care , Bogotá, Colombia

**Introduction:**

Primary tumors of the CNS account for 1-2% of all malignant tumors and are responsible for approximately 2-3% of cancer-related deaths. For these patients, treatment includes surgical resection and postoperative management in the ICU where multimodal monitoring, goal-directed therapy and analysis of serum markers are essential. Serum lactate is a commonly used marker of global tissue perfusion, so it correlates to prognosis and clinical outcomes of patients. Thus, blood lactate has been used widely in critically ill patients. This study aims to assess the behavior of serum lactate as an outcome predictor in patients undergoing surgical resection of brain tumors.

**Methods:**

After Institutional Review Board approval was obtained, a cross-sectional study was conducted. All patients older than 18 years undergoing brain tumor resection between January 2015 and December 2019 were included. We excluded septic patients and those with hyperlactatemia. For analysis patients were divided into two groups according to their serum lactate levels: <2 mmol/l (normal) and ≥2 mmol/l (hyperlactatemia). Measured clinical outcomes included duration of mechanical ventilation, reintubation, length of stay in ICU, length of hospital stay, readmission to the ICU and mortality. P<0.05 was considered statistically significant.

**Results:**

A total of 225 patients were analyzed, of which 154 patients (64.4%) had a normal level of serum lactate and 80 patients (35.6%) presented hyperlactatemia. There were no statistically significant differences in duration of mechanical ventilation (p=0.07), reintubation (p=0.06), length of stay in ICU (p >0.05), length of hospital stay (p>0.05), and readmission to the ICU (p=0.09). Mortality was higher in patients with hyperlactatemia (p=0.04).

**Conclusions:**

Serum lactate is not a good predictor of postoperative clinical outcomes in patients undergoing brain tumor resection. However, it may predict mortality in these patients, so it is necessary to conduct another study with larger sample size.

## P531 Targeted temperature management and neurological outcome after out of hospital cardiac arrest

### J Ellis, C Pritchett, J Thorns, BA Alberts, R Tedstone, M Spivey

#### Royal Cornwall Hospital, Intensive Care, Truro, United Kingdom

**Introduction:**

Our 2017 audit of targeted temperature management (TTM) in patients admitted to ICU after an out of hospital cardiac arrest (OOHCA) found that patient temperatures were above those recommended by the Resuscitation Council (RC). Previous data showed that cooling was best achieved in 2013; the year with the highest use of invasive cooling. Following the audit, additional education was given to ICU staff on TTM. We repeated this audit to assess the effect of that intervention.

**Methods:**

Patients admitted following OOHCA between 01/04/2017 and 31/06/2019 were identified, excluding patients aged under 18. Baseline data included gender, age, total ‘downtime’, presenting rhythm, and survival to discharge. Data was also collected on cooling method, whether TTM was documented in the medical plan, and temperature at 0, 12, 24, 48 and 72 hours after ICU admission. Cerebral performance category (CPC) was gaged using clinic letters.

**Results:**

96 patients were admitted to ICU following OOHCA between 01/04/2017 and 31/06/2019. Of these, 71.9% were male, the mean age was 63, and 38.5% of patients admitted survived to hospital discharge. TTM was documented in the medical plan for 58.3% of patients. The percentage of patients receiving invasive cooling in 2017-19 was 22.6%, 25.0% and 29.4% respectively. Across all years, the mean temperatures at 0, 12, 24, 48 and 72 hours following ICU admission were 34.9°C, 36.4°C, 36.5°C, 36.6°C and 36.9°C. Comparison between patients with a CPC of 1 and CPC of 5 showed no significant difference in temperature during ICU stay.

**Conclusions:**

The results of this audit are similar to those obtained in 2017, implying additional education has not resulted in better adherence to RC guidelines of TTM below 36°C for 24 hours. However, average temperatures in the first 72 hours are below the recommended 37.6°C. In the future, it would be interesting to compare incidences of pyrexia in those cooled passively compared to those given invasive cooling and to assess for differences in outcomes.

## P532 Outcomes from out-of-hospital cardiac arrests in Kaunas (Lithuania) in 2017-2018

### L Darginavicius^1^, I Kajokaite^2^, D Ieskiene^2^, N Mikelionis^3^, J Vencloviene^2^, G Beinaraviciute^1^, T Taluntis^1^, E Vaitkaitiene^1^, D Vaitkaitis^1^, A Krikscionaitiene^4^

#### ^1^Lithuanian University of Health Sciences, Kaunas, Lithuania; ^2^Vytautas Magnus University, Kaunas, Lithuania; ^3^Kaunas Emergency Medical Service Station, Kaunas, Lithuania; ^4^Lithuanian University of Health Sciences, Department of Emergency and Disaster Medicine, Kaunas, Lithuania

**Introduction:**

EuReCa TWO study [1] has recently reported large variation in out-of-hospital cardiac arrest (OHCA) outcomes across the Europe. Unfortunately, Lithuania did not participate in that study. We sought to fill the gap and describe the epidemiology and outcomes from Kaunas, the second largest Lithuanian city with a population of 0.29 million (9.7% of all population in Lithuania).

**Methods:**

The incidence, demographics and outcomes of patients who were treated for an OHCA between 1st January 2017 and 31st December 2018 in Kaunas Emergency medical Service (EMS) were collected and are reported in accordance with 2014 Utstein recommendations.

**Results:**

In total, 524 OHCA cases of EMS treated cardiac arrests were analyzed. The mean age was 68.6 (SD = 16) years and 66.3% were male. 74% OHCA cases occurred at home and 63.2% were witnessed. The initial rhythm was shockable in 28.6% and non-shockable in 66.6% of all cases. Return of spontaneous circulation (ROSC) at hospital transfer was 28.2% in 2017, and 31.8% in 2018. Survival to hospital discharge was 12.1% in 2017, and 12.7% in 2018. Survival to hospital discharge in Utstein comparator group (OHCA witnessed by a bystander, and shockable rhythm) was 31.8% in 2017, and 44.2% in 2018.

**Conclusions:**

ROSC and survival to hospital discharge in Kaunas were slightly better to those reported as median in EuReCa TWO study.

**Reference**

1. Grasner JT et al. Resuscitation 148:218-26, 2020

## P533 Nursing feedback to introducing ICU doulas to provide psychological support for the critically ill

### KR Johnson^1^, KL Philbrick^2^, K Varga^3^, LV Karnatovskaia^1^

#### ^1^Mayo Clinic, Pulmonary and Critical Care Medicine, Rochester, MN, United States; ^2^Mayo Clinic, Pyschology, Rochester, MN, United States; ^3^ELTE Eötvös Loránd University, Institute of Psychology, Budapest, Hungary

**Introduction:**

Over a third of critical illness survivors suffer from mental health problems following hospital discharge. Memories of delusional experiences are a major risk factor. Research on the formation of fear demonstrates that if mitigating information about a traumatic event is introduced during the time of memory formation/upon its subsequent recall, the emotional experience of the memory may be modified. Given that semantic processing continues during altered states of consciousness, we trained doulas to provide early psychological support for the critically ill in parallel with medical treatment. Stakeholder acceptance is a vital part of the intervention success.

**Methods:**

ICU nurses who witnessed the interventional session were given a paper questionnaire with multiple choice and open-ended items regarding their impressions.

**Results:**

43 patients received the intervention; 32 nurses provided feedback. When asked about what they liked, all provided positive comments about the intervention (see Figure 1). When questioned about what they didn’t like, only 11 (34%) commented including that positivity may not always reflect reality, that it may overstep the nurse’s job, that the intervention should be longer, or recommending better coordination with nurses/shift changes. When asked whether communicating with the ICU doulas was also helpful for the nurse, 26 (81%) answered yes. Specifically, nurses liked: aspects of the intervention that provided patients with reorientation and reassurance; ICU doulas being a liaison with the family and team and answering questions; and, being able to step away to complete other tasks while someone was there with the patient. Of the 6 respondents who did not find the intervention helpful to nurses, only two elaborated that the intervention appeared redundant to the care nurses already provide.

**Conclusions:**

The majority of bedside ICU nurses welcome the presence of ICU doulas and the psychological support intervention they provide.

**Fig. 1 (abstract P533).**
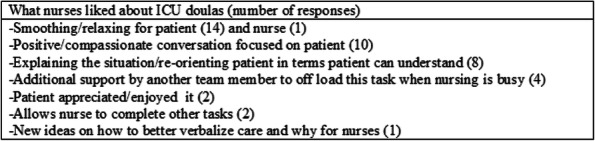
ICU nurse questionnaire responses regarding ICU doulas

## P534 Patients presenting with ST-elevation myocardial infarction and high thrombus burden: the role of mechanical thrombectomy in combination with deferred stenting

### K Kintis, E Papadakis , D Antonatos, A Poulianitou, V Kiriakopoulos, S Patsilinakos

#### Konstantopoulio General Hospital, Cardiology, Athens, Greece

**Introduction:**

High thrombus burden is an independent risk factor for death and complications, including no reflow, during primary percutaneous coronary intervention (PCI) for STEMI. The aim was to investigate whether a strategy of mechanical thrombectomy in combination with deferred stenting is associated with a reduced incidence of slow- or no-reflow, and other thrombotic complications compared with stenting in patients with high thrombus burden.

**Methods:**

A total of 210 patients with STEMI and high thrombus burden treated with thrombus aspiration in combination with glycoprotein IIb/IIIa inhibitors with or without stent implantation. Patients were divided into 2 groups: non-stent PCI group (n = 105) and stent PCI group (n = 105). The end points were a myocardial blush grade of 0 or 1 (defined as absent or minimal myocardial reperfusion, respectively) and the postprocedural frequencies of a TIMI flow grade of 3, 48 hours after primary PCI, complete resolution of ST-segment elevation immediately after primary PCI, target vessel revascularization, reinfarction, death, and the combination of major adverse cardiac events by 30 days after randomization.

**Results:**

A myocardial blush grade of 0 or 1 occurred in 26.3% of the patients in the stent PCI group and in 17.1% of those in the non-stent PCI group (p < 0.05). Complete resolution of ST-segment elevation occurred in 86.6% and 78.2% of patients, respectively (p = 0.35). At 30 days, the rate of death in the stent PCI group and non-stent PCI group was 1.7%, and 1.0%, respectively (p = 0.33), and the rate of adverse events was 12.1% and 2.2%, respectively (p < 0.01).

**Conclusions:**

Mechanical thrombectomy in combination with glycoprotein IIb/IIIa inhibitors without stenting is applicable and effective method in a large majority of patients with myocardial infarction with ST-segment elevation and high thrombus burden. It results in better reperfusion outcomes than conventional PCI with stent, irrespective of clinical and angiographic characteristics at baseline.

## P535 Preload functional status and cardiac output in sepsis

### J Sahatjian^1^, I Douglas^2^, M Exline^3^, L Forni^4^, D Kaufman^5^, A Khan^6^, G Martin^7^, J Weingarten^8^, M Williams^9^, D Hansell^10^

#### ^1^Cheetah Medical, Newton Center, MA, United States; ^2^Denver Health Medical Center, Denver, CO, United States; ^3^Ohio State University, Columbus, OH, United States; ^4^Royal Surrey Hospital, Guildford, United Kingdom; ^5^New York University, New York, NY, United States; ^6^Oregon Health and Sciences University, Portland, OR, United States; ^7^Emory University, Atlanta, GA, United States; ^8^New York Presbyterian Brooklyn, Brooklyn, NY, United States; ^9^Indiana University, Indianapolis, IN, United States; ^10^Massachusetts General Hospital, Boston, MA, United States

**Introduction:**

Cardiac function is known to be negatively impacted by sepsis. Monitoring cardiac output (CO) and stroke volume (SV) trends over the course of treatment may provide insight into cardiac function and predict patient outcome. The goal of this study was to explore the relationship between the change in cardiac output over time in septic shock.

**Methods:**

FRESH is a randomized controlled study evaluating the hemodynamics in critically ill patients with sepsis or septic shock (NCT02837731). Patients randomized to PLR guided resuscitation received hemodynamic monitoring for 72 or until ICU discharge, whichever occurred first (Starling SV, Cheetah Medical). Patients that exhibited an improvement in cardiac output at 12, 24, 36 and 48 hours were compared to those who did not exhibit improvement. Overall improvement in cardiac output (first CO measurement compared to last CO measurement) was also compared between groups.

**Results:**

90 patients with septic shock received hemodynamic monitoring over a 72 hour monitoring period. 60 % were female, and the average age was 61 years. Overall, 44% of assessments demonstrated a fluid responsive positive response after receiving initial resuscitation fluid of 2.3 L. Patients who exhibited improved CO at 48 hours received less fluid over the course of their ICU stay. This difference was consistent both when pre-enrollment fluids were included (5985.6 ± 2293.0 vs 8667.2 ± 3750.6, p=0.01) and only when post enrollment fluids were used (4056.2 ± 2149.2 vs 6296.5 ± 3646.8, p=0.024). Notably, patients who exhibited an overall improvement in CO also exhibited a decreased in serum creatinine over the study period (-0.28 ± 0.27 vs 0.52 ± 0.23, p=0.029) (Figure 1).

**Conclusions:**

We have previously shown that patients who improve CO in response to the resuscitation exhibited improved outcome. Trending cardiac output over the ICU stay revealed additional usefulness in predicting patients with improved outcome. The results highlight the importance of trending hemodynamics in therapy.

**Fig. 1 (abstract P535).**
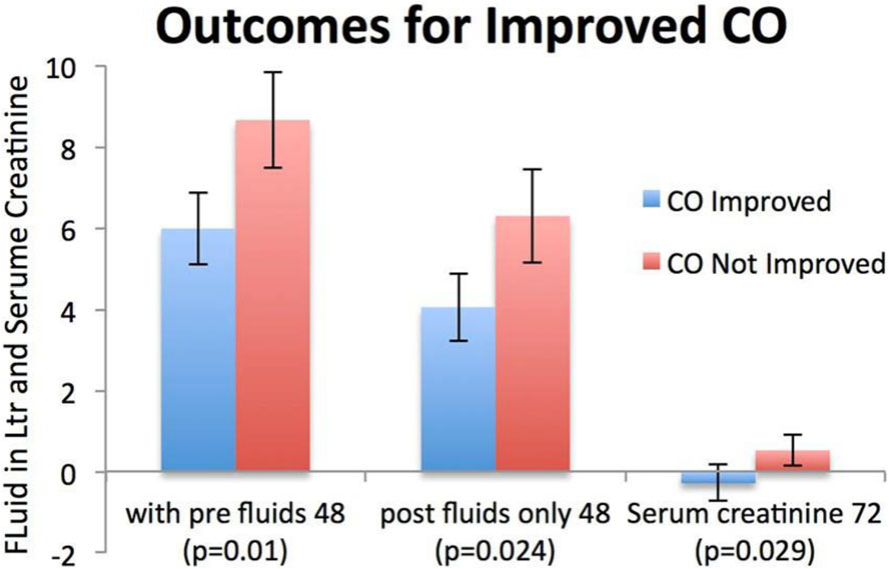
Cardiac output and patient outcome

## P536 COVID-19: speckle tracking echocardiography for detection of sepsis-induced cardiomyopathy

### J Higny^1^, F Forêt^2^, PF Laterre^3^

#### ^1^CHU UCL Namur, Department of Cardiovascular Disease, Yvoir, Belgium; ^2^CHU UCL Namur, Intensive Care Medicine, Dinant, Belgium; ^3^Cliniques Universitaires Saint-Luc, Intensive Care Medicine, Bruxelles, Belgium

**Introduction:**

SARS-CoV-2 infection may progress to acute respiratory failure (ARF), cardiac injury, renal failure and liver dysfunction. Myocardial depression in sepsis represents a major predictor of unfavorable outcome, leading to a mortality rate close to 70%. Sepsis-induced cardiomyopathy (SIC) is characterized by a global but reversible myocardial dysfunction with left ventricular (LV) dilatation and depressed LVEF [1]. While LVEF calculation is easy to acquire, it remains variable with beat to beat and highly dependent on loading conditions. Speckle tracking echocardiography (STE) is a recent imaging technology allowing early detection of LV dysfunction, prior to decrease in LVEF [2].

**Methods:**

We investigated a COVID-19 patient with severe ARDS (P/F ratio <100) and septic shock. A TTE was performed to assess LV function and standard echocardiographic variables. QLAB cardiac analysis was performed to assess strain imaging.

**Results:**

Standard echocardiographic parameters were calculated: LVEF 59.7%, LVFS 37%, LVOT VTI 22 cm, SV 56 ml, CO 7.2 l/min, E 107 cm/sec, E’ 12 cm/sec, TAPSE 15 mm, S’ 11 cm/sec, RVFAC 46%. Average strain in apical views (4-chamber, 2-chamber, 3-chamber) and global longitudinal strain (GLS) were calculated: LV strain (4-Ch) -13.4%, LV strain (2-Ch) -15%, LV strain (3-Ch) -12.4%, LV global systolic strain -13.6% (Figure 1).

**Conclusions:**

We illustrated STE findings in a COVID-19 patient admitted with hypoxemic ARF and septic shock. While LVEF was normal with the Simpson’s biplane method, average strain in 4-Ch view was -13.4% and GLS was -13.6%, indicating reduced LV longitudinal function. In this regard, STE may improve prognostication over LVEF to assess LV systolic function in critically-ill patients with SARS-Cov-2 infection.

**References**

1. Sato R et al. J Intensive Care 3:48, 2015

2. Ehrman RR et al. Crit Care 22:112, 2018

**Fig. 1 (abstract P536).**
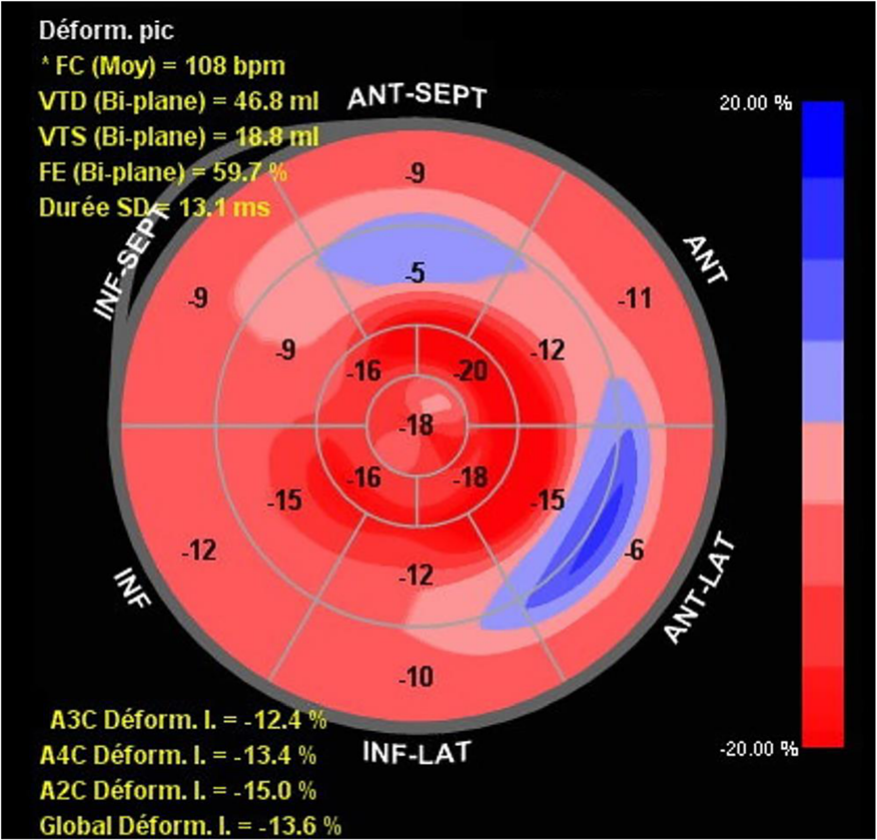
Bull’s eye plot obtained after myocardial strain imaging in a patient with severe SARS-CoV-2 infection. Apical segments are highly contractile (red color) while anteroseptal and anterolateral segments (blue color) are hypokinetic. Calculated strain in apical 4-chamber view was -13.4% and GLS was -13.6%, indicating reduced LV longitudinal function. LVEF was normal (Simpson biplane: 59.7%)

## P537 Ultrasonographic assessment of pleural effusion in patients with heart failure with preserved ejection fraction

### O Maadarani, Z Bitar, Z Bitar

#### Ahmadi Hospital, Critical Care Department, Alahmadi, Kuwait

**Introduction:**

The incidence of pleural effusions (PEs) radiologically in acute decompensated heart failure was estimated by 50 % [1]. The prevalence of PEs using chest ultrasound in patients with heart failure with preserved ejection fraction (HFpEF) was not studied before. The aim was to determine the prevalence and severity of PE using bedside chest ultrasound in patients with HFpEF.

**Methods:**

We prospectively evaluated 85 patients admitted to the coronary care unit with acute pulmonary edema. 27 patients had LVEF below 50% and they were excluded. We used bedside ultrasonography to document the presence of pleural effusions (Figure 1) and estimate the amount using Goecke 2 formula.

**Results:**

58 patients were recruited with estimated LVEF above 50% and median age of 73 years. Pleural effusion was detected by chest ultrasound in 54 patients (94%) with 91% being bilateral and of moderate amount. The mean value of E/A and E/E’ ratio was 1.92 and 19.9 respectively where mean deceleration time (DT) was 171 ms, however, these parameters were not related to severity of pleural effusion (p=0.52, 0.98 and 0.7 respectively).The N-terminal pro-brain-type natriuretic peptide (NT-proBNP) was significantly higher in patients with pleural effusion (p=0.046).

**Conclusions:**

PEs are present on chest ultrasound in 94% of patients with HFpEF and mainly bilateral. The degree of PEs was moderate and was not related to the LV indices of HFpEF.

**Reference**

1. Morales-Rull JL et al. Eur J Intern Med 52:49-53, 2018

**Fig. 1 (abstract P537).**
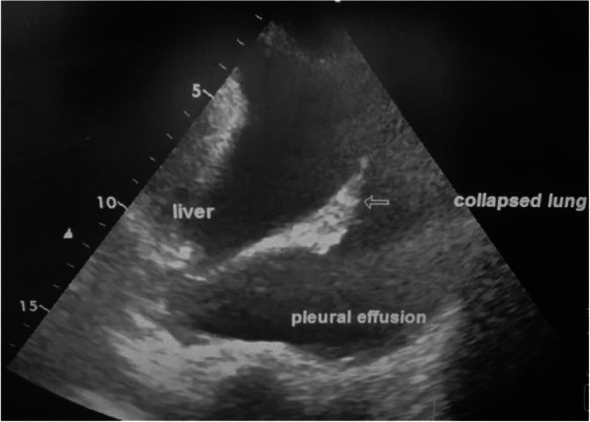
Pleural effusion

## P538 Midregional proadrenomedullin (MR-proADM) algorithm reduces hospitalization rate by identifying low risk patients in the ED safely treatable as out-patients

### J González del Castillo^1^, C Rechner^2^, C Clemente Calleja^1^, P Herrero Puente^3^, F Llopis Roca^4^, C Navarro Bustos^5^, A Schwabe^6^, J Wiemer^6^, S Ebmeyer^6^

#### ^1^Hospital Clinico San Carlos, Madrid, Spain; ^2^Thermo Fisher Scientific, BRAHMS GmbH, Hennigsdorf, Germany; ^3^Hospital Universitario Central de Asturias, Oviedo, Spain; ^4^Hospital Universitario de Bellvitge, Barcelona, Spain; ^5^Hospital Universitario Virgen Macarena, Sevilla, Spain; ^6^Thermo Fisher Scientific, Hennigsdorf, Germany

**Introduction:**

In two independent observational cohorts MR-proADM values identified low disease severity patients without risk of disease progression in the ED with no 28 days mortality that wouldn´t require hospitalization [1]. This interventional study aimed to provide guidance to safely reduce the number of hospital admissions by implementing a MR-proADM algorithm that identifies low risk patients not requiring hospitalization.

**Methods:**

A randomized controlled interventional multicenter study in 4 EDs in Spain. The study protocol was approved by the Ethics Committees of the hospitals. Control arm patients received standard care. MR-proADM guided arm patients with low MR-proADM value (≤0.87 nmol/l) were treated as out-patients, with high MR-proADM value (>0.87 nmol/l) were hospitalized (Figure 1). The hospitalization rate was compared between the study arms.

**Results:**

Two hundred patients with suspicion of infection were enrolled. In the MR-proADM guided arm the hospital admission rate in the intention-to-treat population (ITT-P) was 17% lower than in the control arm (40.6% vs. 57.6%, p=0.024) and 21% lower in the per-protocol population (PP-P) (37.2% vs. 57.6%, p=0.009). The mortality rate in the out-patients group was 0% (5.1% in hospitalized patients in ITT-P). No significant differences for the safety endpoints re-admission and re-presentation rates were observed. The re-admission rate was slightly but not significantly higher in the MR-proADM guided arm compared to the control arm (PP-P: at 14 days 9.3 % vs. 7.1%, p=1; at 28 days 11.1% vs. 9.5%, p=1). The rate of 28 days re-presentation was slightly lower in the MR-proADM guided arm compared to the control arm (20.4% vs. 26.2%, p=0.668; PP-P).

**Conclusion:**

Implementing a MR-proADM algorithm efficiently and sustaining optimizes ED workflows. Hospitals can highly benefit from a reduced rate of hospitalizations by 21% using MR-proADM.

**Reference**

1. Saeed K et al. Crit Care 23:40, 2019

**Fig. 1 (abstract P538).**
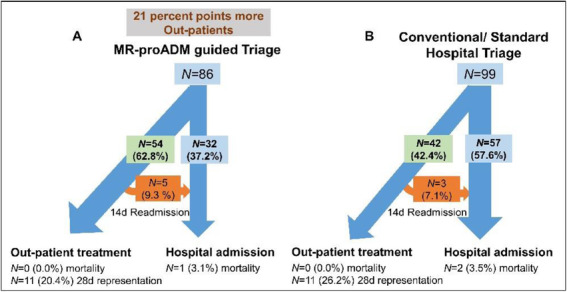
MR-proADM guided triage in comparison to conventional/standard hospital triage. Decisions on out-patient treatment or hospitalization after initial Emergency Department assessment illustrated for the PP population, with corresponding 14d readmission rates, 28d representations rates and 28d mortality rates. Panel A: MR-proADM guided triage, Panel B: Conventional/Standard Hospital Triage. d: day, PP: per-protocol, MR-proADM: mid-regional proadrenomedullin, N: number

## P539 DNA methylation is altered in muscle of critically ill patients

### L Van Dyck^1^ , F Güiza^2^, I Derese^2^, L Pauwels^2^, MP Casaer^2^, G Hermans^2^, PJ Wouters^2^, G Van den Berghe^2^, I Vanhorebeek^2^

#### ^1^KU Leuven, Laboratory of Intensive Care Medicine, Leuven, Belgium; ^2^KU Leuven, Leuven, Belgium

**Introduction:**

ICU-acquired weakness has shown to persist beyond the ICU stay and to associate with long-term functional impairment of ICU survivors. Underlying mechanisms remain unclear, but illness-induced aberrant DNA methylation could be involved due to its potential long-lasting impact on gene expression. Recently, DNA methylation alterations have been identified in peripheral blood of pediatric ICU patients, which were found to explain part of their long-term developmental impairment [1]. Whether DNA methylation in muscle is altered by critical illness is unknown.

**Methods:**

We extracted DNA from skeletal muscle biopsies collected on day 8±1 in ICU from patients included in the EPaNIC trial (n=188) and from 20 matched healthy controls. Genome-wide DNA methylation was determined with Infinium® HumanMethylation EPIC-BeadChips, which interrogate more than 850000 CpG sites. Methylation status of individual CpG sites and of DNA regions of patients and controls were compared, using stringent corrections for multiple comparisons.

**Results:**

In DNA extracted from ICU patients, 565 CpG sites, associated with 400 unique genes, were differentially methylated as compared with controls, with an average difference in methylation of 3.2% (SEM 0.07%, p<0.00005). Many of the associated genes were identified as highly relevant for muscle structure, function and/or weakness. In addition, in patients as compared with controls, we identified two hypomethylated regions (family-wise error rate <0.05), spanning 18 and 3 CpG sites in the promotor regions of the HIC1 and NADK2 genes, respectively. HIC1 and NADK2 play important roles in muscle regeneration and postsynaptic acetylcholine receptor regulation, and in mitochondrial processes, respectively.

**Conclusions:**

The DNA methylation signature in skeletal muscle is altered by critical illness, which may provide a biological basis for the long-term persistence of weakness in ICU survivors.

**Reference**

1. Güiza F et al. Lancet Respir Med 8:288-303, 2020

## P540 Complications after abdominal surgery and their impact on postoperative outcome

### V Shpata^1^, E Ramosaço^2^, N Kodra^3^

#### ^1^Faculty of Medical Technical Sciences, University of Medicine in Tirana, ICU, Tirana, Albania; ^2^Faculty of Medical Technical Sciences, University of Medicine in Tirana, Tirana, Albania; ^3^University Hospital Center of Tirana, Tirana, Albania

**Introduction:**

Postoperative morbidity still remains a major concern for the survival and cost of treatment, negatively affecting either of them. The aim of the study to describe the most common complications after abdominal surgery (POC) and their impact on length of hospital stay (LOS) and in-hospital mortality. We also explored which patients were more at risk for these complications.

**Methods:**

Prospective observational study on patients who underwent abdominal surgery in University Hospital Center of Tirana that stayed ≥ 3 days in hospital.

**Results:**

540 patients, aged 62.3±12.5 years old, 58.9% male. 40.74% of the patients developed at least one POC, the most common POC were: pulmonary 30.7%, cardiovascular 16.7% and surgical complications 15.2%. Patients with POC had longer hospital stay compared to patients without those complications: 10.22±5.71(3-40) days versus 6.28±2.88 (3-15) days, p<0.0001. In-hospital mortality resulted 8.3%. All the patients with mortality had at least one POC. Risk for mortality was higher in patients with complications compared to patients without respective complications, such as: cardiovascular complications (OR: 10.28, 95%CI: 5.35-19.74), surgical complications (OR: 9.60, 95%CI: 5.01-18.39) and pulmonary complications (OR: 280.48, 95%CI: 17.14-4587.48), p - significant. Multivariate analysis revealed that independent risk factor for POC were diabetes mellitus (OR:2.93, 95%CI:1.63-5.27, p=0.0003), hemoglobin levels < 10 g/dl (OR:2.15, 95%CI:1.25-3.71, p=0.005), previous lung disease: (OR:3.40, 95%CI:1.59-7.27, p=0.001), emergency admission: (OR: 6.47, 95%CI:2.01-20.76, p=0.001).

**Conclusions:**

Complications are frequent after abdominal surgery (incidence: 40.74%) and the most common complications were pulmonary complications. Postoperative complications increase length of hospital stay and in-hospital mortality. Efforts should be done to prevent complications, modifying their risk factors and optimizing the treatment of underlying diseases.

## P541 Predicting risk of emergency readmission after intensive care: external validation of an existing model

### C Beattie^1^, P Henderson^2^, T Quasim^2^, M Shaw^3^

#### ^1^University of Glasgow, School of Medicine, Glasgow, United Kingdom; ^2^University of Glasgow, Glasgow, United Kingdom; ^3^Glasgow Royal Infirmary, Glasgow, United Kingdom

**Introduction:**

Intensive care unit (ICU) survivors are at risk of emergency hospital readmissions. A risk prediction tool may help identify high-risk patients in need of preventative interventions after discharge. Scottish Patients at Risk of Readmission and Admission (SPARRA) predicts 1-year risk of emergency hospital admission in the general Scottish population. The aim of this study was to externally validate SPARRA in ICU survivors. We also aimed to compare the performance of the two SPARRA scoring systems: automated scores (imported from national database) and manual scores (generated by entry of predictors into online risk calculator).

**Methods:**

Retrospective study of 264 patients discharged from a single ICU from 2015-2018. Scores were matched to time of hospital discharge: automated scores were obtained for all 264 participants; 60 patients were also scored manually. Observed rates of emergency admission in the year from hospital discharge were recorded. Discrimination was assessed by area under the ROC curve (AUC); calibration plots were drawn and the mean absolute error (MAE) between predicted and observed readmission rates was calculated.

**Results:**

160 patients (60.6%) had ≥1 emergency admission in the follow-up year. Manual scores had poor discrimination: AUC 0.59 (95% CI 0.44-0.74); automated SPARRA scores had moderate discrimination: AUC 0.73 (95% CI 0.65-0.81). The ROC curves are shown in Figure 1. Both scoring systems were poorly calibrated with underestimation of admission rates across all risk strata. The MAE was 16.5% (95% CI 10.2-22.8) in automated scores and 20.9% (95% CI 13.8-28.0) in manual scores.

**Conclusions:**

Emergency hospital readmissions are common in the year after hospital discharge following an intensive care admission. Automated SPARRA scores performed better than manual SPARRA scores in parameters of discrimination and calibration. However, neither method reliably predicted emergency readmission in this cohort. A new risk prediction model should be developed for use in ICU survivors.

**Fig. 1 (abstract P541).**
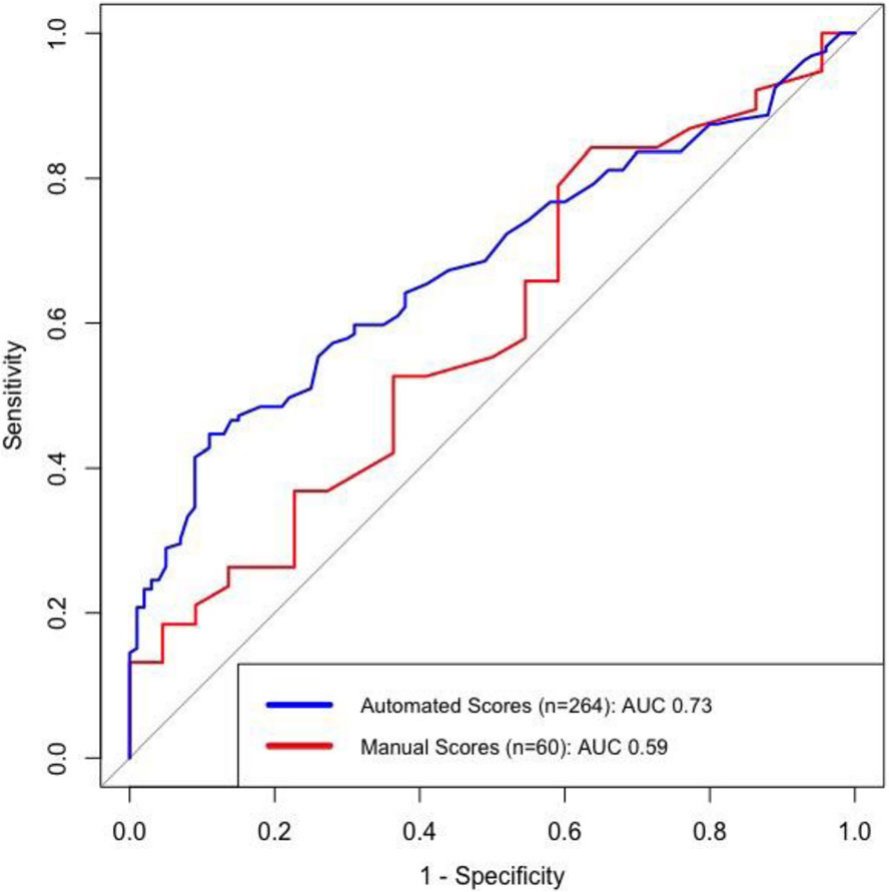
Receiver operating characteristic (ROC) curves for automated SPARRA scores (n=264) and manual SPARRA scores (n=60)

## P542 Impact of frailty status on timely goals of care documentation and clinical outcomes in older patients

### A Subramaniam^1^, R Tiruvoipati^1^, C Green^2^, M Bailey^3^, D Pilcher^4^

#### ^1^Peninsula Health, Intensive Care Unit, Mt Eliza, Australia; ^2^Peninsula Health, ANZRIC, Mt Eliza, Australia; ^3^Alfred Health, School of Public Health and Preventive Medicine, Melbourne, Australia; ^4^Alfred Health, Intensive Care Unit, Melbourne, Australia

**Introduction:**

Hospitalized frail older patients are at risk of clinical deterioration. Early goals of care documentation (GOC) is vital to avoid futile/unwarranted interventions in the event of deterioration. It is however unclear whether being frail impacted GOC timing. We aimed to investigate the impact of frailty, as measured by Hospital Frailty Risk Score (HFRS), on timely GOC and outcomes in all newly admitted older patients.

**Methods:**

Single-center retrospective study of all medical patients aged ≥80 years admitted between 1/3/2015 and 31/8/2015. Primary outcome was rates of GOC within 72-hours in frail (HFRS≥5) and non-frail (HFRS<5) patients. Secondary outcomes included GOC during hospital stay, in-hospital mortality, rapid response call (RRC), discharge destination, and 28-day readmission rate.

**Results:**

529 (47.3%) of the 1,118 admitted patients were frail. Frailty syndromes frequented reasons for hospitalization in frail patients (55.2% vs. 26.7%; p<0.001). 604 (54%) had GOC during their hospitalization; 559 (92.5%) of these within 72-hours (53.5% vs. 46.9%; p<0.027), commonly in frail patients. Frail patients had higher proportion of RRCs (12.5% vs. 5.4%, p<0.001), in-hospital mortality (10.8% vs 3.6%, p<0.001), longer hospitalizations (median 5.3 vs 2.9 days, p<0.001) and were less likely to be discharged to their usual accommodation (32.3% vs 57.7%; p<0.001) than non-frail patients. There was no difference in 28-day readmission rates (6.6% vs. 8.5%; p=0.24).

**Conclusions:**

Older frail patients were more likely to have timely GOC than older non-frail patients. Frail patients had more RRCs, longer hospitalization and increased hospital mortality. Early GOC may avoid burdensome treatments.

## P543 Critical management of robotic pancreatic surgery: our experience on the first 66 procedures

### K Donadello^1^, C Bruscagnin^2^, L Gottin^2^, M Taiana^2^, V Schweiger^2^, E Polati^2^

#### ^1^University of Verona, School of Medicine, Anesthesia and Intensive Care B, Department of Surgery, Dentistry, Gynecology and Pediatrics, Verona, Italy; ^2^University of Verona, School of Medicine, Verona, Italy

**Introduction:**

Pancreatic robotic surgery (PRS) allows less invasivity and a better anatomical view than open surgery. From the anesthesiological point of view, this approach requires greater attention to hemodynamics and ventilation as complications can occur during or after surgery. This observational study aimed at evaluating the first 2 years of PRS procedures so as to foster a common anesthesiological management.

**Methods:**

We analyzed the data of patients submitted to PRS between December 2017 to January 2020. Standard general anesthesia was performed (induction with propofol, opioid and rocuronium for neuromuscular blockade, maintenance with alogenate or TIVA/TCI), using VCV or PCV-VG ventilation; vital signs and TOF were constantly monitored intra-operatively. We recorded baseline clinical characteristics, pre-operative SOFA score, positive pre-operative surveillance rectal swabs, clinical(AKI, pneumonia, fever and/or sepsis) and surgical complications (pancreatic fistulae, abdominal collections, bleeding); 1st post-operative day(POD) SOFA score; ICU and hospital length of stay(LOS).

**Results:**

66 pts(14-77 y, 21 males) were analyzed, ASA 1-3,mean BMI 20.3 kind of interventions were performed: 5 enucleoresections, 27 spleen preserving distal pancreatectomy, 34 distal splenopancreatectomy. Mean duration of surgery was 426’ with a mean laparoscopic IAP of 12 mmHg. TAP block was performed in 21 pts. Pre-operative SOFA score was 0 for all patients. Pre-operative rectal swabs showed 1 case of *E. coli* ESBL. Pre- and PO SOFA score were not statistically different.9 patients were admitted to ICU with maximum LOS of 5 days. Mean LOS in the hospital was 11 days. 65% of patients had complications (21 sepsis, 23 fistulae, 16 abdominal collections, 5 PO bleeding, 4 pneumonias, 2 pneumothorax, 1 AKI). We recorded 1 case of PO positive rectal swab (*C. freudii* ESBL) and 3 cases of MRSA bloodstream infection.

**Conclusions:**

IO management did not alter pts' SOFA score but postoperative complications prolonged both ICU and hospital LOS.

## P544 Arterial lactate concentration on intensive care unit admission predicts long term mortality after liver transplantation

### LD Drouard

#### CHU Erasme, Anesthesiology, Brussels, Belgium

**Introduction:**

Arterial lactate is a routinely measured biomarker that reliably detects tissue hypoxia. High risk patients undergoing major surgery are at risk of developing hyperlactatemia. The aim of this study was to investigate the association between arrival ICU lactate concentration and mortality at 1 year, postoperative complications and 30-day mortality after liver transplantation.

**Methods:**

We identified all liver transplant patients in Erasme hospital from September 2013 to December 2019.The primary outcome was to determine if early hyperlactatemia was associated with mortality at one year. Other outcomes included the association between increased lactate, postoperative complications and 30-day mortality. A multivariate analysis determined the independent association of lactate on one year mortality. Receiver operating characteristic (ROC) curves were established for one-year mortality, 30-day mortality, and postoperative complications. 95% IC were calculated with the Delong method.

**Results:**

A total of 228 patients were included from September 21st, 2013, to December 19th, 2019. Lactate on ICU arrival predicted long term mortality at one year with an ROC AUC of 0.80 (95% CI 0.72-0.87). Lactate values greater than 1.75, 3, and 5 mmol/l were associated with a 50%, 75%, and 90% risk of death at 1 year, respectively. Furthermore, lactate on ICU arrival predicted 30-day mortality with an ROC AUC of 0.91 (95% CI 0.84 - 0.97). Lactate values greater than 2, 5, and 6 mmol/l were associated with a 49%, 73%, and 90% risk of mortality at 30 days post-transplant. ROC AUC for predicting complications, however, was only 0.6 (95% CI 0.56 - 0.71).

**Conclusions:**

In this retrospective study we report an association between early postoperative lactate level and major postoperative complications, 30-day mortality, and 1 year mortality. Lactate concentration on ICU arrival is an easily accessible biomarker that may predict outcome and identify high risk patients.

## P545 STAR-Liège: modulating insulin AND nutrition improves glycemic control

### V Uyttendaele^1^, JL Knopp^2^, M Pirotte^3^, M Bayet^3^, P Morimont^3^, N Layios^3^, B Lambermont^3^, GM Shaw^4^, JG Chase^2^, T Desaive^1^

#### ^1^University of Liège, GIGA - In silico Medicine, Liège, Belgium; ^2^University of Canterbury, Department of Mechanical Engineering, Christchurch, New Zealand; ^3^University Hospital of Liège, Department of Intensive Care, Liège, Belgium; ^4^Christchurch Hospital, Department of Intensive Care, Christchurch, New Zealand

**Introduction:**

Stress-induced hyperglycemia is a common complication associated with higher morbidity and mortality in ICU patients. The Stochastic TARgeted (STAR) glycemic control (GC) framework provides consistent, safe, effective control in different ICUs and countries. It is a patient-specific, risk-based protocol controlling both insulin and nutrition dosing. This study analyses safety and efficacy of STAR at the University Hospital of Liège, Belgium, and assesses the impact of also modulating nutrition on GC outcomes.

**Methods:**

Patients are included after 2 blood glucose (BG) > 145 mg/dl. The study compares STAR (N=14; modulating insulin and nutrition) and STAR-IO (N=15; controlling insulin only, leaving nutrition at clinical discretion). STAR controls nutrition between 30-100% of goal feed if insulin alone cannot safely reduce BG. The target band is 80-145 mg/dl for both arms. GC was stopped after 72h or if BG in target at insulin rate ≤2U/h for 6 hours. Performance is assessed by %BG in target and the median [IQR] BG, and safety by %BG below (<80 mg/dl) and above (>145 mg/dl) target. Ethics approval was granted by the University Hospital of Liège Ethics Committee.

**Results:**

Table 1 shows better performance for STAR (83% vs 78% BG in target), and better safety from hypoglycemia (0.7% vs 1.5%) and hyperglycemia (17% vs 21%). Median [IQR] BG are similar (STAR: 121 [110 135] mg/dl vs STAR-IO: 119 [106 137] mg/dl). Despite controlling nutrition, STAR provided higher median [IQR] nutrition than STAR-IO (98 [67 109] vs 93 [53 103] %Goal) and required lower workload (13.6 vs 15.5 assays per day).

**Conclusions:**

STAR outperforms STAR-IO. Both STAR and STAR-IO provide safe, effective control for all patients. GC safety and performance can be improved by patient-specific control of nutrition, in addition to insulin, for highly insulin resistant patients.

**Table 1 (abstract P545).** Clinical data GC results for STAR and STAR-IO

	STAR (N=14)	STAR-IO (N=15)
Workload (BG assays/day)	13.6	15.5
Median [IQR] BG (mg/dl)	121 [110 135]	119 [106 137]
% BG in 80-145 mg/dl (%)	83	78
% BG > 145 mg/dl	17	21
% BG < 80 mg/dl	0.7	1.5
# Patients < 40 mg/dl	0	0
Median [IQR] nutrition (%Goal)	98 [67 109]	93 [53 103]

## P546 Higher insulin resistance in female ICU patients

### V Uyttendaele^1^, JL Knopp^2^, GM Shaw^3^, T Desaive^1^, JG Chase^2^

#### ^1^University of Liège, GIGA - In silico Medicine, Liège, Belgium; ^2^University of Canterbury, Department of Mechanical Engineering, Christchurch, New Zealand; ^3^Christchurch Hospital, Department of Intensive Care, Christchurch, New Zealand

**Introduction:**

Sex differences in the metabolic response to critical illness are unknown. This retrospective analysis examines potential differences in the evolution of insulin sensitivity (SI) and its variability (%ΔSI) between sexes. Significant differences would suggest differences in the metabolic stress response and glycemic response to insulin therapy, and, thus, the need for more personalized glycemic control (GC).

**Methods:**

Retrospective data from 145 ICU patients (N=8710 hours) are used to hourly identify hourly model-based SI and its rate of change %ΔSI in 6-hour blocks from ICU admission to 72 hours. The evolution of SI and %ΔSI are compared for males and females. Hypothesis testing (95% confidence interval (CI) bootstrapped difference in medians) assesses if differences are significant, and equivalence testing assesses if differences are clinically equivalent.

**Results:**

Females have significantly lower SI levels than males (p<0.05), and this difference is not clinically equivalent (Figure 1; top). Differences in %ΔSI are not significant (p>0.05), and these differences are clinically equivalent (Figure 1; bottom).

**Conclusions:**

Given significantly lower SI levels, but equivalent variability, for women, equally safe and effective GC should be achievable for both sexes. However, women may require more insulin to achieve these goals. GC protocol designs should thus account for these differences in the future.

**Fig. 1 (abstract P546).**
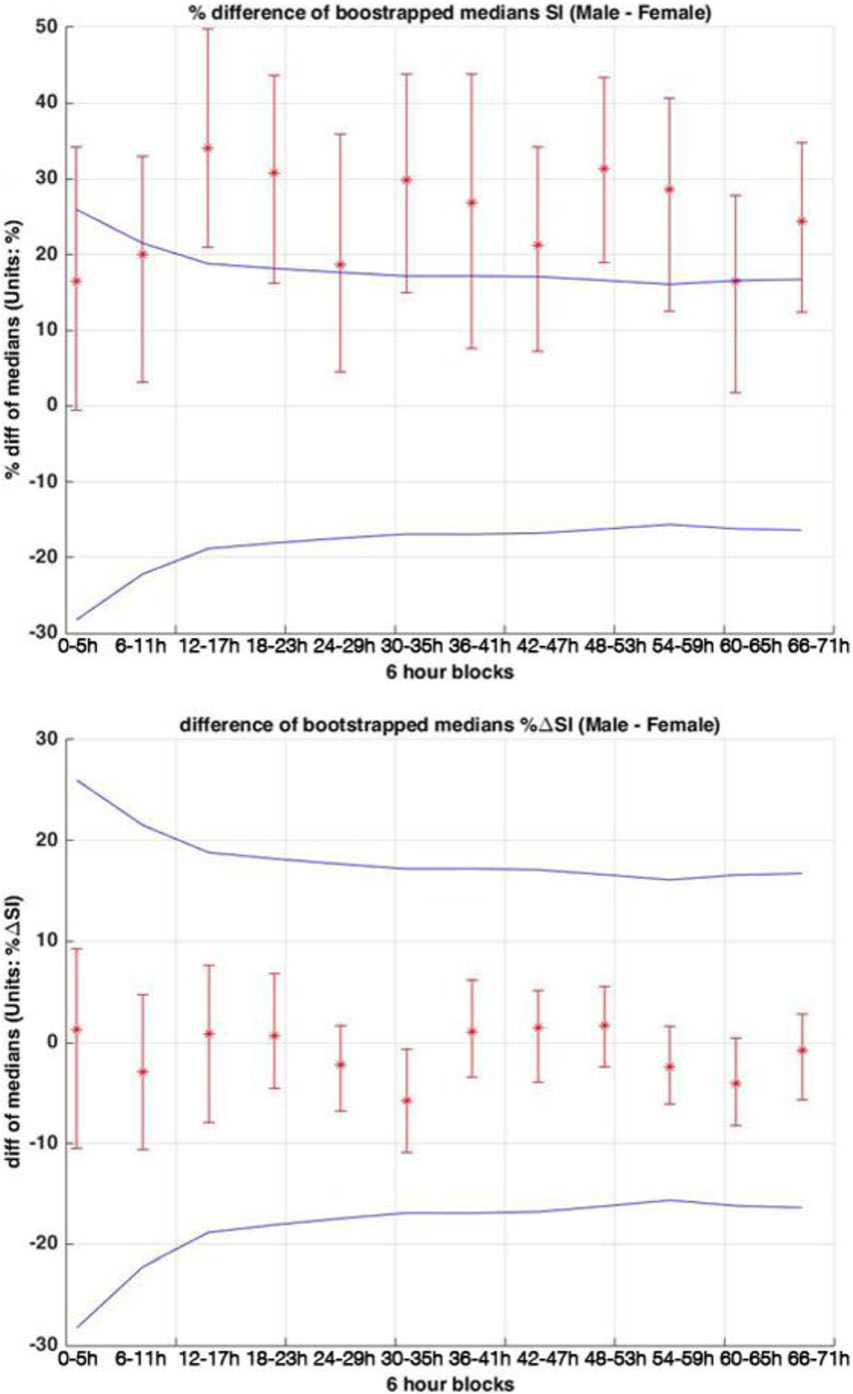
Hypothesis and equivalence testing results on SI (top) and %ΔSI (bottom) between males and females for each 6-h block. Blue solid lines give equivalence range. Equivalence accepted if 95% CI of percentage difference in bootstrapped median SI values is within equivalence range. Difference in median SI and %ΔSI levels are considered statistically significant (p<0.05) if the 95% CI of difference in bootstrapped medians SI and %ΔSI crosses 0

## P547 Reduced workload in the STAR glycemic control framework: quantifying the safety trade-off

### V Uyttendaele^1^, JL Knopp^2^, GM Shaw^3^, T Desaive^1^, JG Chase^2^

#### ^1^University of Liège, GIGA - In silico Medicine, Liège, Belgium; ^2^University of Canterbury, Department of Mechanical Engineering, Christchurch, New Zealand; ^3^Christchurch Hospital, Department of Intensive Care, Christchurch, New Zealand

**Introduction:**

The Stochastic Targeted (STAR) glycemic control (GC) framework is a model-based, risk-based insulin and nutrition protocol providing safe, effective GC to nearly all patients. STAR currently uses 1-3 hourly measurement intervals, averaging 12 measurements per day. This study assesses the impact of increasing its measurement interval up to 6-hourly on GC safety and efficacy.

**Methods:**

STAR identifies patient-specific model-based insulin sensitivity (SI) using clinical data and forecasts its future variability to obtain an insulin and nutrition intervention minimizing hypoglycemic risk. STAR also modulates nutrition intervention to reduce persistent hyperglycemia for highly insulin resistant patients. STAR is adapted to allow 1-6 hourly measurement interventions. Validated virtual trials assess GC outcomes of the original STAR (STAR-3H) and the STAR 1-6 hourly (STAR-6H) protocols on the same 681 underlying virtual patients based on retrospective clinical data.

**Results:**

Results are presented in Table 1. As expected, STAR-6H results in lower workload than STAR-3H (8 vs 12 measures per day). The resulting median [IQR] BG is higher in STAR-6H (124 [113 139] mg/dl) compared to STAR-3H (117 [106 131] mg/dl), using significantly lower insulin (2.5 [1.5 3.0] vs 3.2 [2.0 5.0] U/h). Overall, there is a slight decrease in the %BG in target band (80-145 mg/dl) for STAR-6H (80% vs 83%), and slightly higher %BG above target (18% vs 15%). Importantly, the incidence of moderate hypoglycemia is similar (1.6%), but STAR-6H has higher incidence of severe hypoglycemia (19 (2.8%) vs 14 (2.1%) patients). These results are achieved with slightly lower nutrition for STAR-6H (90 [75 100] vs. 100 [85 100] %goal).

**Conclusions:**

The risks associated with the reward of reducing workload are slightly reduced safety, performance, and nutrition rates. Overall, despite using these longer measurements intervals, STAR still managed to provide highly safe, effective GC for nearly all patients.

**Table 1 (abstract P547).** Virtual trial results of STAR-3H and STAR-6H on 681 virtual patients. Data given as median [IQR] for per-patient statistics

	STAR-3H (N=681, totaling 59240h)	STAR-6H (N=681, totaling 60003h)
Workload (meas. per day)	12	8
Per-patient median BG (mg/dl)	117 [106 131]	124 [113 139]
Per-patient median insulin (U/h)	3.2 [2.0 5.0]	2.5 [1.5 3.0]
Per-patient median nutrition (%goal)	100 [85 100]	90 [75 100]
%BG in 80-145 mg/dl	83	80
%BG>145 mg/dl	15	18
# patients min BG < 40 mg/dl	14 (2.1%)	19 (2.8%)

## P548 Improved risk-based glycemic control for critically ill patients: the first 200 patients

### V Uyttendaele^1^, JL Knopp^2^, GM Shaw^3^, T Desaive^1^, JG Chase^2^

#### ^1^University of Liège, GIGA - In silico Medicine, Liège, Belgium; ^2^University of Canterbury, Department of Mechanical Engineering, Christchurch, New Zealand; ^3^Christchurch Hospital, Department of Intensive Care, Christchurch, New Zealand

**Introduction:**

Stochastic TARgeted (STAR) is unique patient-specific, model-based, and risk-based glycemic control (GC) framework providing safe, effective control for virtually all patients, targeting the 80-145 mg/dl range. STAR uses a proven physiological model and a stochastic model to identify patient-specific insulin sensitivity (SI) from clinical data and its likely future evolution in the next 1-3 hours. These predicted SI ranges can be used to predict blood glucose (BG) outcomes for a given insulin and nutrition treatment to maximize nutrition and minimize (mild) hypoglycemic risk. This study presents an initial safety and performance analysis of STAR using an enhanced 3D (STAR-3D) risk-prediction model from use as the new standard of care at Christchurch Hospital, New Zealand.

**Methods:**

STAR is unique as it modulates both insulin and nutrition inputs. In total, 200 GC episodes are analyzed for performance and safety. This data audit and analysis was approved by the New Zealand Health and Disability Ethics Committee Upper South Regional Ethics Committee B (Ref: URB/07/15/EXP).

**Results:**

Results are presented in Table 1. STAR provided high GC efficacy with median [IQR] per-patient %BG in target band of 81 [65 92]%, with minimal incidence of mild hypoglycemia overall 0.3%BG<80mg/dl and no incidence of severe hypoglycemia (BG<40 mg/dl). The resulting median [IQR] 119 [112 130] mg/dl per-patient median BG is achieved with median [IQR] 3.5 [2.5 5.0] U/h of median insulin and 97 [80 100] median % goal feed. Considering results only once the target band is reached (145 mg/dl) to avoid bias from different initial starting BG, both performance and safety are further improved (Table 1).

**Conclusions:**

STAR using this new 3D predictive model provides safe, effective control for all patients, with no incidence of severe hypoglycemia, despite targeting normoglycemic ranges. Results are slightly better than the current standard of care version of STAR.

**Table 1 (abstract P548).** Per-patient clinical GC outcomes summary for STAR-3D. Data given as median [IQR] where appropriate

	STAR-3D (N=200)	STAR-3D (once target reached, N=199)
Median BG (mmol/l)	119 [112 130]	115 [108 124]
% BG in 80-145 mg/dl (%)	81 [65 92]	95 [82 100]
% BG > 145 mg/dl (%)	18 [7 33]	3 [0 15]
% BG < 80 mg/dl (%)	0 [0 0]	0 [0 0]
# Patients < 40 mg/dl (%)	0	0
Median insulin rate (U/h)	3.5 [2.5 5.0]	2.5 [1.5 4.0]
Median dextrose rate (%GF)	97 [80 100]	95 [75 100]

## P549 Perioperative blood transfusion during cytoreductive surgery with hyperthermic intraperitoneal chemotherapy: an observational study

### G Madrid^1^, O Ballesteros^2^, JA Cárdenas^2^, E Celis^2^

#### ^1^Fundación Santa Fe de Bogotá, Anesthesiology, Bogotá, Colombia; ^2^Fundación Santa Fe de Bogotá, Critical Medicine and Intensive Care, Bogotá, Colombia

**Introduction:**

The increase in morbidity and mortality associated with anemia has been well documented. Thus, blood transfusion can be a lifesaver maneuver for the surgical patient. However, transfusion carries a potential risk of acute or delayed immunologic reactions that may result in poor postoperative outcomes. Patients undergoing cytoreductive surgery with hyperthermic intraperitoneal chemotherapy (CRS/HIPEC) may have a higher risk of bleeding due to tumor-related fibrinolysis, tumor vascularity and location, and extent of the disease. We aimed to evaluate the trends in transfusion rates for CRS/HIPEC.

**Methods:**

After IRB approval was obtained, a retrospective observational study was conducted. All patients older than 18 years undergoing elective CRS/HIPEC were enrolled. We excluded patients with known coagulopathies and active infection. Main outcome was to describe transfusion rates in patients undergoing CRS/HIPEC. Other variables measured included ICU length of stay, mechanical ventilation, readmission to ICU, and hospital length of stay. For analysis patients were divided by the amount of blood units received: 0 units, ≤4 units, and >4 units. Chi-squared test, Student’s T-test and Mann-Whitney U test were used when appropriate. P<0.05 was considered statistically significant.

**Results:**

A total of 130 patients were included, of which 114 patients (87.7%) had blood transfusion. Of these, 57 patients (50%) received ≤4 red blood cells units and 49 patients (37.7%) received >4 red blood cells units. The average number of red blood cells units transfused per person was 4.23 ± 3.29. There were statistically significant differences on duration of mechanical ventilation (p=0.036) and ICU length of stay (p<0.01). No significant differences were found on hospital length of stay, start of oral feeding, ambulation, and readmission to ICU between groups (p>0.05).

**Conclusions:**

Perioperative blood transfusion of >4 units may associate with increased length of stay in ICU and duration of mechanical ventilation.

## P550 Coagulogram changes and von Willebrand factor activity in patients with COVID-19 infection.

### S Tachyla^1^, A Marochkov^1^, D Tsopau^2^, V Kononkov^2^

#### ^1^Mogilev Regional Hospital, Department of Anesthesiology and Intensive Care, Mogilev, Belarus; ^2^Mogilev hospital №1, Department of Anesthesiology and Intensive Care, Mogilev, Belarus

**Introduction:**

Pandemic COVID-19 lasts for several months and associated with high mortality. Features of the pathogenesis of this disease are not well understood. Depth laboratory tests - this is the key to the study of this disease. The objective was to determine the changes in coagulation parameters and activity of von Willebrand factor in patients with infection COVID-19.

**Methods:**

A pilot cohort study was conducted in 6 patients of the intensive care unit, of which 4 men and 2 women, age 58 (56-68) years, weight 91 (79-98) kg, height 169 (167-174) cm. The presence of COVID-19 infection in patients was established by polymerase chain reaction diagnostics and computed tomography of the chest. Blood sampling for analysis was performed on the 1st day of admission to the intensive care unit. A coagulometer ACL 10000 (USA) was used. The activated partial thromboplastin time (APTT), prothrombin time (PT), prothrombin complex activity, international normalized ratio (INR), fibrinogen, D-dimers, plasminogen, antithrombin III were monitored. Von Willebrand factor activity indicators were determined using the mouse monoclonal antibodies.

**Results:**

All patients had acute respiratory distress syndrome and mechanical ventilation. It was not dead among them. All patients received enoxoparin at 4000 anti-XA IU/day. Other drugs that affect coagulation, and blood plasma is not administered. When studying the indicators of the coagulogram, the values of APTT are 35.2 (33.4-37.2) s, PT 16.2 (15.4-16.7) s, INR 1.18 (1.1-1.2) and fibrinogen 4.8 (4.3-5.3) g/l were slightly higher than normal. Moreover, in patients Antithrombin III was decreased to 77.9 (66.4-86.9)% (normal over 83%), the level of D-dimers was increased 0.65 (0.43-2.14) μg / ml (normal to 0.26 μg/ml), and was increased activity of von Willebrand factor 364.5 (331.5-456.8)% (normal to 125.2% -169.7%).

**Conclusions:**

In patients with COVID-19 infection antithrombin III was increased, the level of D-dimers was increased, and the activity of von Willebrand factor was increased.

## P551 Preoperative anemia increases risk for poor outcome after abdominal surgery

### V Shpata^1^, M Kreka^2^, T Çina^2^

#### ^1^Faculty of Medical Technical Sciences, University of Medicine in Tirana, ICU, Tirana, Albania; ^2^Faculty of Medical Technical Sciences, University of Medicine in Tirana, Tirana, Albania

**Introduction:**

Anemia is a common finding in preoperative period, and often increases the need for blood transfusion. The aim of the study: to explore the relation between moderate or severe preoperative anemia (Hb levels <10 g/dl) and postoperative outcome and also the impact of preoperative blood transfusion on the outcome.

**Methods:**

Prospective observational study on patients, who underwent abdominal surgery in University Hospital Center of Tirana, that stayed more than 48 hours in hospital. Univariate and multivariate analysis used to determine relation between variables.

**Results:**

The study included 540 patients. 107 patients, 19.6% had Hb levels <10 g/dl. 63.55% of the patients with anemia developed at least one postoperative complication demonstrating a greater risk for their occurrence (OR: 3.22, 95%CI: 2.07-5.00) and increased in-hospital mortality (OR: 4.55, 95%CI:2.42-8.55). Length of hospital stay was longer for patients with preoperative anemia compared to them without anemia, respectively: 9.86±5.92 days, versus 7.40±4.19 days, p<0.0001. Multivariate analysis adjusted for confounders showed that moderate and severe preoperative anemia was an independent risk factor for the occurrence of postoperative complications (OR: 2.15, 95%CI: 1.25-3.71), especially for surgical complications (OR: 2.93, 95%CI: 1.55-5.52). 50 patients (9.25%) received preoperative blood transfusion, which resulted in an increased risk for postoperative complications (OR: 6.88, 95%CI: 3.36-14.10) and in-hospital mortality (OR: 10.93, 95%CI: 5.46-21.9), compared to patients who did not receive blood transfusion. (p-value significant)

**Conclusions:**

19.6% of patients had moderate or severe preoperative anemia. Anemia resulted an independent risk factor for postoperative complications and it increased the length of hospital stay and in-hospital mortality. A better preoperative treatment of anemia, avoiding blood transfusion, can improve postoperative outcome of the patients.

## P552 Pushing the limits of storage of venous blood gas samples from intensive care patients

### MK Pedersen^1^, M Lumholdt^2^, SK Fagerberg^2^, S Mikkelsen^3^, P Leutscher^4^, K Damgaard^5^

#### ^1^Centre for Clinical Research, North Denmark Regional Hospital, Hjørring, Denmark; ^2^Aalborg University Hospital, Department of Anaesthesiology and Intensive Care, Aalborg, Denmark; ^3^University of Southern Denmark, The Prehospital Research Unit, Odense, Denmark; ^4^Centre for Clinical Research, Hjørring, Denmark; ^5^North Denmark Regional Hospital, Department of Anaesthesiology and Intensive Care, Hjørring, Denmark

**Introduction:**

The blood gas sample is the most used paraclinical test at the ICU and recommended to be analyzed instantly without any storage. At a clinical setting with acute and unexpected work tasks, blood gas samples might be left unanalyzed and, consequently, replaced by a new patient sample. We hypothesize that minor storage with delayed analysis is unproblematic. We aim to define the timespan venous blood gas samples maintain stable values. The study is ongoing.

**Methods:**

From each of ten healthy participants we have obtained 16 blood samples. This is followed by an ongoing inclusion of ICU patients with an abnormal pH, and we increase the number of samples to 20. Sampling is done through an intravenous access (central or elbow flexion), a three-way stopcock and 1.8 ml heparinized syringes. Samples are either packed in separate ice bags or stored at room temperature. The ABL800 FLEX analyses samples for pH, pO2, pCO2, HCO3-, glucose and lactate. The first and last obtained samples are analyzed immediately and used as a reference, followed by a cooled and un-cooled sample every 15th-18th minute. In the case of the ICU samples, the test period is prolonged from two to three hours.

**Results:**

The number of currently included ICU patients (nine) does not meet a sufficient sample size for statistics. However, preliminary results tend towards the results of the normal values; Except lactate, all normal values remain stable throughout a two-hour test period without any differences compared to controls (un-paired Mann-Whitney U-test; all p>0.05). Apart from pO2, there are minor clinically irrelevant effects of cooling (paired Mann-Whitney U-test, n=70; all p<0.001). In the case of lactate, cooling delays an otherwise instantly increase for one hour and also slows down the progression (paired Mann-Whitney U-test; p<0.001).

**Conclusions:**

Preliminary results of stored abnormal venous blood gas values reflect the trends of normal values. Except for lactate, normal venous blood gas values remain stable throughout two hours.

## P553 Does curative intravenous immunoglobulin therapy improve outcome in the treatment of infections in chronic lymphoid leukemia?

### M Benlabed^1^, S Nedjari^2^, R Gaudy^3^, F Ouanes^2^, A Ladjouze^4^

#### ^1^Lille University, Anesthesiology, Lille, France; ^2^Algiers University Hospital, Anesthesiology and Intensive Care , Algiers, Algeria; ^3^Lille University, Lille, France; ^4^Algiers University Hospital, Algiers, Algeria

**Introduction:**

Hypogammaglobulinemia [1] is one of the important predisposing factor for infection in chronic lymphoid leukemia (CLL) and to prevent it, needs usually the prophylactic administration of immunoglobulins. Nevertheless, intravenous immunoglobulins (IVIg) as curative treatment [2] was never well evaluated in these patients. So the objective of our study was to analyze the mortality and the morbidity of 30 patients with CLL admitted in an university hospital ICU for severe pulmonary infections and receiving or not administration of IVIg as curative treatment.

**Methods:**

We conducted a prospective observational study from April 2018 to April 2020 and selected 30 patients with early diagnosed and untreated CLL. Patients were 75+-10 years old and were mechanically ventilated for severe pneumoccoci bilateral pulmonary infections. We divided the patients in two groups: A first group (experimental group) of 15 patients received IVIg 1g/kg associated with antibiotics on the first day of the diagnosis of bacterial pneumonia and a second group (control group) of 15 patients received placebo and antibiotics. In each group, we performed a dosage of IgG before and one week after the administration of IVIg.

**Results:**

Before IVIg, the level of IgG was in the normal range (8 g/l) in the two groups. One week after IVIg, in the experimental group, the level was significantly higher than in the control (ctrl) group (14.73+-1.16 VS 8+-1.25 g/l) p<0.0001 (Table 1). Duration of mechanical ventilation was shorter in the experimental group than in the ctrl group (10.4+-1.16 VS 17.2+-1.56 days) p<0.001. Septic shock occurred less frequently in the experimental group than in the ctrl group (5 VS 11 patients). Mortality rate was significantly lower in the experimental group than in the ctrl group. (47% VS 80%).

**Conclusions:**

A large prospective randomized clinical trial is needed to confirm the curative effect of IVIg for infections in patients with LLC.

**References**

1. Andersen M et al. Leuk Lymphoma 57:1592-9, 2016

2. Tsiodras S et al. Mayo Clin Proc 75:1039-54, 2000

**Table 1 (abstract P553).** Dosage of IgG and duration of mechanical ventilation in experimental and control group

	Experimental group	Control group	
	Mean ± SD	Mean ± SD	
Dosage IgG T7 days. g/l	14.73 ± 1.16	8.00 ± 1.25	p<0.0001
Duration mechanical ventilation/days	10.4 ± 1.40	17.2 ± 1.56	p<0.0001

## P554 The effect of plasmapheresis on mortality in patients monitored in the pediatric intensive care unit due to hemophagocytic lymphohistiocytosis: single center experience

### N Akcay, HS Kihtir, U Kocoglu Barlas, E Sevketoglu

#### University of Health Sciences, Bakirkoy Dr Sadi Konuk Training and Research Center, Pediatric Intensive Care Unit, Department of Pediatrics, Istanbul, Turkey

**Introduction:**

Hemophagocytic lymphohistiocytosis (HLH) is a life threatening condition caused by excessive stimulation of lymphocytes and histiocytes that secrete inflammatory cytokines. In this study, the effect of therapeutic plasma exchange in HLH and MAS has been observed.

**Methods:**

The patients who were diagnosed with HLH and MAS in PİCU, were included in this study. The age, sex, PRISM scores, diagnosis, the length of hospital stay, C-reactive protein and procalcitonin levels, thrombocyte counts, fibrinogen and ferritin levels, neutrophil and lymphocytes counts, hemoglobin, triglycerides, d-dimer and albumin levels and the need for therapeutic plasma exchange of the patients were noted.

**Results:**

Twenty male (74.1%), seven female (25.9%) patients were included in the study. The median age of the patients was 3.5(0.6-5.8), the length of hospital stay was 11(5.0-22.0). Genetic causes existed in 13 patients (48%), 6 (22.2%) of the patients had causes secondary to infections, 5 of them (18.5%) had rheumatologic causes, and 3 (9.3%) of them had other causes. Therapeutic plasma exchange was performed on 21 (81.5%) patients. Intravenous immunoglobulin was given to 19 (70.4%) of them. Eight (36%) patients in the plasmapheresis group and 1 (20%) patient who was not in the group died. The standard mortality rate (observed/expected mortality) of the patients who were not in the plasmapheresis group was 1.74, higher than of the ones in the plasmapheresis group (1.52). The incidence of death of the ones whose albumin levels were under 2.49 was 32.9 times higher than the ones whose levels were above 2.49 (p:0.001). There was no related mortality except for the albumin levels.

**Conclusions:**

Although there was no statistically meaningful difference in terms of mortality in between the two groups, the calculated standard mortality rate was higher in the patients who did not have plasmapheresis. These results need to be supported with wider studies since the number of cases was not enough in our study.

## P555 Up-skilling staff for re-deployment to intensive care during the COVID-19 pandemic: the role of online learning resources

### R Wong^1^, M White^1^, D Turton^1^, E Jenkins^1^, H Mills^1^, P Lee^1^, A Collini^1^, J Kobyluch^1^, E Young^1^, D Melia^2^

#### ^1^Barts Health Education Academy, London, United Kingdom; ^2^Barts Health, Dept of Anaesthesia and Critical Care, London, United Kingdom

**Introduction:**

Preparing staff for redeployment to intensive care medicine (ICM) during the COVID-19 pandemic took a multi-faceted approach in our Trust, which includes 5 separate hospitals. In addition to face-to-face training, we found that online resources were positively received by staff preparing for redeployment.

**Methods:**

An entirely new set of educational and reference resources were rapidly collated onto an existing online platform previously used for Statutory and Mandatory Training. These resources were organized into general information for all healthcare professionals (e.g. personal protective equipment and resuscitation) and profession-specific sections aimed at preparing doctors and nurses for their new roles. Within the profession-specific sections, the material was arranged according to where the staff member would be redeployed: ICM or medical wards. Efforts were made to ensure material was as accessible as possible on any device in order to optimize the user experience. As a sample, all 131 staff who accessed the resources over 3 days (18-20 April) were contacted by email to complete feedback via SurveyMonkey.

**Results:**

78% of respondents found the resources to be very or extremely useful (Figure 1) and 73% found the usability and design of the site to be very or extremely clear. 46% of respondents indicated that they were most interested in the ICM resources.

**Conclusions:**

The COVID-19 pandemic focused attention on optimizing the provision of resources to prepare staff for new roles. Positive feedback for the online resources used in our hospitals during the pandemic suggests that similar platforms could be utilized as a matter of course in the future. Doctors-in-training, in particular, rotate frequently between departments and access to online resources may aid their preparation for, and effectiveness in, new roles within ICM.

**Fig. 1 (abstract P555).**
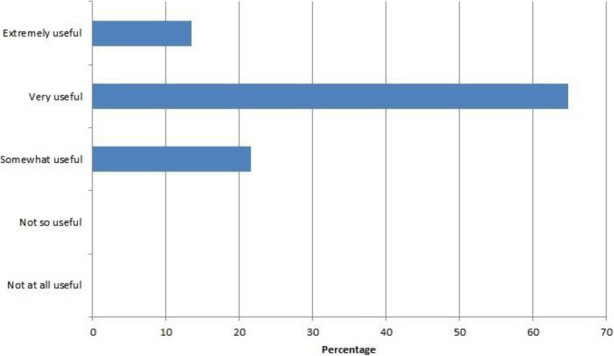
Usefulness ratings of resources

## P556 Streamlining the application of machine learning techniques to ICU practices - a preliminary study

### A Sakagianni^1^, G Feretzakis^2^, E Loupelis^3^, D Kalles^4^, M Lada^5^, M Martsoukou^6^, C Christopoulos^5^, N Skarmoutsou^6^, S Michelidou^1^, K Valakis^1^

#### ^1^Sismanoglio General Hospital, Intensive Care Unit, Athens, Greece; ^2^Sismanoglio General Hospital, IT Department, Department of Quality Control Research and Continuing Education, Athens, Greece; ^3^Sismanoglio General Hospital, IT Department, Athens, Greece; ^4^School of Science and Technology, Hellenic Open University, Patras, Greece; ^5^Sismanoglio General Hospital, Internal Medicine Department, Athens, Greece; ^6^Sismanoglio General Hospital, Microbiology Laboratory, Athens, Greece

**Introduction:**

This study aimed to evaluate the performance of a standard machine learning (ML) technique, namely a multinomial logistic regression model with a ridge estimator, on the task of predicting bacterial resistance to antibiotics in an intensive care unit (ICU). ML techniques are increasingly applied to identify resistance factors and to assist the clinician in selecting empirical antibiotic treatment.

**Methods:**

We analyzed the microbiological data collected from 345 ICU patients during two years (2017 and 2018), in a public tertiary hospital in Greece. The dataset of 23,067 instances contains the attributes of gender, age, type of the sample (e.g., blood, tracheal aspirate, urine), Gram stain, antibiotics, and the class of antimicrobial susceptibility. We have used the WEKA-Data Mining Software in Java Workbench and, specifically, a multinomial logistic regression model with a ridge estimator [1] with 10-fold cross-validation.

**Results:**

The multinomial logistic regression model with a ridge estimator weighted average results achieved an F-measure of 0.664, a Precision value of 0.671, a Recall value of 0.667, and an area under the receiver operating characteristic curve (AUC ROC) of 0.726. The experimental results, especially the value of the AUC ROC, indicate that this model could be appropriate for ICU antimicrobial susceptibility forecasting, based on similar datasets with limited attributes, as in our case. The method’s performance compares favorably with the best model, a Multilayer Perceptron (an artificial neural network), as has been reported in our previous study [2] based on the same dataset.

**Conclusions:**

A multinomial logistic regression learning classifier was validated on an ICU antimicrobial susceptibility dataset as a low-cost method to complement the information available to medical doctors before they dispense direct proper treatment.

**References**

1. Le Cessie S et al. Applied Statistics 41:191-201, 1992

2. Feretzakis G et al. Antibiotics 9:50, 2020

## P557 Does teaching ultrasound guided cannulation to junior doctors reduce requests for difficult venous access from intensive care and anesthetic departments?

### S Sahota^1^, W Carter-Esdale^2^, K Pope^1^, K Mitchell^3^

#### ^1^Treliske, Critical Care, Truro, United Kingdom; ^2^Cardiff and Vale Health Board, Psychiatry, Cardiff, United Kingdom; ^3^Treliske, Anaesthetics and Pain, Truro, United Kingdom

**Introduction:**

Peripheral intravenous cannulation is a skill that is expected of newly qualified doctors in the UK, however there is no standardized guidance on how to proceed when this is challenging. When this is the case, intensive care and anesthetic departments are often called to provide assistance. A recognized technique is using ultrasound-guidance; however, this is not routinely taught to newly qualified doctors. In our pilot study, we assessed whether first year doctors would benefit from ultrasound cannulation training as a mandatory clinical skill, and whether it affects venous access requests from intensive care and anesthetics departments.

**Methods:**

After a preliminary survey to our first year doctors, we incorporated ultrasound cannulation training into regular clinical skills sessions. The teaching sessions were a composite of both didactic and practical learning. Following the session, surveys based on the Kirkpatrick learning evaluation model were collected after teaching sessions. Follow up surveys were also collected from the same participants once the doctors had entered the next year of their training.

**Results:**

Our training sessions increased confidence with ultrasound cannulation for 88% of participants. The same number reported incorporating ultrasound cannulation into their independent practice, i.e., Kirkpatrick Level 3: change in behavior. 82% reported that this new skill reduced the amount of difficult venous access they escalated to their seniors and 71% reported that they had been able to cannulate patients where previously they would have had to request help from anesthetics or intensive care departments.

**Conclusions:**

Our pilot study concluded that first year qualified junior doctors found ultrasound training for peripheral venous access useful, relevant to their clinical practice, and reduced cannula requests from anesthetics or intensive care. Owing to the success of our pilot program, ultrasound cannulation is now a skill that we offer as standard to all our junior doctors.

## P558 Intraventricular colistin: a successful treatment of multidrug-resistant and extensively drug-resistant *Acinetobacter baumannii* meningitis

### W Sellami^1^, I Ben Mrad^2^, S Saidani^2^, H Askri^2^, Z Hajjej^2^, I Labbene^2^, M Ferjani^2^

#### ^1^Military Hospital of Tunis, Department of Anesthesiology and Intensive Care Unit, Tunis, Tunisia; ^2^Military Hospital of Tunis, Tunis, Tunisia

**Introduction:**

*Acinetobacter baumannii* (AB) appears as the most frequent Gram negative micro-organism implicated in post neurosurgical nososcomial meningitis [1]. Intraventricular colistin (IVT) as the last therapeutic resort for the treatment of multidrug-resistant (MDR) and extensively drug-resistant (XDR) *Acinetobacter baumannii* meningitis. The aim of this study was to determinate the epidemiological characteristics, outcomes end prognosis of patients complicated with XDR *Acinetobacter baumannii* meningitis.

**Methods:**

Retrospectively we included patients hospitalized for post neurosurgical nosocomial AB meningitis and treated by IVT colistin (125000 IU) in addition with IV colistin (Loading dose 9 MIU followed by 4.5 MIU every 12h) for a median duration of 21 days. For each patient, the following data were recorded retrospectively: age, sex, primary diagnosis, antimicrobial regimens prescribed, days of sterilization of cerebrospinal fluid, duration of IVT colistin treatment, toxicity, outcome and follow-up.

**Results:**

A total of 30 patients were found to have been treated with IVT colistin for AB meningitis. The mean age of patients was 54.1 years. In all cases, meningitis was secondary to neurosurgery procedures for the management of various cerebral nervous system diseases. The median time for admission to diagnosis of meningitis was 7 days. Regarding the resistance patter, 30 cases were defined XDR strains. Successful clinical and bacteriological outcome was achieved in 83% of AB meningitis cases. Five patients died of septic shock.

**Conclusions:**

IVT administration of colistin appears to be a rather safe and efficacious therapeutic option for the treatment of XDR *Acinetobacter baumannii* meningitis and should be considered an applicable procedure in the neurosurgery setting.

**Reference**

1. Giamarellou H et al. Int J Antimicrob Agents 32:106-19, 2008

## P559 Population pharmacokinetics of ceftriaxone for prophylaxis in liver transplantation

### GS Casu^1^, D Fage^2^, B Ickx^3^, V Lucidi^4^, T Gustot^5^, F Cotton^2^, F Jacobs^6^, L Van Obbergh^3^, FS Taccone^1^, M Hites^6^

#### ^1^Hôpital Erasme, Department of Intensive Care, Bruxelles, Belgium; ^2^Hôpital Erasme, Department of Clinical Chemistry, Bruxelles, Belgium; ^3^Hôpital Erasme, Department of Anesthesiology, Bruxelles, Belgium; ^4^Hôpital Erasme, Department of Digestive Surgery, Bruxelles, Belgium; ^5^Hôpital Erasme, Department of Hepatology, Bruxelles, Belgium; ^6^Hôpital Erasme, Department of Infectious Diseases, Bruxelles, Belgium

**Introduction:**

Alterations of antibiotic pharmacokinetics (PK) during liver transplantation surgery (LTS) may result in insufficient β-lactam concentrations during post-operative prophylaxis. Nevertheless, no clear guideline is provided concerning specific dosing strategies. Furthermore, the available PK data are very limited, coming from old studies, heterogeneous populations and antibiotic regimens. The objective was to assess whether our institutional prophylaxis regimen based on ceftriaxone (CFT) results in appropriate free drug concentrations of this antibiotic for LTS.

**Methods:**

All consecutive adult patients undergoing LTS were included if prophylaxis regimen was based on CFT (2 g before incision, 1 g q6h intraoperatively followed by 2 g q24h postoperatively). Total (tCFT) and free (fCFT) serum concentrations of the antibiotics were determined by ultrahigh pressure liquid chromatography on samples taken peroperatively (before and 0.5, 1.5, 3, 4.5 and 6h after antibiotic administration and at the end of surgery) and postoperatively (8 h and 16 h post-operatively and before and 0.5, 1.5, 3, 4.5 and 6h after 1^st^ postoperative CFT redosing).

**Results:**

170 blood samples were analyzed from 11 patients (2 were sampled only peroperatively). All patients had free CFT serum concentrations for 100% of the time between two doses above the minimal inhibitory concentration (100%T>MIC) corresponding to the clinical breakpoint for *Enterobacteriaceae* spp, as defined by the European Committee on Antimicrobial Susceptibility Testing (EUCAST; i.e. target MIC 2 mg/dl). CFT PK model parameters were (interindividual variability, CV%): for tCFT, clearance (CL) of 0.68 l/h (61%), V1 11 l (26%), V1_occ3_ 16 l (18.3%) and V2 10 l (50%); for fCFT, CL of 3.96 l/h (74%), V1 39.6 l (70%), V1_occ2.4_ 30.7 l (78%) and V2 46.8 l (50%). No significant difference between V1 and V1_occ2.4_ was observed. No SSIs were reported.

**Conclusions:**

Adequate free serum concentrations during the entire prophylaxis period were achieved for CFT.

## P560 Health economics of nosocomial pneumonia in UK intensive care units (ICU): an exploratory study

### A Wagner^1^, D Turner^1^, V Enne^2^, R Baldan^1^, C Russell^1^, DM Livermore^1^

#### ^1^University of East Anglia, Norwich Medical School, Norwich, United Kingdom; ^2^University College London, Centre for Clinical Microbiology, London, United Kingdom

**Introduction:**

The objective was to establish a baseline, a cross-sectional health economic survey of recent Hospital-acquired and ventilator-associated pneumonia (HAP and VAP). Both HAP and VAP cause considerable health care costs, as well as significantly impacting patient outcomes. The UK-based INHALE research program is exploring the use of rapid molecular diagnostics to improve the treatment of HAP/VAP patients by more swiftly identifying causative pathogens and their antibiotic resistances. Resulting changes to patient management and antibiotic use potentially have substantial resource implications, quantifying these is important.

**Methods:**

Patients, or their representatives, from 4 UK ICUs were approached for involvement if they were either i) starting a course of antibiotics or ii) having a change of antibiotics for the treatment of HAP or VAP. We collected information to allow estimates of: cost of ICU stay [length of stay (LOS) and related health resource group (HRG)]; acquisition cost (from the British National Formulary) of antibiotics used in the 21-days after recruitment; and quality of life (EuroQoL EQ-5D-5L) in those alive at 21-days.

**Results:**

N=143 patients were recruited. They had considerable ICU-associated LOS and hospital costs: their mean stay was 22 days and mean costs were GBP £43,100. Both LOS and costs were heavily right-skewed (most values are low but the remainder take large values). Compared with HAP, VAP caused greater LOS and ICU costs. Antibiotics themselves formed only a tiny fraction of total costs (mean 21-day cost was £321). A total of 43 people completed the EQ-5D-5L: a wide range of utilities resulted, ranging from 0.8 to -0.4 (with negatives indicating states valued worse than death).

**Conclusions:**

HAP, and particularly VAP, are associated with significant hospital costs. Interventions that could improve the care of individuals with HAP/VAP and reduce their LOS would significantly free up scarce ICU resources, allowing other patients to be treated.

## P561 Performance of a PCR based syndromic panel compared to routine culture and microscopy in patients suspected of pneumonia

### V Andrews, M Pinholt, U Schneider, L Søes, K Schønning, G Lisby

#### Department of Clinical Microbiology, Hvidovre and Amager Hospital, Hvidovre, Denmark

**Introductions:**

Syndromic testing for lower respiratory tract infections with Biofire® Filmarray® Pneumonia Panel (BF) consists of a multiplex PCR with 27 pathogens and a turn-around-time of two hours. Routine diagnostic of bacterial pneumonia in the Capital Region of Denmark consist of culture preceded by microscopy for quality assessment of sputum. Turn-around-time and sensitivity of culture can be a limiting factor for targeted antimicrobial treatment. Hence, we evaluated BF performance against culture.

**Methods:**

From January to May 2019, 298 samples were collected consecutively from hospitalized patients with suspected pneumonia. Samples were sent routinely to the Department of Clinical Microbiology for culture and additional testing by BF. Retrospectively, patients were categorized into ‘pneumonia’, ‘probable pneumonia’ and “no pneumonia”. Analytical performance was evaluated by bacterial pathogen concordance between the two methods. Clinical performance was determined regarding pneumonia/not pneumonia and detection of a positive/negative bacterial pathogen and evaluated by sensitivity, positive predictive value (PPV), negative predictive value (NPV) and efficacy.

**Results:**

98 patients had pneumonia, 71 had probable pneumonia and 129 had no pneumonia. Positive agreement between culture and BF was 42%. The rate increased to 67% when pathogens in lowest quantity in BF were excluded. Sensitivity of BF was improved from 73% to 89%, and for culture from 43% to 58%, when only high quality samples were included. For BF, PPV: 50%, NPV: 69% and efficacy: 57% were comparable to culture (PPV: 52%; NPV: 62%; efficacy: 58%); this increased slightly for both BF (PPV: 55%; NPV: 76%; efficacy: 61) and culture (PPV: 55%), when only high quality samples were included.

**Conclusions:**

PPV and NPV of both BF and culture were low and are therefore best used in patients in whom the pneumonia diagnosis has been established clinically. Indiscriminate use may be diagnostically misleading and a cause of improper use of antibiotics.

## P562 Antibiotic prescribing decisions in intensive care: a qualitative study

### AM Pandolfo^1^, R Horne^1^, Y Jani^1^, N Bidad^1^, SJ Brett^2^, TW Reader^3^, D Brealey^4^, VI Enne^5^, DM Livermore^6^, V Gant^7^

#### ^1^University College London, Department of Practice and Policy, London, United Kingdom; ^2^Imperial College Healthcare NHS Trust, Centre for Perioperative Medicine and Critical Care Research, London, United Kingdom; ^3^London School of Economics, Department of Psychological and Behavioural Science, London, United Kingdom; ^4^University College London Hospitals NHS Foundation Trust, Division of Critical Care, London, United Kingdom; ^5^University College London, Division of Infection and Immunity, London, United Kingdom; ^6^University of East Anglia, Norwich Medical School, Norwich, United Kingdom; ^7^University College London Hospitals NHS Foundation Trust, Department of Medical Microbiology, London, United Kingdom

**Introduction:**

Antimicrobial stewardship (AMS) is a key issue in ICUs; however, few studies have examined antibiotic decision-making in this context. INHALE is a research program examining molecular diagnostics’ influence on hospital-acquired pneumonia (HAP) prescribing in ICU. This study explored how prescriber perceptions and contextual factors influence ICU antibiotic decisions, before implementing molecular tests.

**Methods:**

Four focus groups and 34 interviews were conducted with clinicians in four UK ICUs. Focus groups explored perceptions of factors influencing prescribing decisions and interviews explored decision processes using clinical vignettes depicting HAP. Data were analyzed using thematic analysis.

**Results:**

Prescriber perceptions were key to decision-making. Most clinicians balanced societal risks of antimicrobial resistance (AMR) against individual patients’ needs, with the latter generally prioritized. In uncertainty, the default was to prescribe on the basis that antibiotics might prevent patient mortality, with clinicians viewing prescribing as more defensible than withholding. Antibiotic side effects were rarely mentioned. Clinicians were aware of AMR and strove to withhold potentially unnecessary antibiotics. This aim was counter-balanced by previous negative experiences, which motivated prescribing antibiotics ‘just in case’ of infection. Clinicians’ perceptions interacted with the prescribing context. Examples include a lower perceived threshold to prescribe antibiotics out of hours, input from non-ICU team members, and varied local prescribing norms.

**Conclusions:**

When making prescribing decisions, clinicians’ understandable fear of undertreating possible infection often conflicts with AMS aspirations. Prescribers seem to be driven by perceived negative consequences for patients and themselves over more distal issues of AMR. Rapid evidence-based support from more effective diagnostics may help reconcile these competing priorities.

## P563 Biofire® FilmArray® pneumonia panel in the evaluation of severe lower respiratory tract infections

### E Kyriazopoulou^1^, A Karageorgos^2^, L Liaskou-Antoniou^2^, P Koufargyris^2^, A Safarika^2^, G Adamis^3^, A Antoniadou^2^, EJ Giamarellos-Bourboulis^2^

#### ^1^National Kapodistrian University of Athens, 4th Department of Internal Medicine, Athens, Greece; ^2^National Kapodistrian University of Athens, Athens, Greece; ^3^General Hospital of Athens "Georgios Gennimatas", 1st Department of Internal Medicine, Athens, Greece

**Introduction:**

Although viruses are considered the commonest cause of admitted cases of lower respiratory tract infections (LRΤΙs) [1] data for patients with severe LRTI are missing. This study aimed to define the epidemiology of severe LRTI using the Biofire® FilmArray® Pneumonia *plus* (PN*plus*) Panel.

**Methods:**

This was a sub-study of the PROGRESS trial where procalcitonin (PCT)-guidance for early stop of antibiotics was used to prevent infection-associated adverse events in patients with sepsis (ClinicalTrials.gov NCT03333304). PN*plus* Panel was performed retrospectively in frozen lower respiratory samples of 90 septic patients (Sepsis-3) with LRTI. Primary endpoint was the comparison of the detection rate of pathogens between conventional microbiology (blood, sputum, pleural fluid cultures and urine antigen detection) and PN*plus* Panel. Secondary endpoints were the association with inflammatory host response and detection of antibiotic resistance.

**Results:**

56 patients with community-acquired (CAP) and 34 with healthcare-associated pneumonia (HCAP) were studied; median pneumonia severity index was 113 (88-135). PN*plus* detected at least one pathogen in 65 patients (72.2%) compared to 10% detected by conventional microbiology (p<0.0001); bacteria were the commonest pathogens (Figure 1). Median PCT was 0.49 ng/ml among patients with ≥105 copies/ml of a bacterial pathogen compared to 0.18 ng/ml in detection at lower loads (p=0.004). SOFA score, serum CRP and white blood cells did not differ in patients with undetected, bacterial, viral or mixed cause of infection. Median PCT was 0.52 ng/ml among patients with bacterial pathogens compared to 0.19 ng/ml with viral (p: 0.045). At least one resistance gene was detected in 14.4% of samples, being more common in HCAP versus CAP (32.2% vs 5.1%; p: 0.001).

**Conclusions:**

PN*plus* detects severe pneumonia pathogens at significantly greater rate than conventional microbiology and higher circulating PCT levels reflect their true virulence.

**Reference**

1. Jain S et al. N Engl J Med 373:415-27, 2015

**Fig. 1 (abstract P563).**
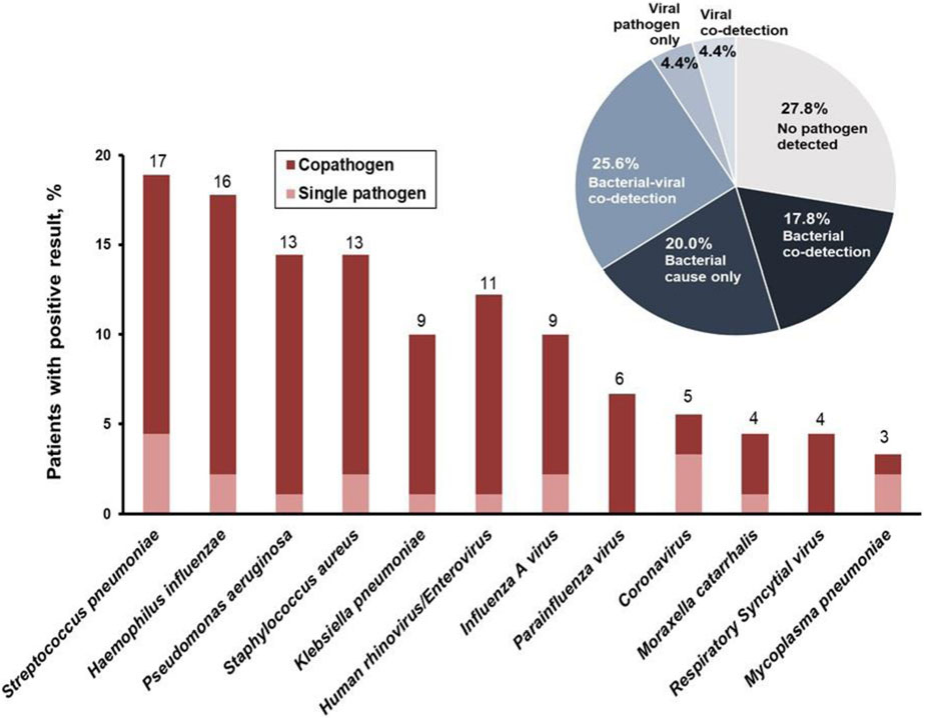
Pathogens detected by Biofire® FilmArray® Pneumonia Panel in patients with sepsis due to lower respiratory tract infections

## P564 Development and external validation of an online clinical prediction model for augmented renal clearance in adult mixed critically ill patients: the ARC predictor

### MG Gijsen^1^, CY Huang^2^, M Fléchet^2^, R Van Daele^1^, P Declercq^1^, Y Debaveye^2^, P Meersseman^2^, G Meyfroidt^2^, J Wauters^2^, I Spriet^1^

#### ^1^UZ Leuven, Pharmacy Department, Leuven, Belgium; ^2^UZ Leuven, Clinical Division and Laboratory of Intensive Care Medicine, Leuven, Belgium

**Introduction:**

Augmented renal clearance (ARC) might lead to subtherapeutic plasma levels of drugs with predominant renal clearance. Early identification of ARC remains challenging for the intensive care unit (ICU) physician. We developed and validated the ARC predictor, a clinical prediction model for ARC on the next day during ICU stay, and made it available via an online calculator. Its predictive performance was compared with that of two existing models for ARC, i.e. the ARC score and the ARCTIC score.

**Methods:**

A large multicenter database including medical, surgical and cardiac surgery ICU patients (n = 33258 ICU days) from three Belgian tertiary care academic hospitals was used for the development of the prediction model. Development was based on clinical information available during ICU stay. We assessed performance by measuring discrimination, calibration and net benefit. The final model was externally validated (n = 10259 ICU days) in a single-center population.

**Results:**

ARC was found on 19.6% of all ICU days in the development cohort. Six clinical variables were retained in the ARC predictor: day from ICU admission, age, sex, serum creatinine, trauma and cardiac surgery. External validation confirmed good performance with an area under the curve of 0.88 (95% CI 0.87 – 0.88), and a sensitivity and specificity of 84.1 (95% CI 82.5 – 85.7) and 76.3 (95% CI 75.4 – 77.2) at the default threshold probability of 0.2, respectively.

**Conclusions:**

ARC on the next day can be predicted with good performance during ICU stay, using routinely collected clinical information that is readily available at bedside. The ARC predictor is available at www.arcpredictor.com.

## P565 The INHALE trial: designing a prescribing algorithm to aid antibiotic choices for the FilmArray Pneumonia Panel plus

### Z Dhesi^1^, VI Enne^1^, V Gant^2^, DM Livermore^3^

#### ^1^University College London, Dept for Clinical Microbiology, London, United Kingdom; ^2^University College London Hospitals NHS Trust, Dept of Infection, London, United Kingdom; ^3^University of East Anglia, Microbiology, Norwich, United Kingdom

**Introduction:**

The NIHR-funded INHALE Programme aims to improve antimicrobial stewardship by using molecular microbiology diagnostics for HAP/VAP in ICUs. The BioFire FilmArray Pneumonia Panel is deployed at point of care in 12 UK ICUs, with patients randomized to FilmArray-guided management or to standard empiric antimicrobials and laboratory microbiology testing. The FilmArray seeks 34 organism and gene targets, with results in 1.5 h; we designed and field-tested an algorithm to guide antimicrobial prescribing based on its findings.

**Methods:**

The algorithm is based on (i) the organism and resistance gene targets detected, (ii) national resistance prevalence data and (iii) the patient’s allergy status. Narrow-spectrum agents are preferred, and good stewardship encouraged. Microbiologists, ICU pharmacists, and ICU clinicians were consulted and local adaptation allowed.

**Results:**

When single organisms are found, the algorithm favors, e.g. temocillin vs. *Enterobacterales*, flucloxacillin vs. MSSA and co-amoxiclav vs. *H. influenzae*; discontinuation is advised if no organism is found and the patient lacks evidence of infection; broader spectrum agents are favored for combinations of organisms. Among 10 adult sites, 4 adopted the algorithm unaltered and 2 with minor variation. Concerns were: unwillingness to adopt: (i) temocillin for *Enterobacterales*; (ii) ceftazidime vs. *Pseudomonas*; or (iii) cephalosporins for patients with mild β-lactam allergy. Greater variation was needed at 2 pediatric ICUs. There was debate about infection control implications of rapid ICU-based tests.

**Conclusions:**

The algorithm aims to ensure that rapid microbiology translates to optimized therapy. It was not possible to impose a single algorithm at all ICUs, but core elements and principles were retained. An early audit of RCT results indicates most test-arm treatments are being guided by the algorithm, illustrating the approach’s potential.

## P566 Real-life use of intravenous (i.v.) fosfomycin in patients with infective endocarditis – insights from the FORTRESS study

### S Hagel^1^, S Kluge^2^, S Lindau^3^, FA Litty^4^, KF Bodmann^5^

#### ^1^Universitätsklinikum Jena, Jena, Germany; ^2^Universitätsklinikum Hamburg-Eppendorf, Hamburg, Germany; ^3^Universitätsklinikum Frankfurt, Frankfurt, Germany; ^4^InfectoPharm Arzneimittel und Consilium GmbH , Medical Scientific Department, Heppenheim, Germany; ^5^Kliniken Nordoberpfalz, Weiden, Germany

**Introduction:**

Here, we present first interim results on the real-life use of i.v. fosfomycin in a subgroup of critically ill patients with IE.

**Methods:**

Prospective, non-interventional and monitored European multicenter study (FORTRESS; NCT02979951). The primary objective is clinical success, defined as clinical cure or improvement incl. microbiological cure at end of fosfomycin treatment (EOT). Secondary objectives are microbiological cure, clinical evaluations at different time points, and safety.

**Results:**

Currently (01/2020), 245 patients with severe infections have been enrolled, thereof 14 patients with IE (3 female, 11 male, mean age 64y). Thirteen patients with IE (93%) were treated in intensive care and 7 (50%) had sepsis or septic shock at baseline. Mean APACHE II score at baseline was 21. Seven (50%) patients had a prosthetic valve associated IE, one (7%) had a cardiac device associated IE, and two (14%) had both. Eleven (79%) IE were left-sided, one (7%) right-sided, and two (14%) were both-sided. Five patients had concomitant severe embolic complications, thereof two patients with stroke. Imaging results showed vegetation in 13 (93%) cases and abscess formation in one (7%) case. Twelve (86%) cases of IE were microbiologically confirmed. Causative pathogens were mostly staphylococci (12/14 (86%) patients), particularly MSSA (6/14 (43%) patients) and CoNS (4/14 (29%) patients). I.v. fosfomycin was used with a daily dose of 15 g/day (median) for a mean duration of 17 days and in combination therapy, particularly with beta-lactams, vancomycin or daptomycin. Clinical success was reported in 11/14 (79%) patients, thereof in 4/4 patients without foreign body involvement and in 7/10 (70%) patients with foreign body associated IE. All 14 patients were microbiologically cured at EOT. Four (29%) patients had adverse drug reactions.

**Conclusions:**

These new insights from daily clinical practice suggest that i.v. fosfomycin is a valuable combination partner for the treatment of IE even in cases of foreign body involvement.

## P567 Intravenous fosfomycin in challenging cases of complicated skin and soft tissue infections (cSSTI)

### S Kluge^1^, K Schmidt^2^, S Lindau^3^, M Zoller^4^, J Kielstein^5^, V Leshchinskiy^6^, D Kindgen-Milles^7^, S Hagel^8^, FA Litty^9^, KF Bodmann^10^

#### ^1^Universitätsklinikum Hamburg-Eppendorf, Hamburg, Germany; ^2^Charité - Universitätsmedizin Berlin, Berlin, Germany; ^3^Universitätsklinikum Frankfurt, Frankfurt, Germany; ^4^LMU - Klinikum der Universität München, Munich, Germany; ^5^Städtisches Klinikum Braunschweig gGmbH, Braunschweig, Germany; ^6^Universitätsklinikum Schleswig-Holstein, Lübeck, Germany; ^7^Universitätsklinikum Düsseldorf, Düsseldorf, Germany; ^8^Universitätsklinikum Jena, Jena, Germany; ^9^InfectoPharm Arzneimittel und Consilium GmbH, Medical Scientific Department, Heppenheim, Germany; ^10^Kliniken Nordoberpfalz, Weiden, Germany

**Introduction:**

Here, we present first real-life experience with i.v. fosfomycin in a subgroup of patients with cSSTI from an ongoing international study.

**Methods:**

Prospective, non-interventional and monitored European multicenter study (FORTRESS; NCT02979951). The primary objective is clinical success, defined as clinical cure or improvement incl. microbiological cure at end of fosfomycin treatment. cSSTI was defined according to common definitions (US, EU).

**Results:**

Twenty-four of 245 currently (Jan 20) enrolled patients with severe infections had cSSTI (11 female, 13 male; mean age 58y), of which 17 (71%) were treated in intensive care. Fourteen (58%) patients had at least one additional risk factor for cSSTI. Ten (42%) patients had sepsis or septic shock at baseline. Fourteen patients (58%) had surgical site infections, 18 (75%) non-necrotizing cSSTI, thereof 14 (78%) with abscess formation, and four (17%) necrotizing cSSTI (fasciitis, cellulitis/severe phlegmon, abscess). Twenty cases (83%) of cSSTI were considered as acute and four (17%) as chronic infections. Eighteen (75%) infections were microbiologically confirmed. Causative pathogens were mostly staphylococci (15/24 patients; 63%), particularly methicillin-sensitive *S. aureus* (n=10/24 patients; 42%), *E. faecium* (1/24 patients; 4%), streptococci (n=5/24 patients; 21%), and Gram-negative species (n=12/24 patients; 50%). I.v. fosfomycin was used in a dose of 14 g/day (median) for a mean duration of 20 days, often in combination with carbapenems, penicillins or cephalosporins. Clinical success was reported in 19/24 (79%) patients, in 12/15 (80%) patients with abscess involvement, and in 8/10 (80%) patients with concomitant sepsis or septic shock. Fifteen adverse events were considered being related to fosfomycin treatment.

**Conclusions:**

These interim data from clinical practice emphasize that i.v. fosfomycin is a useful combination partner for treatment of cSSTI even in life-threatening cases.

## P568 Interleukin-6 and interleukin-10 as predictors of poor clinical outcome in major trauma

### M Jones^1^, J Hanison^2^, R Appreteusi^1^, B Allarakia^1^, D Ramaswamy^1^, S Namvar^1^, D Horner^3^, R Body^4^, K Mackway-Jones^4^, N Nirmalan^1^

#### ^1^Engineering, School of Science, University of Salford, Manchester, United Kingdom; ^2^Critical Care Unit, Manchester Royal Infirmary, Manchester, United Kingdom; ^3^Critical Care, Salford Royal NHS Foundation Trust, Manchester, United Kingdom; ^4^Emergency Medicine, Manchester Royal Infirmary, Manchester, United Kingdom

**Introduction:**

The SOFA score is a reliable tool to describe organ dysfunction following trauma [1]. Serum IL-6 and IL-10 have been shown to rise early following injury and potentially be useful markers in predicting clinical outcomes [2]. This study evaluated the predictive potential of early IL-6 and IL-10 levels on SOFA outcome in patients following major trauma.

**Methods:**

One hundred major trauma patients were included in this study. Inclusion criteria were 1) injury severity requiring immediate transfer from the emergency department to the operating theatre or critical care, 2) enrolment within 24 hours of the injury. Principle exclusion criteria were 1) age <18 years, 2) patients on steroids or other immunosuppressive medication. Serum samples collected day 1 and day 3 were evaluated for IL-6 and IL-10 using cytometric bead arrays. SOFA scores were calculated on day 1 and day 5, from which delta SOFA was calculated. All study procedures were approved by the National Research Ethics Committee South Manchester.

**Results:**

Day 1 IL-6 did not correlate with day 5 SOFA (r = 0.120, p = 0.21 n = 100), day 3 IL-6 correlated with day 5 SOFA (r = 0.40, p = 0.002 n = 52). The day 1 (r = 0.24, p = 0.018 n = 100,) and day 3 (r = 0.43, p = 0.0013, n = 21,) IL-10 concentrations, however, were both found to significantly correlate with patients’ day 5 SOFA scores. Patients with a delta SOFA of < 0, indicating recovery, display a lower IL6:IL10 ratio on both day 1 serum (7.94 ±0.81 compared to 14.15±2.30, p = 0.03) and day 3 serum (5.11 ±0.78 compared to 10.57±1.32, p = 0.0008) (Figure 1).

**Conclusions:**

IL-10 may be a useful biomarker to predict late organ dysfunction following major trauma. Furthermore, the ratio of IL6:IL10 on both day 1 and day 3 post injury, may be of particular value in predicting the risk of deterioration or improvement (delta SOFA) in the first 5 days after trauma.

**References**

1. Antonelli M et al. Intensive Care Med 25:389-94, 1999.

2. Stensballe J et al. Acta Anaesthesiol Scand 53:515-21, 2009.

**Fig. 1 (abstract P568).**
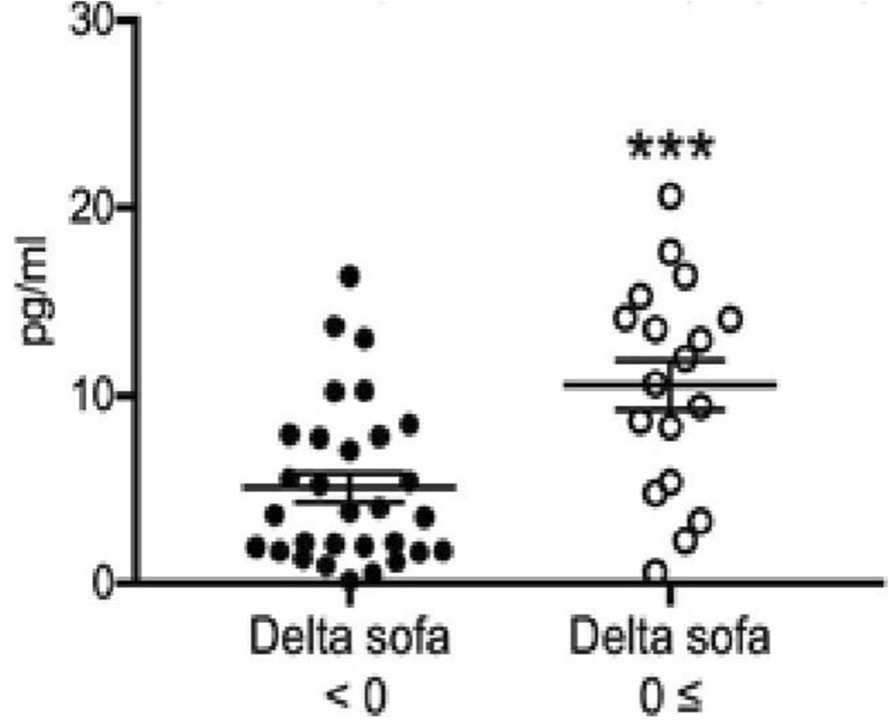
Day 3 IL6:IL10 ratio grouped by delta SOFA Day 1 to Day 5

## P569 Mild increases of creatinine after moderate and high-risk abdominal surgery are associated with long-term renal injury: a retrospective cohort study

### Z Mokhtari^1^, L Van Obbergh^1^, J Rinehart^2^, P Van der Linden^3^, A Joosten^1^

#### ^1^Department of Anesthesiology, Erasme University Hospital, Brussels, Belgium; ^2^Anesthesiology, UCI, Irvine, United States; ^3^CHU Brugmann, Anesthesiology, Brussels, Belgium

**Introduction:**

The impact of mild acute kidney injury (AKI) observed in the immediate postoperative period after major surgery on long term renal function remains poorly studied. According to the “Kidney Disease: Improving Global Outcomes” (KDIGO) classification, a mild injury corresponds to a KIDIGO stage 1, characterized by an increase in creatinine of at least 0.3 mg/dl or 1.5 to 1.9 times the baseline level. We tested the hypothesis that patients who underwent moderate-to high-risk abdominal surgery and developed mild AKI in the following days would be at an increased risk of long-term renal injury compared to patients with no postoperative AKI.

**Methods:**

This single center retrospective cohort study analyzed all consecutive adult patients without chronic kidney disease who underwent elective moderate to high-risk abdominal surgery at Erasme Hospital between 2014 and 2019 and who had three relevant creatinine measurements: before surgery, during the first seven postoperative days, and long-term (1 year). The study population was divided into three groups according to the postoperative renal function: no renal injury, mild AKI (KIDIGO stage 1) and moderate to severe AKI (KIDIGO stages 2 and 3).

**Results:**

A total of 815 patients were analyzed. Overall, postoperative AKI (stage 1 to 3) incidence was 13.4% (10% with a mild AKI and 3.4% with a moderate to severe AKI). The median long-term follow-up was 360, 354 and 353 days for the three groups respectively (p=0.190). Compared to patients without postoperative AKI, those developing mild AKI had a higher risk of long-term renal injury (odds ratio [95%CI] of 3.1 [1.7-5.5]; p=0.0001). This effect is even stronger when accounting for other perioperative covariates (adjusted odds ratio [95%CI] of 4.5 [1.8-11.4]; p=0.0001).

**Conclusions:**

Patients undergoing moderate to high-risk abdominal surgery who develop a mild postoperative AKI more than tripled the odds of having renal injury one year after surgery compared with patients who did not develop any postoperative AKI.

## P570 Prediction of cardiac surgery associated-acute kidney injury (CSA-AKI) by healthcare providers, AKI predicting score systems and cell cycle arrest biomarkers. An interim analysis of PREDICTAKI, a prospective observational trial.

### L Van Laethem, W Vandenberghe, E Hoste

#### UZ Gent, ICU, Gent, Belgium

**Introductions:**

Cardiac surgery associated acute kidney injury (CSA-AKI) is a frequent complication and associated with increased morbidity and mortality. The aim of this study is to investigate whether healthcare providers (HCP) can predict CSA-AKI. The adequacy of prediction will be compared with 4 existing AKI prediction score systems (AKIpredictor score, Ng score, Crate score and Murphy score) and cell cycle arrest biomarkers (NephroCheck®).

**Methods:**

This is an interim analysis of a single center prospective study. Patients who underwent elective cardiac surgery with cardiopulmonary bypass (CPB) and without end stage kidney disease or AKI were considered for enrolment. Four hours after CPB was ceased, 3 types of HCP (ICU specialist, ICU nurse, nephrologist) were asked to predict occurrence of AKI stage 2-3, defined according to the KDIGO definition and staging system, and the need for renal replacement therapy (RRT) within 48 hours after admission on ICU, using a structured questionnaire. At the same time we measured cell cycle arrest biomarkers with the Nephrocheck® test. After analysis of the Nephrocheck® the result was communicated to the HCP and the same questionnaire was completed again. The adequacy of prediction by HCP was compared with the Nephrocheck® test results and 4 AKI predicting scoring systems by calculating the area under the ROC curve (AUC).

**Results:**

Fifty patients were included. AKI stage 2-3 occurred in 19 patients, no patient was treated with RRT. AUC was highest for the Ng, AKIpredictor score, and ICU specialist. We found that the Crate score, the NephroCheck® and nephrologist had poor performance in prediction of CSA-AKI stage 2-3 (Table 1). Providing the NephroCheck® test results did not improve the ability of AKI prediction.

**Conclusions:**

ICU specialists, the Ng and AKIpredictor scores had good performance for prediction of CSA-AKI stage 2-3 occurrence within 48-h. Providing info of the NephroCheck® test did not improve this predictive performance.

**Table 1 (abstract P570).** AUC for the 4 scoring systems (AKIpredictor score, Ng score, Crate score and Murphy score), healthcare providers and NephroCheck. CI, confidence interval; AUC, area under the curve; HCP, healthcare providers

Variable	AUC	95% CI	p-value
HCP	0.799	0.662 - 0.899	
NephroCheck®	0.584	0.436 - 0.722	0.2800
HCP and NephroCheck®	0.696	0.549 - 0.818	0.3886
Ng-score	0.910	0.795 - 0.973	0.4618
Crate-score	0.606	0.458 - 0.741	0.1835
Murphy-score	0.736	0.593 - 0.851	0.7142
AKIpredictor-score	0.897	0.778 - 0.965	0.1746

## P571 Critically ill patients with H1N1 pneumonia: two year experience in a tertiary level Indian ICU

### P Sathe, AK Parathody, R Borse

#### Department of Critical Care, Ruby Hall Clinic, Pune, India

**Introduction:**

Pandemic H1N1 viral infection is an important issue in India as in the world over. Maharashtra in particular bears a significant brunt of illness. We present the experience of treating H1N1 cases over the period of two calendar years in a 34 bed multi-disciplinary ICU in a tertiary level health care facility in Pune, Maharashtra, India. Our objective was to learn about the clinical profile, outcome and quality of life and factors influencing these, in critically ill patients with H1N1 pneumonia.

**Methods:**

Retrospective analysis of case files and phone interview of 88 patients with confirmed H1N1 pneumonia.

**Results:**

Out of 88 patients, 51 were males. Mean age was 48.23(+/-13.03). 39(44.31%) were in the 31-50 years age group and 37(42.04%) were in the 51-70 age group. Diabetes (n=16) and Hypertension (n=20) were the most common comorbidities. Majority of the patients presented with cough (n=87), breathlessness (n=85) and fever (n=84). 43 patients had severe ARDS on admission. Mean APACHE II score was 9.6(+/-5.4) Mean SOFA scores 4.99(+/-2.6). Mean Murray score was 2.37(+/-0.76). 52% (n=46) in patients who survived. Factors associated with mortality were APACHE score (p=0.00), SOFA score (p=0.00) Murray score, severe ARDS (p=0.00), requirement of vasopressor support (p=0.00) or renal replacement therapy (p=0.00) and incidence of VAP (p=0.039). Diabetes had a protective effect (p=0.04), as had non-invasive ventilation (p=0.00). Murray score (p=0.000, SOFA score (p=0.036), initiation of mechanical ventilation (p=0.003) and incidence of VAP (p=0.00) was associated with increased length of stay among the survivors.

**Conclusions:**

Higher lung specific severity scores, severe ARDS, secondary organ failure and VAP were associated with increased mortality. Among survivors, higher Murray and SOFA scores, mechanical ventilation and vasopressor use entailed a longer ICU stay.

## P572 ARDS by influenza H1N1: retrospective analysis after 10 years of experience

### JF Martínez Carmona, P Benitez, C Joya Montosa, C Aragón González, G Quesada García

#### Intensive Care Unit, Hospital Regional Universitario de Málaga, Málaga, Spain

**Introduction:**

One of the great epidemics of our time has been Influenza A (Influenza H1N1), with a great repercussion due to the associated morbidity and mortality. One of the most common forms of presentation is moderate-severe ARDS that requires prolonged admission to the ICU.

**Methods:**

Retrospective analysis of patients admitted to the ICU with H1N1 influenza disease from 2009 to 2019. The collected variables were sex, age, APACHE II, SOFA, comorbidity, need for NIMV, time of MV, complications, length of stay and mortality.

**Results:**

The sample includes 78 patients, 53.8% of them were women. The mean age was 47 years +/- 16.77. Regarding the previous pathology, the following stand out: 41% COPD, 33.3% BPH, 32.1% Obesity, 21.3% Heart disease and Dyslipidemia. APACHE II upon admission was 14 +/- 7.23 and average SOFA at admission: 5.5 +/- 3.83. The 66.7% of the patients received previous NIMV, and 74.4% required MV; been mean duration 15 days +/- 14.68. Most of them required pulmonary recruitment maneuvers; prone position was started in 35.9%. 50% of the patients presented associated renal failure, requiring RRT in 21.8% of the cases. 39.7% required a tracheostomy due to weaning. Analyzing risk factors, the following stand out: Alcoholism (OR 9,025 p 0.042 IC 1,079 - 75.51) malignancy (OR 41.45 p 0.001 IC 4.47 - 384,437), autoimmune disease (OR 7.3 p 0.042 IC 1,075 - 49.65). The medium/average stay was 13 days +/- 18.47. ICU mortality was 29.5% and hospital mortality was 33.33%. When the sample was divided into two groups, survivors and non-survivors, it was observed that the deceased patients were older (Median 46 years vs. 53 years), and presented greater renal failure (38.18% vs. 78.26%) with a greater need for RRT (28.57% vs. 61.11 %) as well as greater severity in the Berlin criteria (severe ARDS 38.18% vs. 78.26%).

**Conclusions:**

ARDS due to H1N1 influenza continues to be a relevant pathology in ICUs, with respiratory failure being the main reason for admission, with associated high morbidity and mortality.

## P573 Modulation of the receptor for advanced glycation end-products affects lung epithelial wound healing, proliferation, migration, and differentiation: an in vitro study

### R Blondonnet^1^, R Zhai^2^, E Ebrahimi^1^, C Belville^2^, L Blanchon^2^, J Audard^1^, JM Constantin^3^, V Sapin^4^, M Jabaudon^5^

#### ^1^Department of Perioperative Medicine, CHU Clermont-Ferrand, GReD, CNRS, INSERM, Clermont-Ferrand, France; ^2^GReD, CNRS, INSERM, Clermont-Ferrand, France; ^3^Sorbonne University, GRC 29, AP-HP, DMU DREAM, Department of Anesthesiology and Critical Care, Pitié-Salpêtrière Hospital, Paris, France; ^4^Department of Medical Biochemistry and Molecular Genetics, CNRS, INSERM, GReD,, Clermont-Ferrand, France; ^5^Department of Medicine, Division of Allergy, Pulmonary, and Critical Care Medicine, Vanderbilt University Medical Center, Nashville, TN, United States

**Introduction:**

The receptor for advanced glycation end-products (RAGE) is highly expressed in lung alveolar type (AT)-I cells where it can bind multiple ligands. Whether RAGE plays a role in lung alveolar epithelial repair following injury remains unknown. Here, we investigated whether RAGE modulation has an impact on lung alveolar epithelial repair and examined the influence of RAGE on the proliferation, migration, and differentiation of AT-II cells into AT-I cells.

**Methods:**

A549 cells were grown in culture to confluence and treated with RAGE agonists HMGB1 and AGEs, alone or combined with RAGE antagonist peptide. Lung epithelial repair, cell migration and cell proliferation were studied. Immunostaining of caveolin 1 and surfactant protein-C was used to investigate the expression of markers of AT-I and AT-II cells, respectively.

**Results:**

HMGB1 promoted healing of the A549 cell monolayer compared to the control condition at 24h (p=0.04) and 48h (p <0.0001), and treatment with RAP reduced this effect. AGEs stimulated wound repair at 24h (p=0.005) and 48h (p=0.0001); RAP suppressed the positive effect of AGEs at all time points. A549 cells treated with HMGB1 had higher migration capacity at 12h (p=0.0015), but not at 48h (p=0.7), and co-treatment with RAP had no influence. Treatment with AGEs decreased cell migration at 12h (p=0.002) but increased it at 48h (p=0.004); RAP inhibited this effect at 48h (p=0.005). HMGB1 increased cell proliferation at 12h (p = 0.0001) and 48h (p = 0.0003), and this effect was reversed by RAP treatment; the effects of AGEs on cell proliferation followed exactly the same patterns. Immunostaining of caveolin 1 seemed more important after treatment with HMGB1 and AGEs.

**Conclusions:**

Our results suggest that RAGE ligation may impact wound repair of lung alveolar epithelial A549 cells through multiple mechanisms including cell proliferation and cell differentiation.

## P574 Derivation of "specific population who could benefit from rosuvastatin": a secondary analysis on randomized controlled trial to uncover novel value of rosuvastatin for precise treatment of ARDS

### S Zhang, H Qiu

#### Department of Critical Care Medicine, Zhongda Hospital, School of Medicine, Southeast University, Nanjing, China

**Introduction:**

The high heterogeneity of ARDS contributes to paradoxical conclusions from previous investigations of rosuvastatin for ARDS. Identification of the population (phenotype) who could benefit from rosuvastatin is a novel exploration for precise treatment of ARDS.

**Methods:**

The patient population for this analysis consisted of unique patients with ARDS enrolled in the SAILS trial (rosuvastatin vs. placebo). Phenotypes were derived using consensus k means clustering applied to routinely available clinical variables within 6 hours of hospital presentation before receiving placebo or rosuvastatin. Kaplan–Meier statistic was used to estimate the 90 day cumulative mortality for screening specific population who could benefit from rosuvastatin, with cut-off value as p<0.05.

**Results:**

The derivation cohort included 585 patients with ARDS. Of the 4 derived phenotypes, phenotype 3 was identified as "specific population who could benefit from rosuvastatin" since rosuvastatin resulted in a significant reduction in 90 day cumulative mortality for ARDS (hazard ratio [HR] 0.29 [95% CI 0.09, 0.93]; p=0.027) (Figure 1). Meanwhile, there were no significant differences in baseline characteristics between those assigned to rosuvastatin and those assigned to placebo. Additionally, rosuvastatin markedly improved the free of cardiovascular failure (10.08±3.79 in rosuvastatin group vs 7.31±4.94 in placebo group, p=0.01) and coagulation abnormality (13.65±1.33 vs 12.15±3.77, p=0.02) to day 14 in phenotype 3. Patients classified as phenotype 3 exhibited but not limited to the relative higher platelet count (390.05±79.43 ×10^9^/l), lower CRP (20.23±11.99 μg/l) and creat (1.42±1.08 mg/dl), compared with patients classified as other phenotypes.

**Conclusions:**

This secondary analysis of SAILS trial identified the specific population who can benefit from rosuvastatin using machine learning applied to clinical variables at the time of hospital presentation, which uncovered a novel value of rosuvastatin for the treatment of ARDS.

**Fig. 1 (abstract P574).**
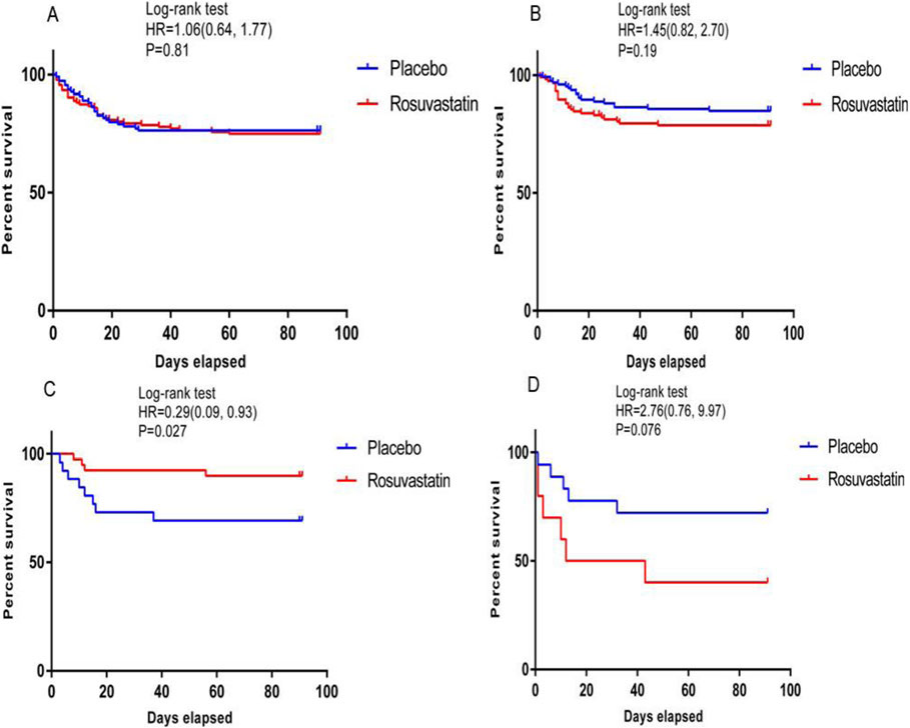
Kaplan–Meier survival curves of 90 day cumulative mortality 4 phenotypes between patients receiving Rosuvastatin and patients receiving placebo

## P575 Estimates of mechanical power directed to the respiratory system in patients with the acute respiratory distress syndrome: a secondary analysis of data from the EPVent study

### MS Schaefer^1^, SH Loring^1^, D Talmor^1^, EN Baedorf Kassis^2^

#### ^1^Department of Anesthesia, Critical Care & Pain Medicine, Beth Israel Deaconess Medical Center, Boston, MA, United States; ^2^Pulmonary, Critical Care & Sleep Medicine, Beth Israel Deaconess Medical Center, Boston, MA, United States

**Introduction:**

The acute respiratory distress syndrome (ARDS) can be aggravated by high cycling rates and airway pressures, leading to the application of mechanical power to the respiratory system. Previous estimates [1] of power include energy dissipated into the chest wall, which may not be causal to lung injury, and also include static work from positive end-expiratory pressure (PEEP). We propose and compare three additional estimates of mechanical power based on data from the EPVent study [2].

**Methods:**

Mechanical power was calculated as product of respiratory rate and inspiratory work (the sum of inspiratory pressure*∆volume products): (1) respiratory system-directed power, based on airway pressure (Pao), including static work from PEEP; (2) respiratory system-directed driving power, based on Pao-PEEP, excluding static work; (3) lung-directed power, based on transpulmonary pressure (PL) and (4) lung-directed driving power, based on ∆PL from end-expiration. We hypothesized that these estimates differ at baseline and that they are affected by PEEP titration to achieve positive PL in the EPVent study.

**Results:**

5,762 breathing cycles in 53 patients (54 [37;70] years, 38% female, BMI 27.4 [24.1;34.7] were analyzed. Respiratory system-directed power yielded the highest estimate (Table 1, p<0.001 against the other estimates) followed by respiratory system-directed driving power. Lung-directed and lung-directed driving power yielded the lowest estimates (Table 1). PL-guided PEEP titration in the EPVent intervention group differentially affected the four estimates of power (Table 1): respiratory system-directed and lung-directed power increased, while respiratory system-directed driving power and lung-directed driving power did not change (p for interaction 0.008).

**Conclusions:**

Proposed estimates of mechanical power differ based on whether static work from PEEP and chest-wall directed energy are included and they are differentially affected by changes in PL and PEEP.

**References**

1 Gattinoni L et al. Intensive Care Med 42:1567-75, 2016

2 Talmor D et al. N Engl J Med 359:2095-104, 2008

**Table 1:** **(abstract P575).** Ventilatory parameters and mechanical power at baseline and changes after PEEP titration to achieve positive transpulmonary pressure. Median [IQR]. IBW: ideal body weight; RS: respiratory system

	Baseline, n=53	∆ from baseline after PEEP titration, n=21	p value
PEEP, cmH_2_O	13.1 [10.6; 16.6]	+7.4 [0.0; 10.3]	<0.001
Tidal volume, ml/kg IBW	6.7 [5.8; 7.9]	-0.3 [-0.8; 0.6]	0.07
Respiratory rate, 1/min	25.4 [21.7; 29.0]	-0.8 [-1.8; 1.5]	0.12
RS-directed power, J/min	37.2 [24.1;51.4]	+3.6 [-1.2; 11.0]	0.033
RS-directed driving power, J/min	14.2 [11.0; 19.8]	-1.3 [-4.2; +0.8]	0.16
Lung-directed power, J/min	13.5 [8.6; 23.7]	+4.8 [0.2; 7.6]	0.021
Lung-directed driving power, J/min	13.4 [9.9; 18.8]	-1.5 [-6.3; 3.4]	0.31

## P576 Validation of a recruitability index measured by mechanical ventilator

### G Alcala^1^, S Gomes^2^, C Lima^2^, R Santiago^3^, C Kajiyama^2^, T Milá^2^, M Amato^2^

#### ^1^Faculdade de Medicina da Universidade de São Paulo, Pulmonary Division, Heart Institute (INCOR), Sao Paulo, Brazil; ^2^Faculdade de Medicina da Universidade de São Paulo, Sao Paulo, Brazil; ^3^Department of Anesthesia, Critical Care and Pain Medicine, Massachusetts General Hospital, Boston, MA, United States

**Introduction:**

Bedside assessment of lung recruitability is essential to screen ARDS patients.to improve lung function and to guide the treatment. A high recruitability index, for instance, would favor the balance towards a recruitment maneuver, followed by individualized PEEP. The purpose of this study was to measure the gain of respiratory system compliance after a brief and "light" maneuver of PEEP challenge. The goal of the maneuver was to predict a meaningful reduction in DP.

**Methods:**

The “light” recruitability maneuver was preformed through a tool developed by Nihon Koden Mechanical Ventilator (NKV550, California, EUA). The applicative program (Recruitability-Assessment-APP) available at the mechanical ventilator performs the recruitability maneuver automatically. The APP provides gain of compliance percentage and shows the difference between pulmonary compliance at the same PEEPs. This study was realized using PEEP at 5 cmH_2_O and Ideal PEEP according of PEEP titration maneuver performed by electrical impedance tomography (EIT) and CT.

**Results:**

5 animals (38.7±2.8 kg) with severe lung injury (PaO_2_/FiO_2_<100 mmHg) were studied. By applying different lung-histories and PEEPs, we performed multiple maneuvers in the same animal, some of them applied to a lung full of collapse (with high-recruitability, confirmed by CT) versus some of them with an already-recruited-lung (animals with minimum recruitability, confirmed by CT). In the recruitable animals, the reduction in DP was (8.2 ± 4.8 cmH_2_O) versus (0.1 ± 0.1 cmH_2_O) in the non-recruitable ones. Respectively, the gain in compliance was (109.4% ± 40.6) in the recruitable ones vs. (5.6 % ± 4.8) in the non-recruitable ones (p=0.01). There was a significant correlation between the gain in compliance and the reduction in DP. (p=0.0002, R ^2^=0.83) (Figure1).

**Conclusions:**

Using a simple and automatized maneuver available at the bedside, it is possible to predict lung recruitability, as well as the consequent reduction in DP (> or < than 4 cmH_2_O, used as meaningful clinical threshold).

**Fig. 1 (abstract P576).**
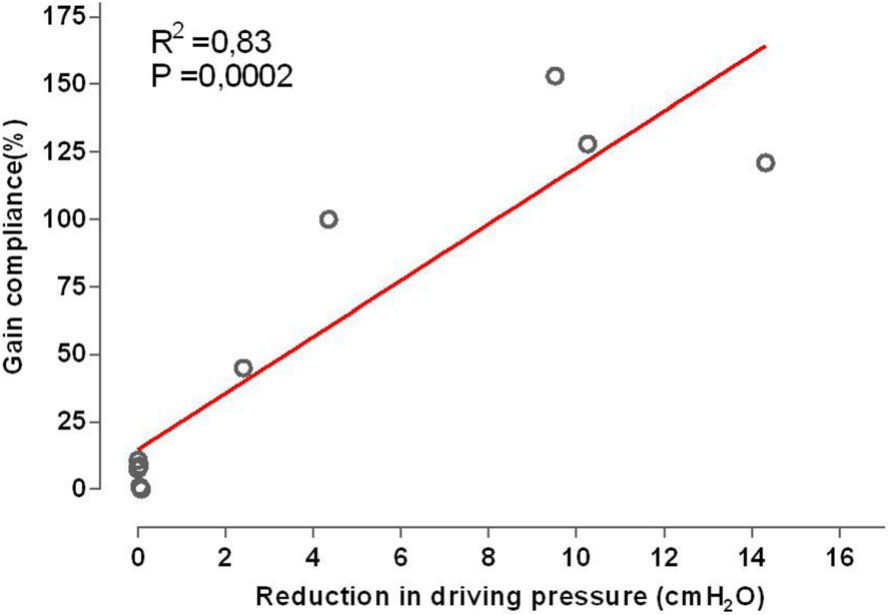
Regression linear model (gain of compliance- between the initial and final PEEP level – versus the DP (driving pressure) reduction observed after applying the final PEEP)

## P577 Cardiopulmonary exercise tests of intubated critically ill presented as 9-panel plots, a feasibility study

### M Kök^1^, H Van den Oever^1^, A Oosterwegel^2^, B Langeveld^3^

#### ^1^Intensive Care, Deventer Hospital, Deventer, Netherlands; ^2^Physical Therapy, Deventer Hospital, Deventer, Netherlands; ^3^Pulmonology, Deventer Hospital, Deventer, Netherlands

**Introduction:**

Critical illness may result in muscle weakness and decreased cardiopulmonary fitness. To avoid this, mobilization is started in the early stage of intensive care unit admission. Cardiopulmonary exercise testing can be used for diagnostic purposes. An accepted method of interpreting exercise data is by presenting them as 9-panel plots, as proposed by Wasserman [1], and analyzing them systematically. We set out to explore whether gas exchange data obtained during exercise in intubated, critically ill patients could be analyzed as standard 9-panel plots.

**Methods:**

Mechanically ventilated patients recovering from critical illness were subjected to an incremental exercise protocol using a bedside cycle ergometer (MOTOmed). Respiratory gases were analyzed with a Cosmed Quark, applied to an endotracheal tube. Blood gases were sampled from arterial catheters. Data were analyzed using MS Excel.

**Results:**

Exercise data of seven patients were analyzed individually, providing insight in their respiratory physiology. The cumulative data are shown in Figure 1. Basal metabolic rate was increased in 6/7 patients. Median oxygen uptake (VO_2_) increased from 408 to 489 ml/min during unloaded cycling, corresponding to an intrinsic workload of 10 W. Median maximum extrinsic workload during loaded cycling was 7 W, resulting in a median peak VO_2_ of 34.3 % of predicted VO_2_max. This was accompanied by an increase in CO_2_ production, respiratory minute volume and heart rate. Three patients passed the anaerobic threshold. Median fractional dead space at rest was 44%, decreasing to 42% during exercise, accompanied by a similar decrease in arterial-end tidal PCO_2_ difference, and improved respiratory efficiency for O_2_ and CO_2_. Median arterio-alveolar oxygen difference was 9.9 kPa, decreasing to 7.2 during exercise.

**Conclusions:**

This research demonstrated the feasibility of standard 9-panel plots to present and analyze exercise data in mechanically ventilated, critically ill patients.

**Reference**

1. Wasserman K et al. Principles of Exercise Testing and Interpretation, Wolters Kluwer, 2012

**Fig. 1 (abstract P577).**
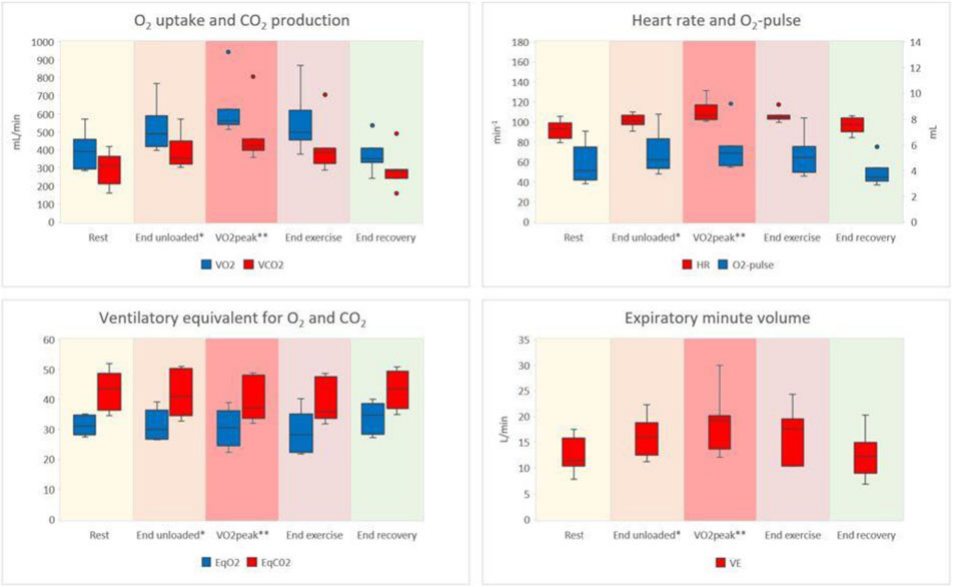
Exercise data of seven ventilated patients

## P578 Clinical evaluation of a wearable sensor for mobile monitoring of respiratory rate on hospital wards

### L Vikatmaa^1^, P Takala^2^, K Jarvela^3^, F Michard^4^

#### ^1^Anesthesia & Intensive Care, Helsinki University Hospital, Helsinki, Finland; ^2^GE HealthCare, R&D, Helsinki, Finland; ^3^GE HealthCare, Clinical Research, Helsinki, Finland; ^4^MiCo, Medical Research, Denens, Switzerland

**Introduction:**

Respiratory rate (RR) is a good predictor of adverse events on hospital wards. Capnography is the reference method to measure RR (RRcap), but it is not a mobile solution. A wireless wearable sensor was recently developed to monitor RR (RRw) in ambulatory patients. We assessed the feasibility of RR monitoring with the wearable sensor and compared RRw with RRcap in real ward conditions.

**Methods:**

The wearable sensor measures impedance variations of the chest from thoracic and abdominal electrodes. The respiratory signals are sent wirelessly to a smartphone-like mobile device that calculates and displays RRw and forward the value to a central station. Simultaneous measurements of RRw and RRcap (1 measure/minute) were compared in 36 ward patients (mean age 58 yrs). Patients were in bed when initiating monitoring but free to sit, speak, eat or move in their room when needed.

**Results:**

Patients were monitored for a period of 182 ± 56 (range 68-331) minutes. Non-artifacted RRcap and RRw measurements were available for 81% and 92% of the monitoring time, respectively (p<0.001). A total of 4836 pairs of simultaneous measurements were available for analysis. The average reference RRcap was 19 ± 5 breath/min (range 6-36). The average difference between RRw and RRcap was -0.6 ± 2.5 b/min. Error grid analysis showed that the proportions of RRw measurements were 89.7% in zone A (no risk), 9.6% in zone B (low risk) and <1% in zones C, D and E (moderate to dangerous risk). The wearable detected RRcap values >20 (tachypnea) with a sensitivity of 81% and a specificity of 93%.

**Conclusions:**

In ward patients, the wearable enabled accurate and precise measurements of RR within a broad range (6-36 b/min) and the detection of tachypnea with high sensitivity and specificity. It also enabled RR monitoring for a longer period of time than capnography. It has potential to facilitate nearly continuous monitoring of RR in ambulatory ward patients.

## P579 SpO_2_ - FiO_2_ diagram: useful and non-invasive tool for shunt monitoring in severe ARDS

### JF Martínez Carmona, S González Soto, MP Benitez, C Joya Montosa, MJ Delgado Amaya

#### Intensive Care Unit, Hospital Regional Universitario de Málaga, Málaga, Spain

**Introduction:**

Respiratory management of patients with moderate-severe ARDS remains challenging, regardless of cause. We must individualize management with each patient, and for this it is essential to have adequate respiratory monitoring to support decision-making. Sapsford and Jones described the SpO_2_ - FiO_2_ Diagram, a useful tool for bedside, which allows estimating the shunt and areas with low V / Q due to the closure of the small airway [1, 2].

**Methods:**

Male patient, 37 years old, with severe ARSD due to H1N1 influenza, requiring intubation in the first 24 hours of admission to the ICU. Protective ventilation is started, with a tidal volume of 7 cc/kg ideal weight, with RR to keep pCO_2_ in the normal range. A SpO_2_ - FiO_2_ Diagram is made after connection to MV, after performing lung recruitment maneuvers and after recruitment with prone position. For this, FiO_2_ is increased to 100% and drops of 10% are made every 2 minutes, evaluating the pulse oximetry value.

**Results:**

Protective ventilation is carried out, maintaining plateau pressure <30 cmh_2_o, driving pressure <15 at all times. AutoPEEP <2 cmH_2_O. After recruitment maneuvers, PEEP is titrated in 13 cmH_2_O for better static compliance. Initially, it requires 100% FiO_2_, which can be reduced to 60% in both RM and Prone Position. The initial PaO_2_ is 65mmHg, with a subsequent increase to 81 mmHg (post-MR) and 168 mmHg (after 2 hours of prone position). The increase in PaO_2_ / FiO_2_ is as follows: 65 - 135 - 281. Figure 1 shows the SpO_2_ - FiO_2_ diagram with the results obtained.

**Conclusions:**

Respiratory monitoring is key in the management of patients with severe ARDS, the SpO_2_ - FiO_2_ diagram is easy to perform and at bedside, providing useful information.

Consent to publish: written informed consent for publication was obtained from the patient.

**References**

1. Sapsford DJ and Jones JG. Eur J Anaesthesiol 12:375–86, 1995

2. Tusman G et al. Anesth Analg 124:62–71, 2017

**Fig. 1 (abstract P579).**
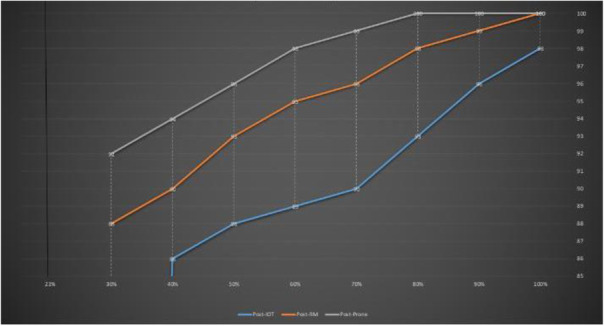
SpO_2_ - FiO_2_ Diagram

## P580 Mortality rates in mild versus severe abbreviated injury score (AIS) graded burns inhalation injury: a systematic review

### WN Charles^1^, A Dutt^2^, D Collins^3^, S Singh^4^

#### ^1^Department of Surgery and Cancer, Imperial College London, London, United Kingdom; ^2^Imperial College London, London, United Kingdom; ^3^Imperial College London and Department of Burns, Plastic and Reconstructive Surgery, Chelsea and Westminster Hospital NHS Foundation Trust, London, United Kingdom; ^4^Imperial College London and Magill Department of Anaesthesia, Intensive Care and Pain Management, Chelsea and Westminster Hospital NHS Foundation Trust and Royal Brompton and Harefield NHS Foundation Trust, London, United Kingdom

**Introduction:**

The Abbreviated Injury Score (AIS) grading system is the most widely used bronchoscopic classification for assessing the severity of burns inhalation injury. However, there is a lack of clarity regarding the effect of AIS-graded inhalation injury on mortality. This systematic review evaluated whether more severe injury grades were associated with increased mortality.

**Methods:**

OVID MEDLINE, EMBASE and CENTRAL were searched from inception to 5th April 2020. Clinical studies utilizing the AIS system to grade inhalation injury were deemed eligible if mortality data were reported by AIS grade. For comparison, AIS grades 0, 1 and 2 constituted a low-grade (milder) injury and AIS grades 3 and 4 constituted a high-grade (severe) injury. The level of evidence of each study was assessed as per the Oxford Centre for Evidence-Based Medicine guidelines. This systematic review adhered to the PRISMA statement.

**Results:**

The search identified 177 papers, of which 13 underwent full-text review. Six single-center, comparative studies (1 prospective and 5 retrospective designs) were included for descriptive analysis. The total number of patients was 715. Inter-grade differences in age and total body surface area burned were non-significant in five studies. Two studies demonstrated a statistically significant increase in mortality for more severe grades, with a further two demonstrating a non-significant trend (p ≤ 0.10). Five studies facilitated the comparison of low-grade (n=513) versus high-grade (n=122) inhalation injury. Mortality rates ranged from 13% (range 4-26%) in low-grade injuries to 27% (range 15-32%) in high-grade injuries. The use of AIS was inconsistent between studies, with all papers performing some further stratification to minimize clinician bias or for statistical purposes. The median level of evidence of included studies was 3.

**Conclusions:**

Mortality rates were higher in more severe AIS-graded burns inhalation injury. Refinement of the AIS is recommended to enable standardized use.

## P581 Comparison of cough efficacy between volume mode and pressure mode of mechanical insufflation-exsufflation in postextubated patients, a preliminary analysis

### S Klanarong^1^, N Kongpolprom^2^

#### ^1^Anesthesiology, King Chulalongkorn Memorial hospital, Bangkok, Thailand; ^2^Division of Pulmonary and Critical Care Medicine, Department of Medicine, Faculty of Medicine, King Chulalongkorn Memorial Hospital, Bangkok, Thailand

**Introduction:**

Mechanical insufflation-exsufflation (MI-E) possibly improves cough efficacy but there are limited data in terms of cough assist techniques, modes of device, safety and efficacy of MI-E in critically ill patients. So we want to compare cough efficacy (secretion clearance) and cough strength (maximum expiratory pressure; MEP), and safety between implementation of volume mode and pressure mode of MI-E in postextubated patients.

**Methods:**

We conducted a prospective crossover study. The postextubated patients with history of MV for ≥24 hours were enrolled. We excluded patients with severe COPD, lung bleb, pneumothorax, hemoptysis, recent history of thoracoabdominal surgery, increased intracranial pressure, impaired consciousness and pregnancy. The patients were randomized into 2 groups. The patients received cough assistance with either volume mode (group A) or pressure mode of MI-E (group B) of MI-E during the first period. The sputum volume, respiratory and hemodynamic parameters, and adverse events were recorded. One day after the first period, the patients received cough assistance with the other mode during the second period. The primary outcome was the amount of secretion after MI-E implementation and the secondary outcomes were MEP changes and adverse events.

**Results:**

Totally, 25 patients were enrolled. The majority of them were female, medical ICU patients with the mean age of 64 years. The amount of secretion after MI-E implementation was not significantly different between both modes. In addition, MEP, respiratory events and hemodynamic instability were also not significantly different between both modes. Remarkably, regardless of modes of MI-E, MEP tended to increase after multiple sessions of MI-E. Two patients were reintubated due to volume overload and vocal cord edema.

**Conclusions:**

In postextubated patients, cough efficacy after MI-E implementation was not different between volume and pressure modes. Additionally, MI-E was safe and tended to improve cough strength after extubation.

## P582 Knockdown of bone morphogenetic protein type II receptor leads to decreased aquaporin 1 expression and function in human pulmonary microvascular endothelial cells

### AG Vassiliou^1^, C Keskinidou^1^, A Kotanidou^2^, F Frantzeskaki^3^, I Dimopoulou^2^, D Langleben^4^, SE Orfanos^2^

#### ^1^National & Kapodistrian University of Athens, 1st Department of Critical Care Medicine & Pulmonary Services, GP Livanos and M Simou Laboratories, Athens, Greece; ^2^National & Kapodistrian University of Athens, First Department of Critical Care Medicine & Pulmonary Services, Athens, Greece; ^3^National & Kapodistrian University of Athens, Second Department of Critical Care, Athens, Greece; ^4^McGill University, Montreal, Canada

**Introduction:**

Pulmonary arterial hypertension (PAH) may lead to severe circulatory compromise, requiring intensive care. Bone morphogenetic proteins (BMPs) are known to have pivotal roles in organ diseases, including heritable PAH. In PAH, genetic mutations in the type II BMP receptor (BMPR2) are the most common cause of receptor dysfunction. However, it has also recently been demonstrated that aquaporin 1 (Aqp1) dysfunction, apart from its role in acute respiratory distress syndrome (ARDS), may contribute to PAH, highlighting that PAH development may involve more than one pathogenic pathway. Whether reduction in BMPR2 affects Aqp1 is unknown.

**Methods:**

We studied Aqp1 and the BMP-signalling molecules, SMADs, in *BMPR2*-silenced human pulmonary microvascular endothelial cells (HPMECs). mRNA levels were measured by RT-PCR, protein by immunoblotting, and Aqp1 function by permeability assays.

**Results:**

*BMPR2*-silenced HPMECs exhibited a reduced expression of Aqp1 at a mRNA and protein level. Moreover, *BMPR2*-silenced HPMECs showed reduced permeability function, implying dysfunctional Aqp1. *BMPR2-*silenced HPMECs also exhibited reduced expression of SMAD1/5/8 and SMAD2/3 pathways.

**Conclusions:**

Decreased *BMPR2* expression appears to affect Aqp1 at mRNA, protein, and functional levels. To our knowledge, this is the first report to demonstrate that decreased *BMPR2* gene expression leads to decreased Aqp1 expression and function in vitro.

## P583 Effects of lung recruitment in the prone position on dorsal lung aeration and lung oxygenation – a randomized controlled study on post cardiac surgery patients

### AM Martinsson

#### CardioThoracic Anaesthesia and Intensive Care, Sahlgrenska University Hospital, Göteborg, Sweden

**Introduction:**

Atelectasis in post cardiac surgery patients is more common compared to non-cardiac surgery, and may lead to ventilation/perfusion mismatch, infection, and an increase in ICU stay. A postoperative recruitment maneuver (RM) to increase aeration and lung oxygenation is clinical routine. In intensive care patients with severe respiratory failure, RM in the prone position may increase survival and/or oxygenation. The object was to compare prone to supine RM, regarding dorsal aeration and lung oxygenation in the extubated patient.

**Methods:**

A prospective randomized controlled trial in postoperative uncomplicated cardiac surgery patients. Subjects were randomized to RM in the prone or supine position, 15 patients in each group. The primary endpoints were ventilation distribution and end-expiratory lung volume measured by electrical impedance tomography and lung oxygenation, early after extubation.

**Results:**

The dorsal tidal volume in arbitrary units (AU) after extubation was 363 (CI 0.95: 283-443) and 212 (CI 0.95: 170-254) in the prone and supine group respectively, p<0.001, d=1.30 (Figure 1). The dorsal ∆EELV (AU) was 724 (CI 0.95: 456-992) and -163 (CI 0.95: -252- -73) in the prone and supine group respectively, p<0.001, d=2.46. The PaO_2_/FiO_2_ ratio after extubation was 46.6 (CI 0.95: 40.7-53.0) and 39.3 (CI 0.95: 34.8-43.8) in the prone and supine group respectively, p=0.041, d=0.74.

**Conclusions:**

Prone positioning plus RM in the prone position early after cardiac surgery is superior to supine RM, regarding dorsal aeration and lung oxygenation after extubation. This new beneficial recruitment strategy reduces dorsal atelectasis.

**Fig. 1 (abstract P583).**
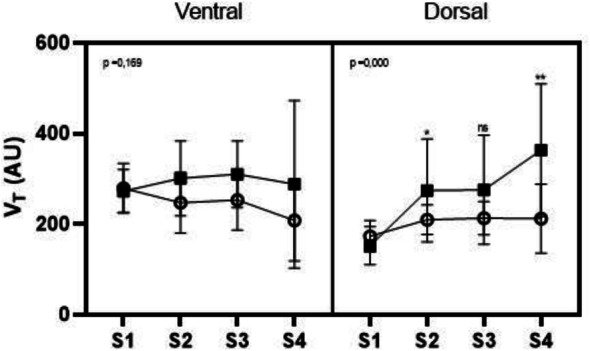
Tidal volume. Ventral: ventral regional tidal volume; Dorsal: dorsal regional tidal volume; S1-4: Supine Registration Point 1-4; AU: Arbitrary Unit; Filled boxes: Prone group; Open circles: Supine group

## P584 Non-operative treatment for tracheoesophageal fistulae in intensive care unit: our experience

### DM Palma^1^, AN Cracchiolo^2^, D Librizzi^3^, A La Sala^4^, L Serafino Agrusa^5^

#### ^1^AORNAS Civico, II Servizio Anestesia-Rianimazione, Palermo, Italy; ^2^AORNAS Civico, Anestesia e Rianimazione, Palermo, Italy; ^3^AORNAS Civico, Chirurgia Toracica, Palermo, Italy; ^4^AORNAS Civico, Endoscopia Bronchiale, Palermo, Italy; ^5^AORNAS Civico, Endoscopia Bronchiale, Palermo, Italy

**Introduction:**

The aim of our retrospective analysis is to evaluate the results of conservative management of acquired tracheoesophageal fistulae (TEF). TEF are rare but potentially life-threatening emergencies which can be of either spontaneous or iatrogenic origin. Spontaneous ones can be congenital or secondary to malignancy. For acquired ones numerous causes have been documented, the most common of which are endotracheal and tracheostomy tube-related injuries.

**Methods:**

From February 2017 to March 2020 seven patients (5 men; 2 women) with acquired TEF were diagnosed in our intensive care unit (ICU). The injury occurred after dilational percutaneous tracheostomy in 3 patients, after esophageal endoscopy in 1 patient, after cuff-related ruptures in the 3 intubated and mechanically ventilated patients. Our patients had no particular medical history. Mean age: 46 years. Mean duration of signs before diagnosis: 8 hours. All our patients had a Level III B lesion. The median length of the injury was 1.4 cm. The mean duration of hospitalization in the ICU was 31 days.

**Results:**

All patients underwent conservative management: antibiotic therapy, close bronchoscopic controls, percutaneous endoscopic gastrostomy and tomographic investigation. No mediastinitis was observed. Two patients died from causes unrelated to the tracheal injury.

**Conclusions:**

Successful management of acquired TEF requires a fast and straightforward diagnostic evaluation. According to our experience conservative management of TEF may be a save option in patients with uncomplicated ventilation and moderate and nonprogressive emphysema.

**Consent to publish:** Written informed consent for the publication of these details was obtained from the participants.

## P585 Use of volatile agents for sedation in the intensive care unit: a national survey in France

### R Blondonnet^1^, A Quinson^2^, C Lambert^3^, J Audard^1^, R Zhai^4^, B Pereira^3^, E Futier^1^, JE Bazin^2^, JM Constantin^5^, M Jabaudon^6^

#### ^1^CHU Clermont-Ferrand, Department of Perioperative Medicine, GReD, CNRS, INSERM, Clermont-Ferrand, France; ^2^CHU Clermont-Ferrand, Department of Perioperative Medicine, Clermont-Ferrand, France; ^3^Biostatistical and Data Management Unit, Department of Clinical Research and Innovation, Clermont-Ferrand, France; ^4^GReD, CNRS, INSERM, Clermont-Ferrand, France; ^5^Sorbonne University, GRC 29, AP-HP, DMU DREAM, Department of Anesthesiology and Critical Care, Pitié-Salpêtrière Hospital, Paris, France; ^6^Division of Allergy, Pulmonary, and Critical Care Medicine, Department of Medicine, Vanderbilt University Medical Center, Nashville, TN, United States

**Introduction:**

Current ICU sedation guidelines recommend strategies using non-benzodiazepine sedatives. Since the development of halogenated anesthetic reflectors, inhaled ICU sedation has become increasingly popular. This survey was undertaken to explore the use of volatile agents for ICU sedation in France.

**Methods:**

Adult ICUs of the Société Française d’Anesthésie et Réanimation (SFAR) database were contacted by phone or email between July and August 2019. Heads of ICU were questioned about the characteristics of their department, their knowledge on inhaled sedation, and practical aspects of inhaled sedation use in their department.

**Results:**

Among the 374 ICUs contacted, 187 provided responses (50%). Most ICU directors (73%) knew about the use of inhaled ICU sedation and 21% used inhaled sedation in their unit, mostly with the Anesthetic Conserving Device (AnaConDa, Sedana Medical). Most intensivists had used volatile agents for sedation for <5 years (63%) and in <20 patients per year (75%), with their main indications being: failure of intravenous sedation, severe asthma, and acute respiratory distress syndrome. Sevoflurane and isoflurane were mainly used (88% and 20%, respectively). The main reasons for not using inhaled ICU sedation were: “device not available” (40%), “lack of medical interest” (37%), “lack of familiarity or knowledge about the technique” (35%) and “elevated cost” (21%). Most respondents (80%) were overall satisfied with the use of inhaled sedation (Figure 1). Almost 75% stated that inhaled sedation was a seducing alternative to intravenous sedation.

**Conclusions:**

This survey highlights the widespread knowledge about inhaled ICU sedation in France but shows its limited use to date. Differences in education and knowledge, as well as the recent and relatively scarce literature on the use of volatile agents in the ICU, might explain the diverse practices that were observed. The low rate of mild adverse effects and the users’ satisfaction are promising for this potentially important tool for ICU sedation.

**Fig. 1 (abstract P585).**
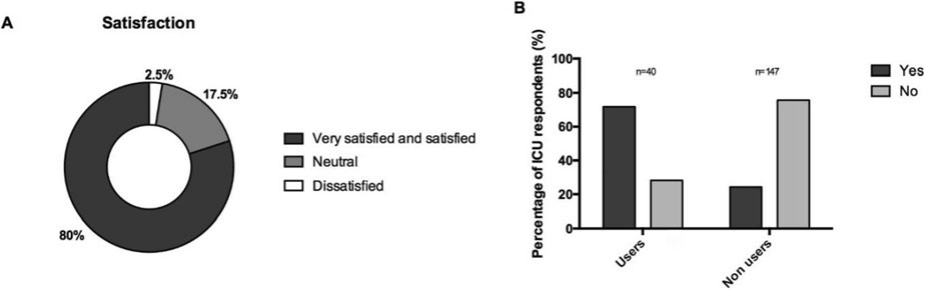
(A) Overall satisfaction of respondents regarding the use of inhaled sedation in their intensive care unit (n=40); (B) Answers to the question “Do you think that inhaled sedation is an interesting alternative to intravenous sedation in the intensive care unit?” by users and non-users of the technique (n=187). Data are shown in %

## P586 Delivery of the ‘Sepsis Six’ resuscitation bundle at a district general hospital

### K Moqeem, M Waseem Beeharry, F Bucknall, J Collis, D Cottam, W Gray, A Falinska, M Zuleika

#### Royal Surrey NHS Foundation Trust, Guildford, United Kingdom

**Introduction:**

Sepsis is a life-threatening organ dysfunction responsible for approximately 48,000 deaths per year in the UK [1]. The ‘Sepsis Six’ bundle consists of six practical components designed to enable rapid patient care. If initiated within the hour, it is associated with a 55% relative risk reduction in mortality and a reduced length of hospital stay and likelihood of critical care admission [1].

**Methods:**

A prospective observational study over four weeks in November 2019 to assess the adherence to ‘Sepsis Six’. Patients fulfilling the following inclusion criteria were added to a dedicated database:

1) Age ≥18

2) NEWS2 [2] ≥ 5 or NEWS2 ≥ 3 in a single category within 24 hours of admission

3) Evidence of clinical infection

The delivery of ‘Sepsis Six’ within one hour and subsequent patient outcomes were evaluated.

**Results:**

67 patients (mean age: 72 years; Age range: 20 to 95; Male: 57%; Female: 43%) met our inclusion criteria. Delivery of Sepsis Six within one hour was as follows - oxygen: 93%; antibiotics: 59%; i.v. fluids: 55%; blood cultures: 64%; lactate: 82%; urine output: 22%. Overall hospital mortality was 15%. Patient outcomes are outlined in Table 1. Delivery of antibiotics within an hour significantly reduced the variability in the length of hospital stay (p-value = 0.014). Initiation of i.v. fluids had no significant effect on the variability of the length of hospital stay.

**Conclusions:**

Nurse-led interventions (oxygen, blood cultures, lactate) had a higher delivery rate compared to those requiring prescriptions. We believe due to a small sample size significance of other outcomes was not elicited. We intend to re-audit with a larger study size after introducing interventions previously shown to improve outcomes. These include 'Sepsis Six' stickers, modifications to phlebotomy trolleys and training sessions to educate and empower staff working in acute settings [3,4].

**References**

1. UK Sepsis Trust, 2020

2. Royal College of Physicians. National Early Warning Score (NEWS) 2, 2017

3. Bentley et al. BMJ Qual Improv Rep 5:u206760.w3983, 2016

4. Kumar et al. BMJ Qual Improv Rep 4:u207871.w4032, 2015

**Table 1 (abstract P586).** Patient outcomes

	Antibiotics delivered within one hour: Yes	Antibiotics delivered within one hour: No	i.v. fluids initiated within one hour: Yes	i.v. fluids initiated within one hour: No
Mean length of hospital stay in days (SD)	8.75 (4.96)	11.63 (11.11)	9.73 (6.70)	10.29 (9.71)
ITU (% of admissions)	2.99	2.99	2.99	2.99
Died (%)	7.46	7.46	8.96	5.97

## P587 Immunomodulatory mechanisms of pentaglobin therapy in sepsis

### K Gericke^1^, S Weissmüller^2^, M König^2^, V Braun^1^, M Germer^3^

#### ^1^Biotest AG, Bioanalysis, Dreieich, Germany; ^2^Biotest AG, Translational Research, Dreieich, Germany; ^3^Biotest AG, Preclinical Research, Dreieich, Germany

**Introduction:**

In sepsis and septic shock, treatment with IgM-enriched human immunoglobulin preparations can reduce patient mortality and mechanical ventilation [1]. Elevated levels of pro- and anti-inflammatory cytokines are detected in the circulatory system of septic patients and correlate with survival. Excessive release of cytokines can be associated with immune dysregulation observed in sepsis. The observed large amount of anti-inflammatory cytokines is not sufficient to limit the negative effects of high levels of pro-inflammatory cytokines.

**Methods:**

Primary cells were used for experiments on modulating the production of inflammatory mediators by the IgM enriched human immunoglobulin preparation Pentaglobin *in vitro*. Commercial ligand binding assays were used for cytokine determinations. The binding of complement to antigen-antibody complexes was characterized by flow cytometry.

**Results:**

In sepsis, relevant pathogens can be neutralized by antibodies derived from human plasma. The production of the proinflammatory cytokines TNF-alpha, interleukin-6 and interleukin-1beta can be reduced by IgM-enriched human immunoglobulin preparations. Functionally relevant is the binding of the antibodies to their cellular receptors. IgM-enriched human immunoglobulin preparations bind the complement factor C1q better than normal IgG preparations and can thus efficiently initiate the innate immune response by activating the complement cascade. The interaction with complement is a critical link of various immunological mechanisms.

**Conclusions:**

IgM-enriched human immunoglobulin preparations can reduce pathogen-induced inflammatory mediators and have the potential to reduce the pathogen load in sepsis. Their immunomodulatory properties initiate favorable complement factor-mediated activities and limit excessive cytokine reactions.

**Reference**

1. Cui J et al. Ann Intensive Care 9:27, 2019

## P588 Procalcitonin: a tricky biomarker for an initial choice of appropriate ATB therapy!

### V Adamkova^1^, V Adamkova^2^, H Lahoda Brodska^3^

#### ^1^General University Hospital, Clinical Microbiology and ATB center, Prague, Czech Republic; ^2^Cardiff University, School of Biosciences, Cardiff, United Kingdom; ^3^General University Hospital, Clinical Biochemistry, Prague, Czech Republic

**Introduction:**

This study aimed to evaluate the appropriateness of the initial ATB therapy in S. pyogenes (GAS) sepsis (ie. use of proteosynthesis inhibitors (PI) in combination with betalactams) based on atypically high procalcitonin (PCT) concentrations. We hypothesized that PCT levels in GAS sepsis are unique within gram positive sepsis, resembling the values of gram-negative sepsis (GNS). Thus, we considered addition of PI in all cases of sepsis of unknown etiology with PCT higher than 3 [1,2] in the first 24 hours.

**Methods:**

Retrospective analysis of an initial choice of ATB in patients with GAS sepsis when acknowledged that high PCT concentrations might indicate the presence of GAS (n=16, hospitalized between 2019 and 2020) compared to routine praxis (n=46, 8-year observation period; 2010-2018). The hypothesis was based on an 8-year-long observation of extremely high PCT concentrations in patients with GAS sepsis. The relationship between appropriateness of the initial ATB therapy and high PCT values was tested by chi-squared contingency table test

**Results:**

During an 8-year observation period, appropriate initial ATB therapy was received by five out of 46 patients (11%) because the median PCT concentration in all evaluated patients was 12.41 ng/ml (IQR: 5.58-54.7 ng/ml) which, according to accepted cut-off PCT values, indicated GNS covering. When high values of PCT considered as potential indicator of GAS sepsis, the appropriateness of initial therapy was significantly increased (p<0.001), 11 out of 16 (69%) received appropriate initial ATB therapy, median PCT concentration was 25.1 ng/ml (IQR: 15.88-52.34 ng/ml).

**Conclusions:**

High concentrations of PCT are measured in patients with GAS sepsis and therefore, possible GAS etiology and use of PI should be considered when prescribing initial ATB therapy in septic patients with high PCT concentrations

**References**

1. Brodska H et al. Clin Exp Med 13:165–170, 2012

2. Li S et al. J Res Med Sci 21:39, 2016

## P589 Clinical phenotypes in sepsis: validation from a new randomized controlled clinical trial

### E Karakike^1^, M Roumpoutsou^1^, M Kyprianou^1^, K Psaroulis^2^, E Massa^3^, N Karampela^4^, A Pitsoulis^5^, P Chaloulis^6^, E Pappa^7^, EJ Giamarellos-Bourboulis^1^

#### ^1^National and Kapodistrian University of Athens, 4th Department of Internal Medicine, Athens, Greece; ^2^Aghios Dimitrios General Hospital, Intensive Care Unit, Thessaloniki, Greece; ^3^ Hippokration General Hospital, Intensive Care Unit, Thessaloniki, Greece; ^4^Korgialeneio Benakeio General Hospital, Intensive Care Unit, Athens, Greece; ^5^G. Gennimatas General Hospital, Intensive Care Unit, Thessaloniki, Greece; ^6^Theageneion General Hospital, Intensive Care Unit, Thessaloniki, Greece; ^7^Laiko General Hospital, Intensive Cate Unit, Athens, Greece

**Introduction:**

Sepsis is characterized by substantial heterogeneity; recently, a large observational study (SENECA) clustered over 60000 septic patients into 4 distinct clinical phenotypes (α, β, γ and δ) that predict 28-day mortality and can be used for enrichment in clinical studies [1]. We aimed to identify those phenotypes in a new multi-center randomized, clinical trial (RCT), evaluating clarithromycin as immune modulator (INCLASS study, NCT 03345992).

**Methods:**

The INCLASS study included adult patients with sepsis, respiratory failure and multiple organ dysfunction. Among the 29 variables, used for clustering in the original publication, we employed 6 (serum creatinine, lactate, aspartate aminotransferase-AST, bilirubin, CRP and INR), prioritizing those that were most instrumental in distinguishing the four phenotypes. For those, the median values of the SENECA cohorts were set as centroids for each phenotype. The Euclidian distances were calculated for every patient from all centroids, providing a likelihood of phenotype assignment.

**Results:**

Preliminary data from 104 patients are shown. Despite of the high severity of the INCLASS cohort, (mean SOFA 10.7 ± 2.4,APACHE II 21 ± 6.8, 28-day mortality 47.1%), all 4 phenotypes were present (α 22%, β 23%, γ 30%, δ 25%). The main characteristics of phenotypes (Table 1) were preserved, median and range values were similar to the SENECA cohort; phenotype α was associated with lower mortality, β with higher age and comorbidity burden, δ with hepato-biliary dysfunction and higher lactate, compared to other phenotypes (p<0.001).

**Conclusions:**

This is an example of a simple operational algorithm successfully classifying patients from a new RCT in phenotypes derived from SENECA database. Whether the study intervention impacts differently those phenotypes is the next step of the analysis.

**Reference**

1. Seymour CW et al. JAMA 321:2003-17, 2019

**Table 1 (abstract P589).** The main characteristics of the phenotypes. IQR: interquartile range, CCI: Charlson comorbidity index, CRP: C-reactive protein, AST: Aspartate aminotransferase

	α	β	γ	δ
Age, median (IQR)	76 (52-80)	77 (71-85)	74 (65-80)	70 (59-76)
CCI, median (IQR)	5 (2-6)	6 (3-8)	6 (4-8)	5 (3-7)
Creatinine, median (IQR)	0.9 (0.6-1.3)	2.3 (1.5-2.9)	1.4 (0.8-2.7)	1.7 (1.0-3.2)
Lactate, median (IQR)	1.4 (0.9-2.1)	1.2 (1.1-2.1)	1.9 (1.7-2.4)	3.3 (2.3-4.1)
CRP, median (IQR)	48 (22-141)	136 (78-254)	168 (113-281)	169 (84-278)
AST, median (IQR)	33 (19-42)	27 (23-46)	31 (20-45)	92 (58-209)
Hospital mortality, n %	11 (50)	13 (56.5)	19 (63.3)	21 (80.8)

